# Shared Decision-Making in Colorectal Cancer Screening for Older Adults: A Secondary Analysis of a Cluster Randomized Clinical Trial

**DOI:** 10.1001/jamanetworkopen.2024.29645

**Published:** 2024-08-23

**Authors:** Karen R. Sepucha, Yuchiao Chang, K.D. Valentine, Steven J. Atlas, Paul K. J. Han, Lauren J. Leavitt, Brittney Mancini, James M. Richter, Lydia C. Siegel, Kathleen M. Fairfield, Leigh H. Simmons

**Affiliations:** 1 Division of General Internal Medicine, Massachusetts General Hospital, Boston; 2 Harvard Medical School, Boston, Massachusetts; 3 Center for Interdisciplinary Population and Health Research, MaineHealth Institute for Research, Portland; 4 Division of Cancer Control and Population Sciences, National Cancer Institute, Bethesda, Maryland; 5 Division of Gastroenterology, Massachusetts General Hospital, Boston; 6 Division of General Internal Medicine, Brigham and Women’s Hospital, Boston, Massachusetts

## Abstract

**Importance:**

Decisions about whether to stop colorectal cancer (CRC) screening tests in older adults can be difficult and may benefit from shared decision-making (SDM).

**Objective:**

To evaluate the effect of physician training in SDM and electronic previsit reminders (intervention) vs reminders only (comparator) on receipt of the patient-preferred approach to CRC screening and on overall CRC screening rates of older adults at 12 months.

**Design, Setting, and Participants:**

This was a secondary analysis of the Promoting Informed Decisions About Colorectal Cancer Screening in Older Adults (PRIMED) cluster randomized clinical trial. In the PRIMED trial, primary care physicians (PCPs) from 36 primary care practices in Massachusetts and Maine were enrolled between May 1 and August 30, 2019, and were randomized to the intervention group or the comparator group. Patients aged 76 to 85 years who were overdue for CRC screening and did not have a prior diagnosis of CRC enrolled between October 21, 2019, and April 8, 2021. Data analysis was performed between May 24, 2022, and May 10, 2023.

**Interventions:**

Primary care physicians in the intervention group completed an SDM training course and received previsit reminders of patients eligible for CRC testing discussion, whereas PCPs in the comparator group received reminders only.

**Main Outcomes and Measures:**

The primary outcome was concordance, or the percentage of patients who received their preferred screening approach. Postvisit surveys were administered to assess patient preference for testing, and electronic health record review was used to assess CRC testing at 12 months. Heterogeneity of treatment effect analyses examined interaction between study groups and different factors on concordance rates.

**Results:**

This study included 59 physicians and 466 older adults. Physicians had a mean (SD) age of 52.7 (9.4) years and a mean (SD) of 21.6 (10.2) years in practice; 30 (50.8%) were women and 16 (27.1%) reported prior training in SDM. Patients had a mean (SD) age of 80.3 (2.8) years; 249 (53.4%) were women and 238 (51.1%) reported excellent or very good overall health. Patients preferred stool-based tests (161 [34.5%]), followed by colonoscopy (116 [24.8%]) or no further screening (97 [20.8%]); 75 (16.1%) were not sure. The distribution of patient preferences was similar across groups (*P* = .36). At 12 months, test uptake was also similar for both the intervention group (29 [12.3%] for colonoscopy, 62 [26.3%] for stool-based tests, and 145 [61.4%] for no testing) and the comparator group (32 [13.9%] for colonoscopy, 35 [15.2%] for stool-based tests, and 163 [70.9%] for no testing; *P* = .08). Approximately half of patients in the intervention group received their preferred approach vs the comparator group (115 of 226 [50.9%] vs 103 of 223 [46.2%]; *P* = .47). Heterogeneity of treatment effect analyses found significantly higher rates with the intervention vs the comparator for patients with a strong intention to follow through with the preferred approach (adjusted odds ratio [AOR], 1.79 [95% CI, 1.11-2.89]; *P* = .02, *P* = .05 for interaction) and for patients who reported more than 5 minutes (AOR, 3.27 [95% CI, 1.25-8.59]; *P* = .02, *P* = .05 for interaction) of discussion with their PCP regarding screening. Higher rates were also observed among patients who reported 2 to 5 minutes of discussion with their PCP, although this finding was not significant (AOR, 1.89 [95% CI, 0.93-3.84]; *P* = .08, *P* = .05 for interaction).

**Conclusions and Relevance:**

In this secondary analysis of a cluster randomized clinical trial, approximately half of older patients received their preferred approach to CRC screening. Physician training in SDM did not result in higher concordance rates overall but may have benefitted some subgroups. Future work to refine and evaluate clinical decision support (in the form of an electronic advisory or reminder) as well as focused SDM skills training for PCPs may promote high-quality, preference-concordant decisions about CRC testing for older adults.

**Trial Registration:**

ClinicalTrials.gov Identifier: NCT03959696

## Key Points

**Question:**

Does physician training in shared decision-making (SDM) improve receipt of older patients’ preferred approaches to colorectal cancer (CRC) screening?

**Findings:**

In this cluster randomized clinical trial with 59 physicians and 466 older adults, 39% of patients in the intervention group and 29% in the comparator group completed CRC screening in the 12 months after the index visit, a nonsignificant difference. Approximately 51% of patients in the intervention group received their preferred approach to screening compared with 46% in the comparator group (nonsignificant difference).

**Meaning:**

Approximately half of the older patients in this study received their preferred approach to CRC screening, but these findings indicate that SDM training did not have a meaningful effect on concordance rates or screening rates.

## Teaser

This secondary analysis of a cluster randomized clinical trial evaluates the effect of physician training in shared decision-making on receipt of the patient-preferred approach to colorectal cancer screening among older adults.

## Introduction

Decision-making about colorectal cancer (CRC) screening and surveillance after age 75 years can be difficult. Even for patients with a history of colon polyps, continued testing after age 75 years is not a straightforward decision. They may continue with colonoscopy, switch to stool-based testing, or stop screening altogether. Patients and clinicians need to balance the potential benefits of screening, based on CRC risk and life expectancy, with the potential risks and complications. The need for shared decision-making (SDM) is reflected in guidelines from the US Preventive Services Task Force^1^ and the US Multi-Society Task Force on Colorectal Cancer^2^ that advise individualized decision-making for CRC screening and surveillance for people aged 76 to 85 years. Despite the recommendation for an individualized decision, a study of older patients found that only half reported that the topic of whether to continue or stop testing for CRC was ever discussed.^3^

Physicians caring for older patients often need to consider whether medical interventions, such as cancer screening, should be modified or stopped. To date, only a few studies have examined patient education and SDM tools to promote deimplementation of tests or treatments.^4,5^ Deimplementation refers to strategies that are focused on discontinuing or reducing the use of interventions that are not effective, are not appropriate, or are harmful. For patients aged 76 to 85 years, the goal is not to stop all screening; however, insights from the deimplementation paradigm, including how beliefs, attitudes, and social norms may affect the acceptability of options, may be helpful for SDM. For example, studies have reported that patients may be reluctant to forgo screening, even if it is unlikely to provide any meaningful benefit, because screening is associated with being healthy and a good patient.^6,7^ Some studies suggest that patients perceive deimplementation discussions as withholding care, which may decrease confidence in or lower satisfaction with their health care clinician.^8^ In addition, physicians’ experiences, particularly negative experiences, shape their beliefs and attitudes and may affect their receptivity to initiating a discussion about stopping cancer screening.^8,9,10^ There are considerable gaps in our understanding of how to best support effective SDM conversations about stopping or reducing the intensity of interventions.

The Promoting Informed Decisions About Colorectal Cancer Screening in Older Adults (PRIMED) study evaluated the effect of an online SDM training course for primary care physicians (PCPs) plus electronic reminders sent before visits with older adults who were due for discussion about CRC screening or surveillance testing.^11^ The SDM skills training explicitly acknowledged the challenges inherent in this situation and provided evidence-based scripts designed to mitigate potential negative patient reactions to the option of stopping CRC testing. The training focused on 3 options: stopping testing entirely, switching to less invasive stool-based testing, and continuing colonoscopy. As reported previously, the PRIMED investigators found that patients seen by physicians in the training group reported more frequent discussions about CRC testing, had higher SDM scores, and expressed greater intentions to follow through with their preferred approach.^11^ Importantly, despite the more frequent discussions about CRC testing in the intervention group, patient satisfaction was similar across study groups.

The purpose of this planned secondary analysis was to test the a priori hypothesis that more patients seen by PCPs in the intervention group would have higher concordance (defined as the percentage who followed through with their preferred screening modality) compared with patients seen by PCPs in the comparator group. We also explored the effect of the interventions on overall CRC testing rates between groups.

## Methods

This was a secondary analysis of the multisite PRIMED cluster randomized clinical trial (NCT03959696), which is described in detail in the primary publication.^11^ Single institutional review board approval was granted by Mass General Brigham, and the full study protocol is provided in Supplement 1. Physicians and patients provided informed consent. Four patient advisors provided feedback on the study design, outreach to patients, implementation of the study protocol, and the outcome measures. This article followed the Consolidated Standards of Reporting Trials (CONSORT) and the SPIRIT Extension for RCTs Revised in Extenuating Circumstances (CONSERVE) reporting guidelines.

### Study Procedures and Interventions

#### Physicians

We enrolled physicians between May 1 and August 30, 2019. Randomization to the intervention or comparator occurred at the physician level (Figure). Physicians were from 36 internal medicine and family medicine practices (with an average of 1.6 physicians per practice) affiliated with 3 academic centers and 2 community hospitals in Massachusetts and Maine that had at least 20 potentially eligible patients aged 76 to 85 years in their panel. Eligible physician participants were grouped into strata based on self-reported sex (male or female), self-reported prior exposure to SDM training (yes, no, or not sure), and site. Within each stratum, years in practice was sorted in ascending order to create blocks each with a size of 2. The statistician (Y.C.) then assigned physicians to 1 of 2 groups (intervention or comparator) using a random number generator within each block.

**Figure.**
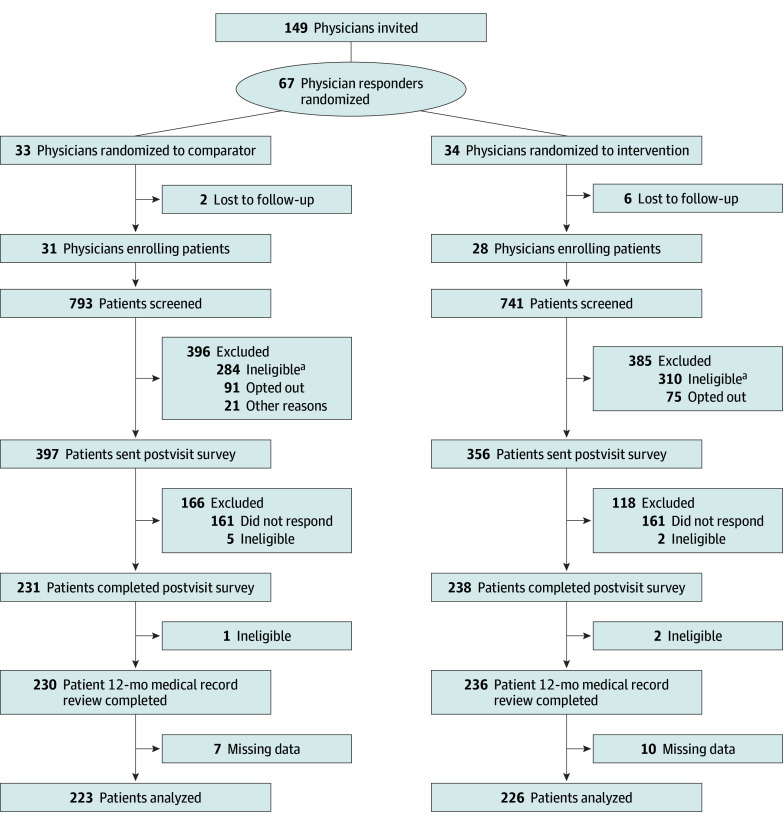
Study Flow Diagram ^a^Reasons for ineligibility included the following: being up to date on screening; having impaired cognition, genetic disorders, or past diagnosis of colorectal cancer; being deceased; not attending a visit or visits being suspended due to the COVID-19 pandemic; and other reasons.

Physicians in the intervention group completed a 2-hour online SDM skills course built around 4 different cases that highlighted SDM for decisions about whether to discontinue CRC testing for older adults. Physicians also received electronic reminders whenever a patient with an upcoming visit (the index visit) was eligible for a discussion about CRC testing. The reminder encouraged physicians to discuss CRC testing and included information about the patient’s most recent CRC test and date, when available. A detailed course description and sample reminder text is included in eTable 1 in Supplement 2. Physicians in the comparator group received the electronic reminders only. Participating physicians were unaware of the interventions used in the other group and were unaware of the study hypotheses.

#### Patients

Patient enrollment occurred between October 21, 2019, and April 8, 2021. After completing a visit with a study physician, eligible patients were sent a survey to complete (which captured patient CRC testing preference). Patients indicated consent by return of the survey. Patients were included in this analysis if they were aged 76 to 85 years and met the following criteria: had no prior diagnosis of CRC, had no prior colectomy, were due for discussion about CRC screening or surveillance, had a visit with a participating physician during the study period, and completed the postvisit survey. Patients were blinded to the intervention.

Study enrollment was suspended between March 13 and May 26, 2020. The primary institution halted all minimal-risk research studies due to the COVID-19 pandemic. The study restarted as soon as the study primary investigators, site primary investigators, and statistician determined it was feasible. Routine primary care visits were canceled, postponed, or moved to virtual visits at high rates for several months after reopening. No major changes to the protocol, outcomes, or analyses were made. Study staff tracked whether visits were in person or virtual (all visits before March 13, 2020, were in person) and added exploratory analyses to examine the effect of timing on outcomes.

### Measures

This analysis used a subset of items from the postvisit patient survey. These items included the patient-preferred approach to screening (colonoscopy, stool-based test, no screening, other, or not sure), strength of patient intention to follow through with the preferred screening approach (“definitely will follow through” vs other responses), discussion of CRC testing (yes or no, and time spent discussing), demographic characteristics (race and ethnicity, education, employment, and marital status), health status (score on the Patient-Reported Outcomes Measurement Information System [PROMIS] Scale, version 1.2—Global Health Physical 2a^12^), health literacy,^13^ and CRC risk factors (family history of CRC, prior screening, or prior polyps).

Data on patient race and ethnicity were collected per requirements of the funder and the data were reported to characterize the sample and help the reader assess generalizability. Race was reported as American Indian or Alaska Native, Asian, Black or African American (hereinafter, Black), Native Hawaiian or Other Pacific Islander, White, other race or multiple races, or unknown. Ethnicity was reported as Hispanic, non-Hispanic, or unknown.

Study staff extracted data from the electronic health record on patient sex, use of CRC screening, and surveillance test results (eg, colonoscopy or stool-based tests) for enrolled participants in the 12-month period after the index visit. Research staff who entered survey data and the biostatistician who analyzed the data were blinded to the intervention.

### Statistical Analysis

#### Primary Analysis

The PRIMED primary analysis compared respondents and nonrespondents, and the investigators found that nonrespondents were slightly older, more likely to be men, and less likely to have had a prior screening test than responders (eTable 2 in Supplement 2).^11^ In the primary analysis, sample demographics and characteristics were also compared to evaluate balance between the 2 patient groups. The intervention group had a significantly higher percentage of female participants and a significantly higher percentage of participants with a history of prior CRC screening.^11^ Subsequent analyses adjusted for these imbalances according to our prespecified analysis plan.

For patient-reported outcomes, our analyses excluded patients with missing data. Distribution of preferences was compared using a χ^2^ test between study groups. We calculated concordance by comparing patient-reported testing preference with documentation of completed testing following the simplified concordance scoring methods for the decision quality instruments.^14,15^ If there was a match (eg, patient indicated preference for colonoscopy and received colonoscopy), then we considered the patient preference concordant. All mismatches were considered preference discordant, including all patients who selected “not sure” for their preference. The prespecified analyses were evaluated using an intention-to-treat approach in which patients were analyzed based on their assigned group, regardless of whether the intervention took place (ie, clinician completed the training, received the notification, or discussed CRC screening in the visit).

This analysis examined the prespecified hypothesis that a higher percentage of patients would receive preference-concordant care in the intervention group. We compared the percentages of patients who received their preferred testing in the 12 months after the index visit between the 2 groups using a logistic regression model with the generalized estimating equation (GEE) approach to adjust for clustering of patients within clinicians. For the 59 participating PCPs recruited from 36 practices (with an average of 1.6 physicians per practice), the intracluster correlation coefficient was 0.02 at the physician level and 0.025 at the clinic level. Two-sided *P* ≤ .05 was considered statistically significant for all analyses.

The original calculation anticipated a sample size of 500 patients. For the concordance analysis, we had 226 patients in the intervention group and 223 in the comparator group. With an average cluster size of 8 and assuming a 46.2% concordance rate in the comparator group, the study would have 80% power to detect the 12.5% difference.

#### Exploratory Analysis

We compared CRC testing rates between study groups. We did not have an a priori hypothesis about whether testing rates of the intervention group would be higher or lower than those of the comparator group. Logistic regression models with the GEE approach were used to test the interaction between study groups and screening rate.

We also explored the heterogeneity of treatment effects by examining the interaction between study groups and different factors on rates of preference concordance. The factors established a priori included the following: (1) physician sex, (2) years of physician experience, (3) hospital network, (4) patient sex, (5) patient age, (6) prior screening history (including test type, prior polyps, and family history), (7) strength of follow-through intentions, (8) discussion of CRC and time spent discussing CRC, (9) patient preference, (10) physician recommendation, and (11) overall patient health. Logistic regression models with the GEE approach were used to test the interaction between study groups and these factors.

Statistical analysis was conducted with SAS, version 9.4 (SAS Institute Inc). Data analysis was performed between May 24, 2022, and May 10, 2023.

## Results

The 59 participating PCPs had a mean (SD) age of 52.7 (9.4) years; 30 (50.8%) were women and 29 (49.2%) were men. Physicians had a mean (SD) of 21.6 (10.2) years in practice, and 16 (27.1%) reported prior training in SDM. No differences in physician characteristics were found between groups. Physician characteristics are presented in eTable 3 in Supplement 2. The 466 patients had a mean (SD) age of 80.3 (2.8) years; 249 (53.4%) were women and 217 (46.6%) were men. Patient characteristics are presented in Table 1. Slightly more than half of patients (238 [51.1%]) self-reported excellent or very good overall health. With regard to patient race, 5 (1.1%) were Asian, 8 (1.7%) were Black, 436 (93.6%) were White, and 6 (1.3%) were of other race or multiple races; race was unknown for 11 (2.4%). With regard to patient ethnicity, 6 (1.3%) were Hispanic and 451 (96.8%) were non-Hispanic; ethnicity was unknown for 9 (1.9%).

**Table 1.** Patient Characteristics^a^

Characteristic	Group (N = 466)	
	Intervention (n = 236)	Comparator (n = 230)
Age, mean (SD), y	80.4 (2.7)	80.1 (2.8)
Sex		
Female	138 (58.5)	111 (48.3)
Male	98 (41.5)	119 (51.7)
Race		
American Indian or Alaska Native	0	0
Asian	4 (1.7)	1 (0.4)
Black or African American	4 (1.7)	4 (1.7)
Native Hawaiian or Other Pacific Islander	0	0
White	220 (93.2)	216 (93.9)
Other or multiple	3 (1.3)	3 (1.3)
Unknown	5 (2.1)	6 (2.6)
Ethnicity		
Hispanic	4 (1.7)	2 (0.9)
Non-Hispanic	229 (97.0)	222 (96.5)
Unknown	3 (1.3)	6 (2.6)
Education		
High school graduate or less	63 (26.7)	65 (28.3)
Some college or 2-y degree	39 (16.5)	53 (23.0)
College graduate (4-y degree)	42 (17.8)	40 (17.4)
More than 4-y degree	89 (37.7)	68 (29.6)
Prior screening test		
Colonoscopy	159 (67.4)	152 (66.1)
Stool-based test	48 (20.3)	33 (14.3)
None on record	29 (12.3)	45 (19.6)
Family history of colorectal cancer	47 (20.3)	42 (19.0)
Prior polyps removed	115 (50.7)	107 (47.6)
Marital status		
Married or living with partner	135 (57.2)	136 (59.1)
Widowed	54 (22.9)	47 (20.4)
Separated or divorced	32 (13.6)	25 (10.9)
Single (never married)	11 (4.7)	14 (6.1)
Other or missing	4 (1.7)	8 (3.5)
High health literacy	198 (85.3)	189 (82.5)
Health status (excellent or very good)	123 (53.7)	115 (50.7)

^a^ Unless specified otherwise, values are presented as No. (%) of patients.

Among the 466 patients, 161 (34.5%) preferred stool-based testing, 116 (24.8%) preferred colonoscopy, 97 (20.8%) preferred no further screening, and 75 (16.1%) were unsure. The remaining 17 (3.6%) did not provide a clear preference and were excluded from the concordance analyses. Distribution of preferences was similar across the 2 study groups (*P* = .36).

Of the 236 patients in the intervention group, 145 (61.4%) received no testing, 62 (26.3%) completed stool-based testing, and 29 (12.3%) completed colonoscopy. Of the 230 patients in the comparator group, 163 (70.9%) received no testing, 35 (15.2%) completed stool-based testing, and 32 (13.9%) completed colonoscopy. Screening test completion was not significantly different between groups after adjusting for covariates and clustering of patients within physicians (*P* = .08).

Approximately half of the 466 patients (218 of 449 [48.6%]) received their preferred approach to screening. Although the intervention group had a higher concordance rate than the comparator group, the difference (4.7%) was not statistically significant (115 of 226 [50.9%] vs 103 of 223 [46.2%]; adjusted odds ratio [AOR], 1.17 [95% CI, 0.78-1.78]; *P* = .47) (Table 2).

**Table 2.** Concordance Rates by Patient Preferences and Study Group

Patient preference	Received preferred approach, No./No. (%)		
	Intervention group	Comparator group	Total
Colonoscopy	25/57 (43.9)	26/59 (44.1)	51/116 (44.0)
Stool-based test	48/90 (53.3)	27/71 (38.0)	75/161 (46.6)
No further screening	42/46 (91.3)	50/51 (98.0)	92/97 (94.8)
Not sure	0/33 (0)	0/42 (0)	0/75 (0)
Overall concordance by group	115/226 (50.9)^a^	103/223 (46.2)^b^	NA

Abbreviation: NA, not applicable.

^a^ Excludes 10 patients who were missing the preference item.

^b^ Excludes 7 patients who were missing the preference item.

In the planned heterogeneity analyses for concordance, patients who reported that their physician spent more than 5 minutes (AOR, 3.27 [95% CI, 1.25-8.59]; *P* = .02, *P* = .05 for interaction) talking about CRC screening and those who indicated strong follow-through intentions (AOR, 1.79 [95% CI, 1.11-2.89]; *P* = .02, *P* = .05 for interaction) had significantly higher concordance rates in the intervention group vs the comparator group. Table 3 presents the results of heterogeneity analyses related to concordance.

**Table 3.** Heterogeneity of Treatment Effects Analyses for Preference-Concordant Testing

Variable	AOR (95% CI)	*P* value^a^	*P* value for interaction^b^
Patient age, y			
<80	1.38 (0.84-2.28)	.21	.40
≥80	0.96 (0.56-1.65)	.88	
Patient sex			
Female	1.02 (0.55-1.91)	.94	.49
Male	1.28 (0.72-2.28)	.39	
Health status			
Excellent or very good	1.19 (0.72-1.98)	.50	.92
Good, fair, or poor	1.15 (0.64-2.07)	.63	
Prior CRC test			
Colonoscopy or other procedure	0.98 (0.59-1.61)	.93	.28
Stool-based test	2.77 (0.98-7.88)	.06	
None	1.68 (0.56-5.03)	.35	
Family history of CRC			
No	1.13 (0.52-2.46)	.76	.79
Yes	1.15 (0.70-1.89)	.58	
Prior polyp			
No	1.03 (0.60-1.77)	.91	.55
Yes	1.30 (0.77-2.21)	.32	
Physician age, y			
<55	1.07 (0.66-1.74)	.78	.83
≥55	1.17 (0.54-2.53)	.69	
Physician sex			
Female	1.20 (0.62-2.32)	.60	.97
Male	1.07 (0.60-1.92)	.81	
Hospital site			
1	1.11 (0.51-2.39)	.79	.43
2	0.29 (0.14-0.60)	<.001	
3	1.74 (0.55-5.49)	.34	
4	0.89 (0.35-2.25)	.80	
5	0.85 (0.52-1.38)	.51	
Physician experience, y			
<25	1.24 (0.69-2.22)	.48	.88
≥25	1.09 (0.53-2.26)	.81	
Discussed CRC testing in visit			
No	0.57 (0.26-1.28)	.17	.15
Yes	1.27 (0.79-2.03)	.32	
Time spent discussing CRC, min			
0	0.57 (0.26-1.28)	.17	.05
<2	0.27 (0.07-1.03)	.06	
2-5	1.89 (0.93-3.84)	.08	
>5	3.27 (1.25-8.59)	.02	
Patient preference			
Colonoscopy	1.16 (0.56-2.40)	.69	.28
Stool-based test	1.91 (0.86-4.23)	.11	
No further testing	0.51 (0.05-5.13)	.57	
Patient intention to follow through with preferred approach			
Definitely yes	1.79 (1.11-2.89)	.02	.05
Other response	0.65 (0.36-1.19)	.16	
Physician recommendation			
Colonoscopy	1.20 (0.60-2.39)	.60	.18
No further testing	0.15 (0.01-2.05)	.16	
Stool-based test	1.15 (0.35-3.77)	.82	
No recommendation	2.89 (0.88-9.51)	.08	

Abbreviations: AOR, adjusted odds ratio; CRC, colorectal cancer.

^a^ For subgroup.

^b^ For the interaction between intervention groups and subgroups.

## Discussion

This secondary analysis examined the effect of electronic reminders with and without physician SDM training on patient receipt of preferred CRC testing and on testing uptake among older adults. At 12 months after the index visit, relatively few patients had undergone any type of screening and approximately half had received their preferred approach. The SDM training did not result in statistically higher concordance with the preferred screening approach compared with reminders only. Screening rates were fairly low overall and were not statistically different between groups. Selected subgroups seemed to benefit, including patients who had strong follow-through intentions and patients who reported having a longer discussion (>5 minutes) with their PCP.

A key goal of SDM is to help patients clarify their preferences and then ensure that patients receive their preferred approach to care. Although the overall effect of SDM training on concordance rates was nonsignificant in this study, these findings are an important contribution to the minimal published literature in this area. For example, in a Cochrane review of patient decisions aids, only 10 of the 105 included randomized trials reported on concordance; the 10 studies (N = 4626) found a positive effect (risk ratio, 2.06 [95% CI, 1.46-2.91]).^16^ In a different Cochrane review of interventions to promote SDM (eg, physician training, feedback, and reminders), 2 of the 87 included trials reported on concordance; neither trial found a significant effect of SDM interventions.^17^ It may be that combining patient-facing and clinician-facing interventions (eg, training plus patient decision aids), incorporating SDM skills earlier in clinical training, or requiring SDM skills as part of recertification would be more effective. More research is needed to identify best approaches to ensure patients receive their preferred approach.

The focus of this study was to promote SDM in a situation in which patients and physicians may be considering reducing intensity of testing or stopping testing. For patients aged 76 to 85 years, CRC screening generally has increasing harms and decreasing benefit, but where or when the harms outweigh the benefits is not clear. Thus, there is a role for SDM to share options, review goals, and discuss trade-offs. There may also be a role for the deimplementation literature to provide insights regarding strategies to overcome barriers to stopping interventions. For example, the SDM training took insights from the deimplementation literature to distinguish between removal (stopping a screening altogether) or reduction (lowering intervention intensity through switching to stool-based testing)—both common deimplementation strategies.^8^ Deimplementation also promotes the use of risk-based strategies to identify individuals for whom the intervention should not be used. Although the SDM training had PCPs use risk calculators to estimate CRC risk and prognosis, the training did not promote explicit risk-based strategies (eg, patients with 10-year median life expectancy should not get colonoscopy).

The planned heterogeneity analyses identified 2 groups that seemed to receive substantial benefit from the intervention over the comparator in terms of higher concordance rates. The first group was patients with a strong intention to follow through with their preferred approach to testing. Studies have consistently found evidence that intentions are strong predictors of cancer screening behaviors.^18^ The SDM skills training may have helped PCPs in the intervention group elicit preferences for those patients with strong intentions and provide them more support to follow through. For patients with weak intentions, the training was no more effective than usual care. Research has shown that preferences are not always clear-cut and readily accessible; rather, they are inchoate and constructed with experience.^19^ This finding points to a potential opportunity to strengthen the current training curriculum by building clinicians’ skills to help patients explore, clarify, and strengthen preferences.

Second, the analyses suggested that physicians in the intervention group who spent at least 5 minutes on the topic of CRC screening had more productive conversations with patients than physicians in the comparator group who spent a similar amount of time. The SDM training provided practical scripts for physicians to use to introduce the topic, present options, elicit preferences, and support implementation. Of course, primary care office visits are short and older adults often have multiple health issues that need to be covered during a visit. In the initial PRIMED study, PCPs in the intervention group were more likely to talk about CRC screening than PCPs in the comparator group (72% vs 60% of visits; *P* = .03)^11^; an analysis of additional PRIMED study data found that any amount of time spent discussing CRC screening increased clinicians’ ability to accurately diagnose patient preferences.^20^ Although there are limited data on time required to have SDM conversations, Braddock et al^21^ found that visits that incorporated more elements of SDM took, on average, 2 minutes longer. Brief SDM discussions may be worth the extra few minutes if they lead to higher follow-through with the patient-preferred approach, thus decreasing the need for subsequent discussions.

Although colonoscopy is considered the standard of care for CRC screening at the participating PRIMED sites, another potential effect of the SDM training was to increase discussion and use of stool-based tests for older adults. The 12-month results of this study suggest that more patients seen by physicians in the intervention group completed screening. Furthermore, the additional screening was due to higher use of stool-based tests, because the rates of colonoscopy were virtually identical across groups. The primary study results showed that physicians in the intervention group were more likely to discuss and recommend stool-based testing, leading to increased use of stool-based tests.^11^ This result is consistent with the deimplementation literature, indicating greater patient acceptance of reducing or replacing screening rather than removing or stopping an intervention.^8^ It is important to ensure that older patients are aware that stool-based testing may be an option to continue monitoring.

### Limitations

This study had several limitations. The cluster randomization resulted in a slight imbalance between groups, with the intervention group having higher percentages of women and of patients with prior testing than the comparator group. All analyses adjusted for these differences. As a secondary outcome, the study was powered to detect a large difference (12.5%) in concordance rates and was likely underpowered to detect the small difference (4.7%) found here. The limited racial and ethnic diversity and the high education level of the patient population limit the generalizability of these results. Finally, the COVID-19 pandemic caused substantial disruptions to clinical care, including the rescheduling of primary care visits, the use of a new telemedicine format, and limited access to colonoscopy. Although the randomization should have ensured that COVID-19 affected groups equally, patients’ ability to schedule a colonoscopy despite clear preference and strong intentions may have been negatively affected due to the pandemic. Some of these issues were largely out of physicians’ control and not readily addressable via SDM training; thus, they may have biased the results of concordance comparison between groups to null. The study did not capture test orders (only completed test results), and it is possible that calculating concordance using orders would have shown more attention to patient preferences.

## Conclusions

In this secondary analysis of a cluster randomized clinical trial, we found marked diversity in older patients’ preferences for CRC testing, which supports an SDM process rather than rigid age-based cutoffs for screening. With an aging population, having good conversations about whether or when to stop cancer screening is increasingly important. Furthermore, it appears that reducing the intensity of testing (eg, pursuing stool-based tests or stopping testing altogether) and discussing alternatives to colonoscopy, even for patients with prior experience with colonoscopy, may be acceptable to physicians and patients alike. Although the SDM training intervention did not make a statistically significant improvement in concordance in this sample, future work to refine and evaluate clinical decision support (in the form of an electronic advisory or reminder), as well as focused SDM skills training for PCPs, may promote high-quality, preference-concordant decisions about CRC testing for older adults.

## Notes

Supplement 1.
Trial Protocol

Supplement 2. **eTable 1.** Shared Decision-Making Training Course Content and Sample Reminder Notification **eTable 2.** Respondents vs Nonrespondents (Including Those Who Opted Out Prior to the Visit) **eTable 3.** Physician Characteristics for the Study Groups

Supplement 3.
Data Sharing Statement
