# Supplementary material for: Shared Decision-Making in Colorectal Cancer Screening for Older Adults: A Secondary Analysis of a Cluster Randomized Clinical Trial
Source: JAMA Netw Open. 2024 Aug 23;7(8):e2429645. doi: 10.1001/jamanetworkopen.2024.29645 (PMC11344228; doi:10.1001/jamanetworkopen.2024.29645)
Supplement: Supplement 1. — Trial Protocol [file jamanetwopen-e2429645-s001.pdf]

Title: PROMOTING INFORMED DECISIONS ABOUT CANCER SCREENING IN OLDER ADULTS (PRIMED STUDY)

Karen Sepucha (PI)

The document includes the

1. original approved IRB protocol summary (dated 01.04.2019),
2. summary of amendments to the protocol,
3. current approved protocol summary (dated 03.29.21)
4. original research proposal – included because the Mass General Brigham IRB did not require a statistical analysis plan as part of the protocol summary for minimal risk trials

PARTNERS HUMAN RESEARCH COMMITTEE
PROTOCOL SUMMARY

Answer all questions accurately and completely in order to provide the PHRC with the relevant information to assess the risk-benefit ratio for the study. Do not leave sections blank.

PRINCIPAL/OVERALL INVESTIGATOR

Karen Sepucha, PhD

PROTOCOL TITLE

Promoting Informed Decisions about Cancer Screening in Older Adults (PRIMED Study)

FUNDING

Patient-Centered Outcomes Research Institute

VERSION DATE

January 4, 2019

SPECIFIC AIMS

Concisely state the objectives of the study and the hypothesis being tested.

As people age, medical decisions become more complex, including conversations about cancer screening. For patients aged 76-85, the United States Preventive Services Task Force (USPSTF) advises clinicians that decisions about colorectal cancer (CRC) screening should be individualized based on overall health and prior screening history (C recommendation).[i] However, studies find that many older adults are not well-informed about, nor meaningfully engaged in, decisions about whether to continue CRC screening. Shared decision making (SDM) has been shown to improve the quality of decisions about initiating cancer screening but little is known about its effectiveness for decisions about stopping interventions. This proposal addresses an important gap in our understanding of how to support clinicians and older patients in making good decisions about whether to continue CRC screening or not.

We will conduct a comparative effectiveness trial that will randomly assign clinicians at participating academic and community practices to one of two different decision support strategies. The first strategy (Registry arm) takes a population health management (PHM) approach and uses a patient registry to identify and track use of CRC screening among older adults for each clinician. The second strategy will enhance the registry by adding an established, multi-faceted SDM training program for clinicians (SDM Skills arm). We will enroll patients of participating primary care providers (PCPs), aged 76-85, who are due or overdue for CRC screening, and survey them shortly after an office visit to determine the impact of the two strategies on outcomes of importance to patients. We plan to randomly assign about 60 participating PCPs to the SDM skills or Registry arms, and enroll about 500 of their eligible patients. We will compare reports of shared decision making, patients' knowledge, and rates of patients who get their preferred option for CRC screening across study arms. We will also compare CRC screening rates across arms and to concurrent and historical controls. Through this project, we will accomplish the following specific aims:
Aim 1: Determine the impact of the approaches on patients' involvement in decision making and knowledge about the risks and benefits of continued CRC screening.

Hypothesis 1.1 (Primary outcome): Patients seen by clinicians in the SDM Skills arm will report more SDM discussions about cancer screening compared to the Registry arm.

Hypothesis 2.1 (Secondary outcome): Patients seen by clinicians in the SDM Skills arm will have higher knowledge of the benefits and harms of CRC screening and treatment compared to Registry arm.

Aim 2: Examine the effects of the interventions on patients' preferences for screening, the extent to which patients receive their preferred approach to screening, and on CRC screening rates.

Hypothesis 2.1 (Secondary outcome): A higher percentage of patients will receive their preferred approach to screening in SDM Skills arm compared to the Registry arm.

Hypothesis 2.2: (Secondary outcome) Both interventions will reduce CRC screening rates compared to concurrent controls (rates of clinicians who are not involved in study).

Aim 3: Examine the impact of the interventions on physicians' confidence with and skills for SDM in this setting.

Hypothesis 3.1: Clinicians in the SDM Skills arm will have higher confidence in their SDM skills.

Hypothesis 3.2: Clinicians in the SDM Skills arm will demonstrate more SDM skills in simulated patient interactions than the Registry arm.

The study will advance our understanding of how to best communicate evidence of cancer screening benefits and harms to older adults. Better decisions about whether or when to stop screening may reduce unnecessary tests and treatments and allow patients to avoid potential harms of screening.

BACKGROUND AND SIGNIFICANCE

Provide a brief paragraph summarizing prior experience important for understanding the proposed study and procedures.

Colorectal cancer (CRC) is a common, lethal disease that affects both men and women. In 2016, an estimated 135,000 people were diagnosed with CRC and 49,000 people died of it. The incidence of CRC increases with age, and the average age of diagnosis is 68 for men and 72 for women. CRC screening is widespread, and data indicate that 65% of adults over 65 were up-to-date with CRC screening tests; however, almost one quarter of adults 75 and older have never been screened for CRC. The screening is performed using a variety of methods, including stool-based testing requiring patients to collect stool specimens at home, and direct visualization testing such as colonoscopy. If stool-based testing is positive, then additional testing with a colonoscopy is recommended. There is evidence from observational and randomized trials that all methods for CRC screening are effective at reducing mortality attributed to colorectal cancer, provided the tests are conducted at recommended intervals with follow-up as needed.

Although CRC screening is recommended for adults aged 50-75, the USPSTF advises clinicians to make an individual decision for adults aged 76-85. Older adults often have a small potential benefit from screening and are at higher risk for complications, particularly complications of colonoscopy. The choice of whether to continue or stop screening depends significantly on patients' individual risk of colorectal cancer, their overall health, as well as their preferences for testing. Those who are able to undergo treatment if cancer is found and those who are otherwise healthy with long life expectancy may be more inclined to continue. Further, the USPSTF notes that adults aged 76-85 who have never been screened are more likely to benefit from CRC screening than those with prior testing.

There is a growing need to address appropriate use of cancer screening tests in older adults. Screening for asymptomatic disease comes with costs and potential harms. Shared decision making (SDM) is an established approach to engaging and informing patients in medical decisions. Currently, there is a lack of evidence on effective interventions to support clinicians in communicating with patients 76-85 about the benefits and harms of cancer screening and

tailoring decisions to what matters most to patients. This study will compare two established interventions to advance our understanding of how to support clinicians in conducting SDM conversations with older adults who may be considering stopping cancer screening.

RESEARCH DESIGN AND METHODS

Briefly describe study design and anticipated enrollment, i.e., number of subjects to be enrolled by researchers study-wide and by Partners researchers. Provide a brief summary of the eligibility criteria (for example, age range, gender, medical condition). Include any local site restrictions, for example, "Enrollment at Partners will be limited to adults although the sponsor's protocol is open to both children and adults."

The main study is a cluster randomized trial enrolling about 60 primary care clinicians across all sites. We estimate including about 30-35 clinicians from primary care practices affiliated with Massachusetts General Hospital (MGH) and Brigham and Women's Hospital (BWH), 10-15 from Maine Medical Center (MMC), and 10-15 from community practices affiliated with Newton Wellesley Hospital (NWH) and North Shore Medical Center (NSMC). The participating clinicians at each site will be placed into two groups stratified by gender and years of experience. Then, each group will be randomly assigned to one of two arms: Registry arm or Registry plus SDM Skills training arm (SDM arm). Clinicians in Registry arm will be notified about their patients aged 76-85 who are due for colorectal cancer screening with an upcoming visit. Clinician participants in the SDM arm will also receive the Registry notification and in addition, they will participate in a SDM skills course that includes online training and telephone-based simulated patient interactions.

After the training, study staff will track and enroll eligible patients who have upcoming visits with participating clinicians in both arms. We plan to enroll about 10 patients per clinician for a total of 500 patients. The clinicians will complete a short survey after each eligible patient visit. The patients will be invited to participate in the survey study and will be asked to complete a survey after their visit and another short survey one year later. Patients will be asked whether a spouse, friend or caregiver was involved in the decision-making process and if so, whether they would be willing to invite them to participate in the study. Study staff will follow up to enroll caregivers that patient participants identify and administer a short survey. We expect that about half of the patients will identify a caregiver, so about 250 caregivers will be surveyed.

Staff will track colorectal cancer screening for 12 months after the recorded date of visit for participating patients, for participating clinicians and for non-participating clinicians across these sites to examine trends in screening over time. Historical controls will also be collected to calculate rates of screening across the sites for the 2 years prior to the study (approximately calendar years 2017 and 2018).

The eligibility criteria for the clinician and patient participants are in Tables 1 and 2.

Table 1: Eligibility for clinician participants

Eligible	Ineligible
• Primary Care Physician (MD or NP)<br>• Have ≥30 potentially eligible patients in their panel<br>• Use of Epic electronic health record	• Residents, medical students

Table 2: Eligibility for patient participants

Eligible	Ineligible
• Adults, age 76-85 at the time of a scheduled visit<br>• Scheduled for non-urgent office visit with a participating clinician during the study period<br>• Due or overdue for colorectal cancer screening (e.g. never been screened, <1 year to follow-up interval indicated on last test).	• Prior diagnosis of colon or rectal cancer, inflammatory bowel disease or genetic disorder that raises CRC risk (hereditary non-polyposis CRC and familial adenomatous polyposis)<br>• Unable to consent for themselves (moderate to severe dementia or other major cognitive limitations)<br>• Unable to read or write in English or Spanish

Briefly describe study procedures. Include any local site restrictions, for example, "Subjects enrolled at Partners will not participate in the pharmacokinetic portion of the study." Describe study endpoints.

Clinicians will be recruited from primary care practices, both internal medicine and family medicine, affiliated with MGH, BWH, MMC, NWH and NSMC. The investigators will work with Steve Atlas who has an IRB approved protocol (2004P002796) to help identify the number of eligible patients by clinician and will then target those clinicians with high number of eligible patients for recruitment on the trial. IRB protocol 2004P002796 remains an active study and continues to operate per an IRB approved protocol and amendments. The study uses data from patients followed in MGH affiliated primary care practices for research and quality improvement purposes. The IRB protocol specifically pertains to research aspects that go beyond data used for administrative purposes as part of usual hospital operations. The study involves implementing a previously validated and published methodology to identify and link patients seen in MGH primary care practices to specific providers. Information is also collected on patient characteristics and outcomes of care. The data for this study is updated on a yearly basis. The information collected as part of this study can be made available to other IRB approved studies such as the current PRIMED submission if permitted in the IRB submission. Data collected as part of IRB protocol 2004P002796 will be used to identify MGH primary care providers with patients who meet eligibility criteria for the PRIMED submission. Though IRB protocol 2004P002796 will help the current submission with identifying potential primary care providers and patients, all aspects of contacting these providers and patients are covered in the current submission.

The participating clinicians at each site will be placed into two groups stratified by gender, years in practice, and number of clinic sessions per week. Then, each group will be randomly assigned to one of two arms: Registry arm or Registry plus SDM Skills training arm (SDM arm).

- Clinicians in Registry arm will complete a baseline telephone-based simulated patient interaction to evaluate their SDM skills. Once patient enrollment begins study staff will send periodic notification of their patients aged 76-85 who are due for colorectal cancer screening with an upcoming visit.
- Clinician participants in the SDM arm will complete an online SDM skills course, two telephone-based simulated patient interactions, and monthly facilitated case-based discussions. Once patient enrollment begins, study staff will send periodic notification of their patients aged 76-85 who are due for colorectal cancer screening with an upcoming visit.

Study staff will use existing functionality in the electronic medical record (EMR) to generate a list of patients 75 and older with CRC screening status, prior CRC screening results, and upcoming

visit dates for each participating clinician. Staff will also work within the EMR at each site to develop an automated report of these items as available.

Our target is to enroll approximately 10 patients per clinician for a total of 500 patients. The clinicians will complete a short survey after each eligible patient visit. The patients will be invited to participate in the survey study and will be asked to complete a survey after their visit and another short survey one year later. Patients will be asked whether a spouse, friend or caregiver was involved in the decision-making process and if so, whether they would be willing to invite them to participate in the study. Study staff will follow up to enroll caregivers that patient participants identify and administer a short survey. We expect that about half of the patients will identify a caregiver, so about 250 caregivers will be surveyed.

The research coordinator will track the number of study participation invitations sent to each clinician as well as the number of clinicians indicating interest. We will track any reason given for refusal to join the study as well as any reason for dropping out of the study after randomization for reporting in CONSORT flow diagram. Staff will track completion of each activity (baseline survey, simulated patient interaction, training course, etc) for each clinician participant.

Staff will also track all patient participants screened, reason for ineligibility, the number sent invitation by mail, the number who opted out or otherwise declined participation, those lost to follow-up for any reason, and any reasons given for the refusal to participate for reporting in CONSORT flow diagram. There are no formal written consent procedures in this study. The research presents no more than minimal risk of harm to subjects and involves no procedures for which written consent is normally required. Consent for the study will be implied by completion of the one survey for patient participants and email consent for clinician participants.

The primary outcome for this study is whether patients report more SDM in the visit with participating clinicians about CRC screening in the SDM arm compared to the Registry arm. Key secondary outcomes will be whether patients are knowledgeable about CRC screening benefits and harms, and whether clinicians understand patients' preferences and tailor screening decisions appropriately.

Patient reported measures: patients will complete a survey shortly after the visit and again about one year later.

- **Shared Decision Making Process (SDMP) Survey:** Four items assess the amount of shared decision making that occurs during a visit. These items are summed to generate a total score (0-4), with higher scores indicating greater patient involvement in decision making. The survey has been validated through its use in many studies, including two national studies of shared decision making for cancer screening and has strong evidence of acceptability, feasibility, reliability and validity.[Error! Bookmark not defined.][ii,iii] The survey has also been endorsed by National Quality Forum as a SDM performance measure (#2962).
- **Knowledge:** Five multiple choice knowledge items will assess patients' understanding of colorectal cancer screening adapted from the Colorectal Cancer Screening Decision Quality Instrument.[iv,v] A total knowledge score (0-5) will be calculated from the number of correct answers.
- **Risk perceptions:** One item will assess affective risk perception, or cancer worry. This item will be adapted from the National Cancer Institute's Health Information National Trends Survey (HINTS).[vi]
- **Patient's preferred approach to screening:** One item will assess patients' preferred approach to screening (with responses of colonoscopy, stool card test, no screening, not sure).

- **Overall health:** SF-1 will be used to assess patient's perception of overall health (poor to excellent)[vii]
- **Screening Recommendation and Time Spent:** One item will assess the patient's perception about their clinician's recommendation about CRC screening and one item will assess how much time was spent discussing CRC screening in the visit.
- **Single Item Literacy Screener**: One item that measures comfort with reading materials from health care providers. It has high specificity according to other, more detailed health literacy screening tools, and is able to be self administered.[viii,ix]
- **Demographics and CRC risk factors**: items will assess factors such as education, employment, marital status, family history of CRC, and alcohol use.
- **Barriers and facilitators to screening**: in the one year survey we will assess decision regret, and will ask patients who did not follow through with planned screening to discuss any barriers or reasons why the decision was not completed. For patients who had screening despite indicating desire to stop we will explore reasons for this change.

Study staff will supplement patient reported data with data collected via chart review. First, staff will review chart to confirm eligibility (e.g. age, dates and types of prior CRC screening tests as well as follow up test timing recommendation, CRC cancer history, dementia or cognitive impairment that would prevent participation, upcoming visit dates with participating clinician). Second, staff will access chart of participating patients to document CRC risk factors (e.g. family history, BMI, inflammatory bowel disease, diabetes, smoking status), abstract screening discussion in the visit note, subsequent CRC tests or procedures, complications related to CRC testing for participants, and findings of tests. Missing patient demographic information may also be supplemented via chart review.

Clinician, Practice and Network level CRC screening rates: We will use established, validated algorithms for calculating cancer screening rates using a combination of administrative, billing and clinical data. Dr. Atlas (co-I) led the algorithm development efforts at MGH and Partners, and he will work with the MMC team to ensure CRC screening rates are comparable across sites. Data is aggregated at the physician, practice and network (e.g. MGH, MMC, PCPO) level to identify the percentage of eligible patients up to date for screening during the historical control period, and concurrent observation period for study and non-study clinicians. Limited data on patient characteristics (e.g. age, gender, education, insurance status, etc) will be collected along with screening rates.

Clinician reported measures: clinicians will complete a baseline survey and a telephone-based simulated patient interaction (SPI) before staff start enrolling their patients onto the trial. They will complete a short survey after each eligible patient visit. After patient enrollment is complete, all clinicians will participate in a debrief interview.

- **Baseline clinician survey** will include the same CRC knowledge items as the patients and 3 items to assess clinicians' confidence in their ability to present benefits and harms, to discuss probabilities of benefits and harms and to elicit patients' goals and concerns during an office visit, each based on a five-point scale (not at all, a little, somewhat, very, and extremely confident).
- **Post visit survey:** 4 items SDMP survey will be adapted for use by clinicians, time spent discussing CRC screening in visit, recommendation, patient's preferred approach, and satisfaction with visit.
- **Baseline SDM skills assessment:** The telephone-based SPI will be transcribed and coded by two trained coders according to the well-validated Braddock's Informed Decision Making framework. The Braddock framework covers the core areas of SDM skills. [x,xi] A total score 0-9 will be calculated with higher scores indicating more SDM skills.

- **Debrief interview**: After patient recruitment is complete, study staff will conduct a brief interview with all participating clinicians and will follow a structured interview guide to assess clinicians' attitudes toward SDM, their perceptions of the study and satisfaction with the intervention, and ideas for improvement.

Caregiver measures: a short survey post visit will contain SDMP survey, their perception of physician recommendation, their preferred approach to screening and their perception of the patient's preference for screening.

Adherence to intervention(s): The online training platform will track completion of modules and time spent on the webinar. Staff will track completion rates and time for the SPIs, delivery of the registry reports, participation in monthly case discussion sessions, and documentation in notes of CRC screening discussions in order to examine whether outcomes are affected by adherence to the protocol.
Patients will self-report time spent discussing CRC screening in the visit. Finally, staff will conduct a short follow-up survey with patients at 12 months to confirm screening choice and reasons for any discrepancy between preferred and implemented approach (e.g. transportation, insurance, clinician recommendation, spouse/caregiver preference, other new or worsening illness).

All study staff are Collaborative Institutional Training Initiative (CITI) certified and will receive training from the PI and program manager in the study protocol. We will hold regular meetings to review screening, enrollment and completion data, to discuss protocol and standard operating procedures, and to identify and mitigate any issues that arise.

For studies involving treatment or diagnosis, provide information about standard of care at Partners (e.g., BWH, MGH) and indicate how the study procedures differ from standard care. Provide information on available alternative treatments, procedures, or methods of diagnosis.

The standard of care is that physicians may discuss appropriate screening options, including their benefits and risks with each patient and individualize a decision based on the patients' risk, overall health and preferences. In this project, we will proactively remind clinicians to have these conversations with eligible patients about continuing colorectal cancer screening. Whether the physician and patient have this conversation remains at their discretion during a clinic visit. No tests or treatments will be administered as part of this study.

Describe how risks to subjects are minimized, for example, by using procedures which are consistent with sound research design and which do not unnecessarily expose subjects to risk or by using procedures already being performed on the subject for diagnostic or treatment purposes.

There are minimal risks to participating individuals associated with or attributable to this study. The main risks are associated with loss of privacy of their health information. To minimize risks, all electronic data files that include clinician, caregiver and patient identifiers will be kept in a Partners protected server and only members of the research team will have access to the files. Files with PHI will only be accessed from Partners computers or encrypted laptops that are protected with SafeBoot. All patient information on eligibility screeners, chart reviews, and surveys collected at MMC will be sent securely using a secure file transfer to the Partners network. To ensure confidentiality, all paper surveys will be identified by study code number only and kept in a locked file cabinet and the scanned surveys and electronic files will be on password protected Partners server. Study papers (screeners, notes, surveys) that have been

scanned or entered into a database will be disposed of in the confidential shredder. To address issues of psychological discomfort, research assistants will inform patients that they may refuse to answer any question and may withdraw from the study at any time. To address privacy and confidentiality issues, analytic database with outcomes data will not contain any identifying information and will be coded by unique study ID number only.

Patients will be invited to complete survey questionnaires. The time required for patient participants to complete each survey is about 15 minutes. Participants may opt out of the survey study, may refuse to answer any question (or set of questions) and may discontinue their participation at any time. It will also be emphasized that whether or not subjects participate will not impact the medical care that they receive. The caregiver's survey should take less than 10 minutes to complete.

The clinicians in the Registry arm will spend about 1.5 hours on study related surveys and activities and clinicians in the SDM arm will spend about 3.5 hours on study related activities over the course of 12-18 months. This includes the baseline questionnaire (10 minutes), the simulated patient interaction (20 minutes), and the training course for those assigned to SDM arm (about 2 hours). Clinicians will also complete about 10 surveys after patient visits that should take 1-2 minutes to complete. The exit interview will be about 20 minutes.

Describe explicitly the methods for ensuring the safety of subjects. Provide objective criteria for removing a subject from the study, for example, objective criteria for worsening disease/lack of improvement and/or unacceptable adverse events. The inclusion of objective drop criteria is especially important in studies designed with placebo control groups.

Although there are no written informed consent forms, Drs. Simmons and Sepucha are responsible for assuring that clinician and patient participants are adequately informed prior to engaging in any research procedures, that all subjects meet eligibility criteria, and that the study is conducted according to the IRB-approved research plan. The patient population in this study is older, and may have significant co-morbidities which may limit life expectancy. Staff will confirm status of patients, particularly before contacting patient participants for the follow-up survey.

There are no formal stopping rules for this minimal risk study.

FORESEEABLE RISKS AND DISCOMFORTS

Provide a brief description of any foreseeable risks and discomforts to subjects. Include those related to drugs/devices/procedures being studied and/or administered/performed solely for research purposes. In addition, include psychosocial risks, and risks related to privacy and confidentiality. When applicable, describe risks to a developing fetus or nursing infant.

There are minimal risks to individuals participating in this project. The main risks are the time and effort involved in participating and the potential loss of privacy. All electronic data files that include clinician, caregiver and patient identifiers will be kept in a Partners protected servers and only members of the research team will have access to the files. Files with PHI will only be accessed from Partners computers or encrypted laptops that are protected with SafeBoot. All patient information on eligibility screeners, chart reviews, and surveys collected at MMC will be sent securely using a secure file transfer to the Partners network. To ensure confidentiality, all paper surveys will be identified by study code number only and kept in a locked file cabinet and

the scanned surveys and electronic files will be on password protected Partners server. Study papers (screeners, notes, surveys) that have been scanned or entered into a database will be disposed of in the confidential shredder. To address issues of psychological discomfort, research assistants will inform patients that they may refuse to answer any question and may withdraw from the study at any time. To address privacy and confidentiality issues, analytic database with outcomes data will not contain any identifying information and will be coded by unique study ID number only.

EXPECTED BENEFITS

Describe both the expected benefits to individual subjects participating in the research and the importance of the knowledge that may reasonably be expected to result from the study. Provide a brief, realistic summary of potential benefits to subjects, for example, "It is hoped that the treatment will result in a partial reduction in tumor size in at least 25% of the enrolled subjects." Indicate how the results of the study will benefit future patients with the disease/condition being studied and/or society, e.g., through increased knowledge of human physiology or behavior, improved safety, or technological advances.

All participating physicians will be notified of upcoming visits with eligible patients. SDM in the clinic visit has been shown to increase patient knowledge, reduce decisional conflict and improve the match between patients' preferences and their treatment choices. Those clinicians randomized to the SDM Skills arm may further benefit as prior work has shown the training results in increased confidence and competence in conducting SDM conversations with patients. Clinicians in both arms may benefit from the registry report as that may prompt them to discuss cancer screening with their older population of patients.

There are no direct benefits to patients from completing the surveys. The potential benefit to society is that the study will help determine the most effective approach to engaging and informing older patients about cancer screening.

As efforts to integrate SDM into routine care expand, understanding the effectiveness of interventions to achieve SDM is critical. This study will provide important new information on comparative effectiveness of different decision support strategies promoting SDM.

EQUITABLE SELECTION OF SUBJECTS

The risks and benefits of the research must be fairly distributed among the populations that stand to benefit from it. No group of persons, for example, men, women, pregnant women, children, and minorities, should be categorically excluded from the research without a good scientific or ethical reason to do so. Please provide the basis for concluding that the study population is representative of the population that stands to potentially benefit from this research.

The patient recruitment is limited to older men and women 76 to 85 years of age as the clinical guidelines on colorectal cancer screening highlight this age group as requiring an individual decision and thus are appropriate to engage in shared decision making. Children, younger adults, and pregnant women are not eligible for this decision. We will be targeting clinicians across the sites who care for a diverse patient population in order to increase enrollment of minority patients on the trial.

The clinician recruitment is focused on primary care clinicians (MD and NPs) who spend a significant portion of time seeing patients. Residents and other health care professional (e.g. RNs, social workers) are not eligible as they rarely consult patients regarding this decision.

When people who do not speak English are excluded from participation in the research, provide the scientific rationale for doing so. Individuals who do not speak English should not be denied participation in research simply because it is inconvenient to translate the consent form in different languages and to have an interpreter present.

Patient survey materials will be available in English, Spanish. We will try to include other languages based on the need of the population. Most patients seen at these sites speak either English or Spanish (>97%). Patients with other primary languages not translated for this study will be excluded from the survey portion of the study.

For guidance, refer to the following Partners policy:
Obtaining and Documenting Informed Consent of Subjects who do not Speak English
https://partnershealthcare-public.sharepoint.com/ClinicalResearch/Non-English_Speaking_Subjects.1.10.pdf

RECRUITMENT PROCEDURES

Explain in detail the specific methodology that will be used to recruit subjects. Specifically address how, when, where and by whom subjects will be identified and approached about participation. Include any specific recruitment methods used to enhance recruitment of women and minorities.

Clinicians will be recruited from primary care, internal medicine, and family medicine practices affiliated with MGH, BWH, MMC, NWH and NSMC. At MGH, there are about 190 adult primary care clinicians across 19 affiliated primary care practices, including 12 community-based practices and 3 hospital-based practices serving a diverse patient population in Eastern Massachusetts. Four of the practices are community health centers located in low-income urban communities around Boston and we plan to target clinicians at these centers to increase patient diversity. At MMC, there are 108 primary care providers at 10 affiliated primary care practices that will be screened for eligibility. Newton Wellesley Hospital and North Shore Medical Center have about 128 clinicians across 16 community practices. Current CRC screening rates for patients 76-85 range from 52%-65% at these sites. All participating sites use the Epic EMR with similar cancer screening registry functionality.

Clinician recruitment:

Investigators at each site will identify clinicians with a high volume of patients aged 76-85 and those who see patients at community health centers or centers with higher patient diversity. The recruitment and study procedure for clinicians is as follows:

1. The PIs and co-investigators will inform clinicians about the study and invite their participation in multiple ways: through presentations at clinical leadership meetings, practice meetings, through HDSC newsletter, through Partners Center for Population Health Newsletter, and through calls, meetings and emails to individual clinicians.
2. Clinicians will indicate interest in participating in the study by contacting the PIs or study staff (either via email, phone or in person).

3. Study staff will screen interested clinicians to collect information to confirm eligibility and will send an information sheet to clinicians that details the requirements of the study.
4. Clinicians will indicate their consent to participate by sending an email with their intention to join the study.
5. Clinicians will be randomly assigned to an arm by study statistician.
6. Clinicians assigned to the registry arm will have 4 weeks to complete:
    a. Baseline survey via RedCap or phone
    b. A telephone-based SPI conducted with standardized patients that will be audiotaped
    c. Short meeting with staff to review protocol and preferences for receiving the registry information about potentially eligible patients (e.g. 24h in advance of visit, weekly report, at morning huddle through medical asst)
    d. Study staff will send up to six reminder emails for each activity and make two reminder calls to encourage completion of each activity.
7. Clinicians assigned to the SDM training arm will have 4 weeks to complete:
    a. Baseline survey via RedCap or phone
    b. The online training course
    c. A telephone-based interaction conducted with a standardized patient. Study staff will email feedback 1 week after completion.
    d. Short meeting with staff to review protocol and preferences for receiving the information about potentially eligible patients (e.g. 24h in advance of visit, weekly report, at morning huddle through medical asst)
    e. Study staff will send up to six reminder emails for each activity and make two reminder calls to encourage completion of each activity.
8. Clinicians in the SDM Training arm will complete a second SPI about 8-12 weeks after the first and will have the opportunity to participate in monthly 'office hours' sessions—conference calls open to all participants in this arm that will be facilitated by SDM experts, primary care physicians and/or a gastroenterologist, to discuss cases and field questions and challenges that come up as they put the skills into practice.
9. After each eligible patient visit, staff will email a short questionnaire to participating clinicians. Staff will follow up with two reminder emails at 24 and 48 hours to complete the questions.
10. After patient enrollment is complete, staff will schedule an exit interview with clinicians. For participants who are not able to attend in person, the interview will be conducted by phone.

<u>Patient and caregiver recruitment:</u>

1. Study staff will review clinic schedules and medical records to identify eligible patients for participating clinicians prior to their scheduled visit.
2. About two weeks before the visit, the research coordinator will send a cover letter signed by the participating clinician and an information sheet describing the study to all eligible patients. The cover letter will have information for participants who wish to opt out of the survey.
3. 1-3 days before the visit, staff will call all eligible patients who did not opt out to discuss the study and answer any questions. Staff will inquire about subject's preference to receive survey via email or mail. If email is preferred, staff will discuss privacy and obtain permission to send the survey via email without send secure (and confirm address). Study staff will read the following statement to patients, "The Partners HealthCare standard is to send email securely. This requires you to initially set up and activate an account with a password. You can then use the password to access secure emails sent to you from Partners HealthCare. If you prefer, we can send you "unencrypted" email that is not secure and could result in the unauthorized use or disclosure of your

information. If you want to receive communications by unencrypted email despite these risks, Partners HealthCare will not be held responsible. Your preference to receive unencrypted email will apply to emails sent from this research study only. If you wish to communicate with other research staff at Partners regarding additional studies, your preference will have to be documented with each research group." After reading the required warning language, study staff will ask for the patient's verbal agreement. The agreement and agreement date will be noted in the research records. Finally, staff will determine whether a caregiver will be involved at the visit and if so, staff will obtain contact information for the caregiver.

4. After the visit, staff will send the patient a survey packet. The mailed survey packets will include a $5 incentive. Patients completing the survey via RedCap will receive a letter with a reminder to check their email, a copy of the link, and a $5 incentive.
5. Patient consent for the study will be implied by return of the completed survey.
6. Staff will also send a survey packet to the caregiver if the patient identified one.
7. Staff will make up to three reminder phone calls (or emails for patients who preferred email), send one reminder paper or email mailing, and then make up to 3 additional reminder calls to non-responders. Patients will be given the option to complete the survey by phone.
8. Approximately one year after initial visit with PCP, a research coordinator will confirm status of each patient and will document any colorectal cancer screening tests and procedures completed since the visit from the medical record.
9. Staff will send the second survey with a $5 incentive to all patients who are still alive in same mode as initial survey (email or mail).
10. Study staff will follow-up a similar protocol as with the initial survey by making up to three phone reminders (or email reminders), followed by a mailed reminder packet, and up to three phone reminders for all non responders. Patients will be given the option to complete the survey by phone.
11. All participants who complete a survey will receive a thank you note.

All study staff are CITI certified and will receive training from the PI and program manager in the study protocol. We will hold regular meetings to review screening, enrollment and completion data.

Provide details of remuneration, when applicable. Even when subjects may derive medical benefit from participation, it is often the case that extra hospital visits, meals at the hospital, parking fees or other inconveniences will result in additional out-of-pocket expenses related to study participation. Investigators may wish to consider providing reimbursement for such expenses when funding is available

- At MGH, BWH, NWH and NSMC:
  - Clinicians in SDM skills arm will receive a total of $100 and 2-3 hours of risk management CME credit, as well as MOC Part IV credit.
  - Clinicians' name will be entered into a quarterly lottery for a $50 amazon gift card each time they complete a survey on one of their patient participants.
- At MMC, the clinicians in the SDM arm who complete the course will receive risk management CME credit, as well as MOC Part IV credit, and the practice will receive a comparable stipend to distribute as per their policy.
- Patients and caregivers will receive a $5 incentive with each survey.

For guidance, refer to the following Partners policies:

Recruitment of Research Subjects
https://partnershealthcare-public.sharepoint.com/ClinicalResearch/Recruitment_Of_Research_Subjects.pdf

Guidelines for Advertisements for Recruiting Subjects
https://partnershealthcare-public.sharepoint.com/ClinicalResearch/Guidelines_For_Advertisements.1.11.pdf

Remuneration for Research Subjects
https://partnershealthcare-public.sharepoint.com/ClinicalResearch/Remuneration_for_Research_Subjects.pdf

CONSENT PROCEDURES

Explain in detail how, when, where, and by whom consent is obtained, and the timing of consent (i.e., how long subjects will be given to consider participation). For most studies involving more than minimal risk and all studies involving investigational drugs/devices, a licensed physician investigator must obtain informed consent. When subjects are to be enrolled from among the investigators' own patients, describe how the potential for coercion will be avoided.

There are no formal written consent procedures in this project for either patients, clinicians, or the non-intervention group. The research presents no more than minimal risk of harm to subjects and involves no procedures for which written consent is normally required. Clinician consent will be implied by email indicating interest and patient and caregiver consent for the study will be implied by completion of the first questionnaire.

Clinicians will be provided a written information sheet that describes the requirements of the study and will be instructed to send an email to the PIs or study staff indicating their interest in participating. By agreeing to be on the study, clinicians will give consent to study staff to contact their eligible patients.

Eligible patient participants will be given an information sheet that describes the risks and benefits of the study and a cover letter inviting them to participate in the survey. The invitation will include information about how to opt out of the survey portion of study. Patient subjects will be given 7 days to review the material and opt out by calling or emailing the study staff. Participants who do not opt out will be contacted by phone by research staff and can indicate their decision to accept or decline participation when contacted. Consent will be implied by the return of the completed survey.

The principal investigators' names and contact information will be available on the information sheet if participants have any questions or concerns about the study. The study staff will be available by phone or email to discuss the study and answer any questions. Each site has at least one primary care physician co-investigator who will be available by pager and study staff/PI will be available by phone to answer any questions.

Patients and caregivers will give verbal consent if they wish to receive surveys via unencrypted emails. The IRB information regarding send-secure vs. unencrypted emails will be included on the invitation and research staff will discuss this with the patients when they join the study. Patients and caregivers may also be read the IRB policy and ask for verbal consent to receive

unencrypted emails over the phone. Participants can choose to receive the surveys via a send-secure email or on paper in the mail if they do not wish to receive unencrypted emails.

Study materials will emphasize that whether or not patients participate will have no effect on the health care they receive.

NOTE: When subjects are unable to give consent due to age (minors) or impaired decision-making capacity, complete the forms for Research Involving Children as Subjects of Research and/or Research Involving Individuals with Impaired Decision-making Capacity, available on the New Submissions page on the PHRC website:
https://partnershealthcare.sharepoint.com/sites/phrmApply/aieipa/irb

For guidance, refer to the following Partners policy:
Informed Consent of Research Subjects:
https://partnershealthcare-public.sharepoint.com/ClinicalResearch/Informed_Consent_of_Research_Subjects.pdf

DATA AND SAFETY MONITORING

Describe the plan for monitoring the data to ensure the safety of subjects. The plan should include a brief description of (1) the safety and/or efficacy data that will be reviewed; (2) the planned frequency of review; and (3) who will be responsible for this review and for determining whether the research should be altered or stopped. Include a brief description of any stopping rules for the study, when appropriate. Depending upon the risk, size and complexity of the study, the investigator, an expert group, an independent Data and Safety Monitoring Board (DSMB) or others might be assigned primary responsibility for this monitoring activity.

NOTE: Regardless of data and safety monitoring plans by the sponsor or others, the principal investigator is ultimately responsible for protecting the rights, safety, and welfare of subjects under his/her care.

The data sources will be clinician surveys, transcripts of simulated patient interactions, patient surveys, caregiver surveys and, electronic health record information.

There are no foreseeable safety risks to participants for participating in a simulated patient interaction or completing a survey. Study staff will protect the privacy of research study participants as described in the Privacy and Confidentiality section. It is possible that participants may be upset by a question in the interview or survey, although our experience with similar questionnaires in other topics (including breast cancer decision making and decision making for joint replacement surgery) have found that it is rare for participants to be upset. Nevertheless, study staff will screen for adverse events and address them as described in the next section.

Study data will be accessible at all times for the Co-PIs to review. The project manager and co-PIs will examine study conduct including enrollment, accrual, drop-outs, and protocol deviations on a weekly or every other week basis with the staff at each site. Study staff will review study related data including comments from the SPIs, reminder phone calls to participants, participant surveys and will notify the PI about any serious or moderate potential adverse events (AEs)

immediately and any minor or potential ones at regular meetings. The Co-PIs will review AEs individually real-time and in aggregate on a regular basis at team meetings. No SAEs are expected based on the minimal risk trial. However, the Co-PIs and co-investigators will review potentially serious adverse events (SAEs), as soon as they are discovered. The Co-PIs will ensure all protocol deviations, AEs, and SAEs are reported to the IRB within required time frame based on severity, and will file an HRC AE Form within 10 working days as needed.

There are no formal stopping rules for this minimal risk study.

Describe the plan to be followed by the Principal Investigator/study staff for review of adverse events experienced by subjects under his/her care, and when applicable, for review of sponsor safety reports and DSMB reports. Describe the plan for reporting adverse events to the sponsor and the Partners' IRB and, when applicable, for submitting sponsor safety reports and DSMB reports to the Partners' IRBs. When the investigator is also the sponsor of the IND/IDE, include the plan for reporting of adverse events to the FDA and, when applicable, to investigators at other sites.<br><br>NOTE: In addition to the adverse event reporting requirements of the sponsor, the principal investigator must follow the Partners Human Research Committee guidelines for Adverse Event Reporting

No serious adverse events are expected. The name and contact information for the principal investigator will be included on study information sheet as well as contact for study staff and MGH IRB in case participants have a problem. We will have a clinical co-investigator for each topic who will be able to consult on any clinical issues that arise during the course of the interviews or surveys. However, if a serious adverse event occurs relating to the study, then the principal investigator will report the event to the IRB within 24 hours and will file an HRC Adverse Event Form within 10 working days. If a mild or moderate adverse event occurs, the principal investigator will summarize the event in the progress report at continuing review.

Study staff will be instructed to review surveys within 48 hours of receipt and to notify the PI about any potentially serious events immediately and all other events at regularly scheduled meetings. Study staff will keep records of any feedback, questions, concerns and/or complaints that are received and we will address them with the co-investigators and staff as needed.

MONITORING AND QUALITY ASSURANCE

Describe the plan to be followed by the principal investigator/study staff to monitor and assure the validity and integrity of the data and adherence to the IRB-approved protocol. Specify who will be responsible for monitoring, and the planned frequency of monitoring. For example, specify who will review the accuracy and completeness of case report form entries, source documents, and informed consent.<br><br>NOTE: Regardless of monitoring plans by the sponsor or others, the principal investigator is ultimately responsible for ensuring that the study is conducted at his/her investigative site in accordance with the IRB-approved protocol, and applicable regulations and requirements of the IRB.

The study staff and the principal investigator at each site will have routine meetings during the study period to ensure the project proceeds as intended per the protocol. All participant

screening and enrollment will be tracked on password protected servers using an Access or RedCap database. The information is stored behind a firewall and only study staff will have access to it as needed. We will track recruitment rates and response rates weekly and identify issues as they come up. The study staff will complete all required documents for the study binder and this will be reviewed quarterly by the project manager and one of the principal investigators.

Limited data will be kept on clinician non-responders for those who received an individual invitation (site, age, gender, patient volume and years in practice) as well as patient non-responders including age, gender, physician, and all elements in the eligibility screener. This information will be used to examine non-response bias.

For guidance, refer to the following Partners policies:

Data and Safety Monitoring Plans and Quality Assurance
https://partnershealthcare-public.sharepoint.com/ClinicalResearch/DSMP_in_Human_Subjects_Research.pdf

Reporting Unanticipated Problems (including Adverse Events)
https://partnershealthcare-public.sharepoint.com/ClinicalResearch/Reporting_Unanticipated_Problems_including_Adverse_Events.pdf

PRIVACY AND CONFIDENTIALITY

Describe methods used to protect the privacy of subjects and maintain confidentiality of data collected. This typically includes such practices as substituting codes for names and/or medical record numbers; removing face sheets or other identifiers from completed surveys/questionnaires; proper disposal of printed computer data; limited access to study data; use of password-protected computer databases; training for research staff on the importance of confidentiality of data, and storing research records in a secure location.

NOTE: Additional measures, such as obtaining a Certificate of Confidentiality, should be considered and are strongly encouraged when the research involves the collection of sensitive data, such as sexual, criminal or illegal behaviors.

Special efforts will be made to protect the privacy of subjects. We will review the subject's medial record to confirm eligibility to participate in the study. We will have names and addresses of eligible participants and this information will be kept separate from the study data (e.g. surveys and/or interview notes). All participants--patients, clinicians and caregivers--will receive a code number and the surveys and other data will only be identified by code number. A separate password-protected electronic file will contain the codes linked to identifying information. Only the MGH study staff and investigators will have access to this file. These will be kept as long as required by the research project. After the study has been completed the personal contact information of all eligible participants will be destroyed.

All files (e.g. eligibility screeners) that contain PHI will be kept in a locked file cabinet or in a secure offsite file storage location or on a password protected Partners shared drive.

Patient confidentiality will be maintained as is routine for all patient care privacy guidelines. All research staff are CITI certified and will be trained on the importance of data confidentiality.

SENDING SPECIMENS/DATA TO RESEARCH COLLABORATORS OUTSIDE PARTNERS

Specimens or data collected by Partners investigators will be sent to research collaborators outside Partners, indicate to whom specimens/data will be sent, what information will be sent, and whether the specimens/data will contain identifiers that could be used by the outside collaborators to link the specimens/data to individual subjects.

To promote research replicability, transparency and future use of the data, de-identified data sets will be created and will be available, by request, to outside researchers. The de-identified data sets will also be deposited in an open access service such as, ICPSR (https://www.icpsr.umich.edu/icpsrweb/). On ICPSR, individuals must register and agree to ICPSR's Responsible Use statement prior to accessing datasets. Additionally, before a dataset is made available for access, ICPSR completes a detailed review of all datasets to assess disclosure risk. If necessary, ICPSR modifies data to reduce disclosure risk or limits access to datasets for which modifying the data would substantially limit their utility or the risk of disclosure remains high. No information that contains identifiers or that could be used to link an individual to the data will be included in the de-identified data set. The information sheets will contain the following language: After the study is completed, all identifiable information will be removed from the data and after removal, the de-identified information will be deposited in an open access service to promote use of the data by other researchers.

Specifically address whether specimens/data will be stored at collaborating sites outside Partners for future use not described in the protocol. Include whether subjects can withdraw their specimens/data, and how they would do so. When appropriate, submit documentation of IRB approval from the recipient institution.

No identifiable data on Partners patients will be stored outside MGH. MMC patient data that is collected outside Partners will be received by the MGH research team (see details below).

MMC will have access to the clinician participant data across all sites, as needed, to schedule and conduct the simulated patient interactions. Any emails that contain identifiable clinician data will be sent using SendSecure, and any large files will be sent using secure file transfer. MMC will only have access to de-identified data sets for the Partners patients and caregivers.

RECEIVING SPECIMENS/DATA FROM RESEARCH COLLABORATORS OUTSIDE PARTNERS

When specimens or data collected by research collaborators outside Partners will be sent to Partners investigators, indicate from where the specimens/data will be obtained and whether the specimens/data will contain identifiers that could be used by Partners investigators to link the specimens/data to individual subjects. When appropriate, submit documentation of IRB approval and a copy of the IRB-approved consent form from the institution where the specimens/data were collected.

Eligibility screeners, patient, caregiver and clinician surveys, and medical record information will be collected from participants at MMC, by MMC-affiliated study staff. This patient health

information is necessary for assessing patient eligibility for participation and for administering the study protocol. As such, this patient information will be sent from MMC to MGH study staff via a secure file transfer or REDCap. The paper surveys collected at MMC will be scanned and sent to the MGH research team using a secure file transfer, and the paper copies will be transported for ultimate storage or confidential disposal at MGH.

All electronic files that contain patient identifiers will be kept Partners protected servers and will only be accessed with Partners computers or encrypted laptops.

Eligibility and medical chart review data will be collected via REDCap (Research Electronic Data Capture). REDCap is a free, secure, HIPAA compliant web-based application hosted by the Partners HealthCare Research Computing, Enterprise Research Infrastructure & Services (ERIS) group. The system offers easy data manipulation with audit trails, reports for monitoring and querying participant records, and an automated export mechanism to common statistical packages (SPSS, SAS, Stata, R/S-Plus).

---

[i] US Preventive Services Task Force. Screening for colorectal cancer US Preventive Services Task Force Recommendation Statement. The Journal of the American Medical Association 2016;315(23):2564-2575.
[ii] Hoffman T, Lewis CL, Pignone MP, et al. Decision-making processes for breast, colorectal, and prostate cancer screening: the DECISIONS survey. *Medical Decision Making*. 2010;30(5 Suppl):53S-64S.
[iii] Sepucha K, Stringfellow V, Fowler FJ. Shared Decision Making (SDM) Process Survey: Validity and Reliability of a Short, Patient-Reported Measure of SDM. 2017 Society for Medical Decision Making Annual Meeting abstract.
[iv] Sepucha K, Feibelmann S, Cosenza C, Levin CA, Pignone M. Development and evaluation of a new survey instrument to measure the quality of colorectal cancer screening decisions. *BMC medical informatics and decision making*. 2014;14(1):72.
[v] Hoffman RM, Elmore JG, Pignone MP, Gerstein BS, Levin CA, Fairfield KM. Knowledge and values for cancer screening decisions: Results from a national survey. Patient Educ Couns. 2016 Apr;99(4):624-30.
[vi] Health Information National Trends Survey. https://hints.cancer.gov/ Accessed December 5, 2016.
[vii] DeSalvo KB, Bloser N, Reynolds K, He J, Muntner P. Mortality Prediction with a Single General Self-Rated Health Question: A Meta-Analysis. *Journal of General Internal Medicine*. 2006;21(3):267-275. doi:10.1111/j.1525-1497.2005.00291.x.
[viii] Morris NS, MacLean, CD, Chew LD, Littenberg B. The Single Item Literacy Screener: evaluation of a brief instrument to identify limited reading ability. *BMC Family Practice*. 2006;7(1):1.
[ix] Brice JH, Foster MB, Principe S, Moss C, Shofer FS, Falk RJ, DeWalt DA. Single-item or two-item literacy screener to predict the S-TOFHLA among adult hemodialysis patients. *Patient education and counseling*. 2014;94(1):71-75.
[x] Leader A, Daskalakis C, Braddock C, Kunkel EJ, et al. Measuring Informed Decision Making about Prostate Cancer Screening in Primary Care. Med Dec Making. 2011; 32(2):327-36.
[xi] Price EL, Bereknyei S, Kuby A, Levinson W, Braddock CH. New elements for informed decision making: a qualitative study of older adults' views. Patient education and counseling. 2012;86(3):335-341.

PROMOTING INFORMED DECISIONS ABOUT CANCER SCREENING IN OLDER ADULTS (PRIMED STUDY)

Principal Investigator

**PI Name:**	**Sepucha, Karen , Ph.D**
**Phone:**	617-724-3350
**Primary email:**	KSEPUCHA@mgh.harvard.edu
**PI Institution:**	MGH
**PI Department/Unit:**	Mass General Brigham > MGH > Medical Services > General Internal Medicine

Protocol Information

**Protocol #:**	**2018P002848** **Related Protocol(s) #**
**Protocol Summary Version Date:**	01/24/23
**Protocol Version Name/Number:**	
**Protocol Version Date:**	
**Date of Initial IRB Approval:**	01/17/19
**Type of Protocol:**	Intervention/Interaction
**Overall Status:**	Active
**Expiration Date:**	**09/07/24**

Study Population

**Age Range:**	18 to 85
**Type of Subjects:**	Employees (physician, nurses, or other healthcare workers) in the course of, or related to, their employment related duties, Non-English Speakers
**Children Risk Assessment:**	

***Note: For research that presents greater than minimal risk and no direct benefit to the child, the permission of each child's parents or guardian will be sought unless one parent is deceased, unknown, incompetent, or not reasonably available, or when only one parent has legal responsibility for the care and custody of the child.**

Recruitment Process/Remuneration

**Source of Subjects:**	Medical Records, Primary Physician / Physician Specialist, Registries / Patient Databases (e.g. cancer registry), Research Patient Data Registry (RPDR)
**Recruitment Methods / Materials:**	Advertisements - E-Mail, Flyers / Postings (e.g. within BWH or MGH)
**Remuneration:**	Check, Gift Certificate
**Reimbursement:**	Other

Consent Process

**Informed Consent:**	
**Informed Consent Obtained by Whom:**	
**Informed Consent Obtained from Whom:**	
**Informed Consent of Non-English Speakers:**	
**Assent of Children:**	
**Participation of Subject Advocate:**	
**Less than 12 Hours to Give Consent:**	

Enrollment/Performance Sites

**Study-wide Enrollment:**	900
**Target Enrollment (Mass General Brigham sites):**	725
**Performance Site(s):**	BWH, MGH, NSMC, NWH, Off-site Research (off-site means sites other than those owned or controlled by Mass General Brigham entities)
**Reliance Agreements:**	

CR Enrollment	Date Recd	#Enrolled	#In Screen	#Ineligible	Active/On Study	#Completed	#Withdrew/Terminated/Lost/Other
CR1	12/26/19	90			82		2 / / 5 / 1
CR2	12/04/20	392			384		2 / / 5 / 1
CR3/AME42	10/19/21	539		3	466	70	2 / / 7 / 1
CR4/AME49	08/31/22	539		3		526	2 / / 7 / 1

Board	Process	Review Type	Review Date	Board Status	Expiration Date	Key Documents	Reconsent
IRB	IR	Expedited	01/17/19	Approve	01/17/20	PS	

Study Staff Amendment: Mayo, Denise NWH > Medicine > General Medicine Site Responsible Investigator Added
Study Staff Amendment: Siegel, Lydia NSMC > Medicine Site Responsible Investigator Added
Study Staff Amendment: Percac-Lima, Sanja MGH > Chelsea Health Center Co-Investigator Added
Study Staff Amendment: Kwiecien, Elaine MGH > Other Research Assistant Added
Study Staff Amendment: Lee, Vivian MGH > Other Research Coordinator/Manager Added
Study Staff Amendment: Ward, Abigail MGH > Medical Services Research Assistant Added
Study Staff Amendment: Licurse, Adam BWH > Medicine > Primary Care Site Responsible Investigator Added
Study Staff Amendment: Vo, Ha MGH > Medical Services > General Internal Medicine Research Assistant Added

Mass General Brigham

Board	Process	Review Type	Review Date	Board Status	Expiration Date	Key Documents	Reconsent
Study Staff Amendment: Marques, Felisha MGH > Medical Services > General Internal Medicine Research Coordinator/Manager Added							
Study Staff Amendment: Simmons, Leigh MGH > Medical Services > General Internal Medicine Co-Investigator Added							
Study Staff Amendment: Leavitt, Lauren MGH > Medical Services > General Internal Medicine Research Coordinator/Manager Added							
Study Staff Amendment: Sepucha, Karen MGH > Medical Services > General Internal Medicine Principal Investigator Added							
Study Staff Amendment: Atlas, Steven MGH > Medical Services > General Internal Medicine Co-Investigator Added							
Study Staff Amendment: Richter, James MGH > Medical Services > GI Unit Co-Investigator Added							
IRB	AME1	Administrative	02/04/19	Noted	01/17/20		
Protocol Amendment: Removing Study staff							
Study Staff Amendment: Ward, Abigail MGH > Medical Services Research Assistant Deleted							
IRB	AME2	Administrative	02/05/19	Noted	01/17/20		
Protocol Amendment: Add study staff							
Study Staff Amendment: Chang, Yuchiao MGH > Medical Services > General Internal Medicine Statistician Added							
Study Staff Amendment: Regan, Susan MGH > Medical Services > General Internal Medicine Data Coordinator/Manager Added							
IRB	AME3	Administrative	03/04/19	Noted	01/17/20		
Protocol Amendment: Adding study staff							
Study Staff Amendment: Valentine, Kathrene Mass General Brigham Regulatory Coordinator/Manager Added							
IRB	AME4	Expedited	04/11/19	Approve	01/17/20	PS	NA
Protocol Amendment: We modified the protocol summary to improve physician recruitment to include follow-up emails to physicians, a survey to screen physicians for eligibility, an EPIC report to assist with screening, and a study activity for physicians to review eligible patients. Thus, we added five new documents: reminder recruitment email, recruitment email from site champions, site-specific flyer for MMC, clinician eligibility screener survey, and a letter relating to the EPIC report. We updated the initial recruitment email, flyer, and clinician information sheet.							
IRB	AME5	Administrative	04/02/19	Noted	01/17/20		
Protocol Amendment: We are adding new study staff.							
Study Staff Amendment: Neil, Jordan MGH > Medical Services > Health Policy Center Research Coordinator/Manager Added							
IRB	AME6	Administrative	04/02/19	Noted	01/17/20		
Protocol Amendment: We are adding a new study staff.							
Study Staff Amendment: Shea, Liis MGH > Central Admin/COO President > Health SCI Library Research Assistant Added							
IRB	AME7	Expedited	05/10/19	Approve	01/17/20		NA
Protocol Amendment: We updated our physician initial baseline questionnaire with additional questions and removed questions to accurately capture our physician outcomes.							
IRB	AME8	Expedited	06/11/19	Approve	01/17/20	PS	No
Protocol Amendment: We made several edits in this Amendment: 1) New opt-out postcard for Maine Medical Center 2) Amended the protocol summary to reflect the opt-out postcard for Maine Medical Center 3) New patient cover letter for Maine Medical Center, adding language re: postcard 4) New patient cover letter for Brigham and Women's Hospital 5) Modified & added confidentiality language to the info sheets: patient, caregiver, clinician 6) Modified the patient cover letter language (invitation, 1 year later Mail, 1 year later Web, post visit)							
IRB	AME9	Administrative	06/07/19	Approve	01/17/20		
Protocol Amendment: We are adding new study staff member who will be supporting physician and patient enrollment from Maine Medical Center							
Study Staff Amendment: Scharnetzki, Elizabeth Maine Medical Center Research Coordinator/Manager Added							
IRB	AME10	Administrative	06/24/19	Noted	01/17/20		
Protocol Amendment: We are adding new study staff.							
Study Staff Amendment: Miller, Rebecca Mass General Brigham Research Assistant Added							
IRB	AME11	Administrative	07/02/19	Approve	01/17/20		
Protocol Amendment: We are adding study staff to support our efforts over the summer. The study staff recently joined our team.							
Study Staff Amendment: Martin, Hannah Maine Medical Center Research Assistant Added							
IRB	AME12	Expedited	08/20/19	Approve	01/17/20	PS	NA
Protocol Amendment: We are updating the protocol summary to deliver the incentive to participants who receive their survey by email after they have completed their survey. Consequentially, we updated the thank you email for those patients. Additionally, we edited the patient invitation cover letter and opt-out postcard for Maine Medical Center (MMC). Finally, we updated the caregiver survey, patient post-visit survey, and physician post-visit survey after receiving feedback from our Outcomes group.							
IRB	AME13	Expedited	09/04/19	Approve	01/17/20		NA
Protocol Amendment: We are adding an additional question to the patient survey.							
IRB	AME15	Expedited	11/05/19	Approve	01/17/20		NA
Protocol Amendment: We modified the email to patients about their post-visit survey.							
IRB	AME16	Expedited	11/05/19	Approve	01/17/20		NA
Protocol Amendment: We have included the email to send to physicians to complete the physician post-visit survey after their patient visit.							
IRB	AME14	Expedited	10/28/19	Approve	01/17/20	PS	NA
Protocol Amendment: We included a new thank you letter for caregivers who completed their survey by email. Additionally, we updated the recruitment criteria in the protocol summary to include more physicians to meet our enrollment targets.							
IRB	AME18	Expedited	12/02/19	Approve	01/17/20		No
Protocol Amendment: We updated the patient study invitation letter to be more clear and added the change to each letter unique to each site. We also updated the patient and caregiver survey by editing a question to be more clear on what we are trying to ask.							
IRB	AME19	Administrative	12/04/19	Noted	01/17/20		
Study Staff Amendment: Chen, Emily MGH > Human Resources Research Assistant Added							
IRB	AME21	Expedited	12/27/19	Approve	01/17/20		No
Protocol Amendment: We are adding a question (#17) about visit satisfaction that was included in previous versions of our survey.							
IRB	AME20	Expedited	12/17/19	Approve	01/17/20		NA
Protocol Amendment: we are adding the Spanish version of the cover letter invitation, information sheet and surveys for patients whose primary language is spanish							

Mass General Brigham

Board	Process	Review Type	Review Date	Board Status	Expiration Date	Key Documents	Reconsent
IRB	CR1	Expedited	01/08/20	Approve	01/17/21		
IRB	AME22	Administrative	01/02/20	Noted	01/17/20		
Study Staff Amendment: Miller, Rebecca Mass General Brigham Research Assistant Deleted							
IRB	AME23	Expedited	02/04/20	Approve	01/17/21		NA
Protocol Amendment: We have altered the language in our thank you letter to patients who mail back their survey and in our thank you letter to patients who email back their survey.							
IRB	AME24	Administrative	02/13/20	Approve	01/17/21		
Study Staff Amendment: Walsh, Kathleen Maine Medical Center Research Assistant Added							
IRB	AME25	Administrative	02/12/20	Noted	01/17/21		
Study Staff Amendment: Mancini, Brittney MGH > Medical Services > General Internal Medicine Research Coordinator/Manager Added							
IRB	AME27	Expedited	03/05/20	Approve	01/17/21		NA
Protocol Amendment: We are updating the patient invite packet to be more explicit about study activities as well as adding a new item to the physician post-visit survey.							
IRB	AME29	Expedited	06/01/20	Approve	01/17/21		NA
Protocol Amendment: We are adding additional questions to assess the impact of COVID-19 on patient's decision making process.							
IRB	AME30	Administrative	07/08/20	Noted	01/17/21		
Study Staff Amendment: Martin, Hannah Maine Medical Center Research Assistant Deleted							
IRB	AME31	Expedited	07/13/20	Approve	01/17/21	PS	NA
Protocol Amendment: We are adding a new patient recruitment process for the study during COVID-19, applicable at Mass General Brigham sites. We can no longer send an invitation letter to patients weeks in advance due to primary care physicians' scheduling changes due to COVID-19. We have created a new post-visit cover letter that includes language from the initial invitation letter (how the patient can opt out of the study if they wish), a new information sheet and we've updated the Protocol summary with these details.							
IRB	AME32	Administrative	08/17/20	Noted	01/17/21		
Study Staff Amendment: Robayo, Valeria Mass General Brigham Research Assistant Added							
Study Staff Amendment: Kwiecien, Elaine MGH > Other Research Assistant Modified							
IRB	AME33	Expedited	10/08/20	Approve	01/17/21	PS	No
Protocol Amendment: We have updated our 12-month patient survey to be conducted by phone. We've modified the survey questions and cover letter. The protocol summary has been updated to reflect this modification. We've also modified the Thank you cover letter that patients will receive after completion of the survey.							
IRB	AME34	Expedited	10/30/20	Approve	01/17/21		No
Protocol Amendment: We have modified the 12-month follow-up cover letter and survey to include: 1. included language that the phone survey will be audiotaped 2. added questions to assess patient's screening approaches if they chose "unsure" on the initial survey							
IRB	AME35	Administrative	12/03/20	Noted	01/17/21		
Study Staff Amendment: Shea, Liis MGH > Central Admin/COO President > Health SCI Library Research Assistant Deleted							
IRB	CR2	Expedited	12/10/20	Approve	12/10/21		
IRB	AME36	Administrative	02/08/21	Noted	12/10/21		
Study Staff Amendment: Kwiecien, Elaine MGH > Other Research Assistant Deleted							
IRB	AME37	Expedited	03/22/21	Approve	12/10/21		NA
Protocol Amendment: Adding a physician exit interview guide and survey. We've included a cover letter, reminder cover letter and updated the thank you letter.							
IRB	AME38	Expedited	04/05/21	Approve	12/10/21	PS	No
Protocol Amendment: We've added two additional $50 gift card lotteries for the participating physicians if they complete they exit survey and exit interview.							
IRB	AME39	Expedited	05/15/21	Approve	12/10/21		No
Protocol Amendment: Adding a new email template for use with the Maine Medical physician debrief invites, a new 'control' interview guide for use with the 'ctrointerview guide							
IRB	AME40	Administrative	06/15/21	Noted	12/10/21		
Study Staff Amendment: Chiang, Taylor Mass General Brigham Research Assistant Added							
IRB	AME41	Administrative	06/24/21	Noted	12/10/21		
Study Staff Amendment: Rieu-Werden, Meghan MGH > Medical Services > General Internal Medicine Research Assistant Added							
IRB	CR3/AME42	Expedited	10/21/21	Approve	10/21/22		
Protocol Amendment: Added DropBox business as a digital health tool for data sharing							
IRB	AME44	Expedited	01/13/22	Approve	10/21/22		
Study Staff Amendment: Doke, Emily Mass General Brigham Intern/Student Added							
IRB	AME45	Expedited	05/13/22	Approve	10/21/22		
Protocol Amendment: I need to remove a site PI as they are no longer affiliated with the site							
Study Staff Amendment: Siegel, Lydia NSMC > Medicine Site Responsible Investigator Deleted							
IRB	AME46	Expedited	05/20/22	Approve	10/21/22		
Study Staff Amendment: Robayo, Valeria Mass General Brigham Research Assistant Deleted							
Study Staff Amendment: Siar, Joshua Mass General Brigham Research Assistant Added							
Study Staff Amendment: Allen, Adrienne NSMC > General Internal Medicine Added							
Study Staff Amendment: Neil, Jordan MGH > Medical Services > Health Policy Center Research Coordinator/Manager Deleted							
Study Staff Amendment: Chen, Emily MGH > Human Resources Research Assistant Deleted							
Study Staff Amendment: Chiang, Taylor Mass General Brigham Research Assistant Deleted							
IRB	AME47	Expedited	07/08/22	Approve	10/21/22		
Protocol Amendment: Adding a new site responsible investigator for NSMC due to staffing change							
Study Staff Amendment: Allen, Adrienne NSMC > General Internal Medicine Site Responsible Investigator Added							
IRB	AME48	Administrative	08/31/22	Noted	10/21/22		

Mass General Brigham

Board	Process	Review Type	Review Date	Board Status	Expiration Date	Key Documents	Reconsent
Study Staff Amendment: Doke, Emily Mass General Brigham Intern/Student Deleted							
IRB	CR4/AME49	Expedited	09/07/22	Approve	09/07/24		
Protocol Amendment: I deleted the minor deviation log as instructed. it's saved in our records.							
IRB	AME50	Expedited	12/19/22	Approve	09/07/24		
Protocol Amendment: I need to remove a site responsible PI. I'm also adding a new study staff person.							
Study Staff Amendment: Licurse, Adam BWH > Medicine > Primary Care Site Responsible Investigator Deleted							
IRB	AME51	Administrative	01/24/23	Noted	09/07/24		
Study Staff Amendment: Balo, Joseph Mass General Brigham Research Assistant Added							

PARTNERS HUMAN RESEARCH COMMITTEE
PROTOCOL SUMMARY

Answer all questions accurately and completely in order to provide the PHRC with the relevant information to assess the risk-benefit ratio for the study. Do not leave sections blank.

PRINCIPAL/OVERALL INVESTIGATOR

Karen Sepucha, PhD

PROTOCOL TITLE

Promoting Informed Decisions about Cancer Screening in Older Adults (PRIMED Study)

FUNDING

Patient-Centered Outcomes Research Institute

VERSION DATE

March 29, 2021

SPECIFIC AIMS

Concisely state the objectives of the study and the hypothesis being tested.

As people age, medical decisions become more complex, including conversations about cancer screening. For patients aged 76-85, the United States Preventive Services Task Force (USPSTF) advises clinicians that decisions about colorectal cancer (CRC) screening should be individualized based on overall health and prior screening history (C recommendation).[i] However, studies find that many older adults are not well-informed about, nor meaningfully engaged in, decisions about whether to continue CRC screening. Shared decision making (SDM) has been shown to improve the quality of decisions about initiating cancer screening but little is known about its effectiveness for decisions about stopping interventions. This proposal addresses an important gap in our understanding of how to support clinicians and older patients in making good decisions about whether to continue CRC screening or not.

We will conduct a comparative effectiveness trial that will randomly assign clinicians at participating academic and community practices to one of two different decision support strategies. The first strategy (Registry arm) takes a population health management (PHM) approach and uses a patient registry to identify and track use of CRC screening among older adults for each clinician. The second strategy will enhance the registry by adding an established, multi-faceted SDM training program for clinicians (SDM Skills arm). We will enroll patients of participating primary care providers (PCPs), aged 76-85, who are due or overdue for CRC screening, and survey them shortly after an office visit to determine the impact of the two strategies on outcomes of importance to patients. We plan to randomly assign about 60 participating PCPs to the SDM skills or Registry arms, and enroll about 500 of their eligible patients. We will compare reports of shared decision making, patients' knowledge, and rates of patients who get their preferred option for CRC screening across study arms. We will also compare CRC screening rates across arms and to concurrent and historical controls. Through this project, we will accomplish the following specific aims:
Aim 1: Determine the impact of the approaches on patients' involvement in decision making and knowledge about the risks and benefits of continued CRC screening.

Hypothesis 1.1 (Primary outcome): Patients seen by clinicians in the SDM Skills arm will report more SDM discussions about cancer screening compared to the Registry arm.

Hypothesis 2.1 (Secondary outcome): Patients seen by clinicians in the SDM Skills arm will have higher knowledge of the benefits and harms of CRC screening and treatment compared to Registry arm.

Aim 2: Examine the effects of the interventions on patients' preferences for screening, the extent to which patients receive their preferred approach to screening, and on CRC screening rates.

Hypothesis 2.1 (Secondary outcome): A higher percentage of patients will receive their preferred approach to screening in SDM Skills arm compared to the Registry arm.

Hypothesis 2.2: (Secondary outcome) Both interventions will reduce CRC screening rates compared to concurrent controls (rates of clinicians who are not involved in study).

Aim 3: Examine the impact of the interventions on physicians' confidence with and skills for SDM in this setting.

Hypothesis 3.1: Clinicians in the SDM Skills arm will have higher confidence in their SDM skills.

Hypothesis 3.2: Clinicians in the SDM Skills arm will demonstrate more SDM skills in simulated patient interactions than the Registry arm.

The study will advance our understanding of how to best communicate evidence of cancer screening benefits and harms to older adults. Better decisions about whether or when to stop screening may reduce unnecessary tests and treatments and allow patients to avoid potential harms of screening.

BACKGROUND AND SIGNIFICANCE

Provide a brief paragraph summarizing prior experience important for understanding the proposed study and procedures.

Colorectal cancer (CRC) is a common, lethal disease that affects both men and women. In 2016, an estimated 135,000 people were diagnosed with CRC and 49,000 people died of it. The incidence of CRC increases with age, and the average age of diagnosis is 68 for men and 72 for women. CRC screening is widespread, and data indicate that 65% of adults over 65 were up-to-date with CRC screening tests; however, almost one quarter of adults 75 and older have never been screened for CRC. The screening is performed using a variety of methods, including stool-based testing requiring patients to collect stool specimens at home, and direct visualization testing such as colonoscopy. If stool-based testing is positive, then additional testing with a colonoscopy is recommended. There is evidence from observational and randomized trials that all methods for CRC screening are effective at reducing mortality attributed to colorectal cancer, provided the tests are conducted at recommended intervals with follow-up as needed.

Although CRC screening is recommended for adults aged 50-75, the USPSTF advises clinicians to make an individual decision for adults aged 76-85. Older adults often have a small potential benefit from screening and are at higher risk for complications, particularly complications of colonoscopy. The choice of whether to continue or stop screening depends significantly on patients' individual risk of colorectal cancer, their overall health, as well as their preferences for testing. Those who are able to undergo treatment if cancer is found and those who are otherwise healthy with long life expectancy may be more inclined to continue. Further, the USPSTF notes that adults aged 76-85 who have never been screened are more likely to benefit from CRC screening than those with prior testing.

There is a growing need to address appropriate use of cancer screening tests in older adults. Screening for asymptomatic disease comes with costs and potential harms. Shared decision making (SDM) is an established approach to engaging and informing patients in medical decisions. Currently, there is a lack of evidence on effective interventions to support clinicians in communicating with patients 76-85 about the benefits and harms of cancer screening and

tailoring decisions to what matters most to patients. This study will compare two established interventions to advance our understanding of how to support clinicians in conducting SDM conversations with older adults who may be considering stopping cancer screening.

RESEARCH DESIGN AND METHODS

Briefly describe study design and anticipated enrollment, i.e., number of subjects to be enrolled by researchers study-wide and by Partners researchers. Provide a brief summary of the eligibility criteria (for example, age range, gender, medical condition). Include any local site restrictions, for example, "Enrollment at Partners will be limited to adults although the sponsor's protocol is open to both children and adults."

The main study is a cluster randomized trial enrolling about 60 primary care clinicians across all sites. We estimate including about 30-35 clinicians from primary care practices affiliated with Massachusetts General Hospital (MGH) and Brigham and Women's Hospital (BWH), 10-15 from Maine Medical Center (MMC), and 10-15 from community practices affiliated with Newton Wellesley Hospital (NWH) and North Shore Medical Center (NSMC). The participating clinicians at each site will be placed into two groups stratified by gender and years of experience. Then, each group will be randomly assigned to one of two arms: Registry arm or Registry plus SDM Skills training arm (SDM arm). Clinicians in Registry arm will be notified about their patients aged 76-85 who are due for colorectal cancer screening with an upcoming visit. Clinician participants in the SDM arm will also receive the Registry notification and in addition, they will participate in a SDM skills course that includes online training and telephone-based simulated patient interactions.

After the training, study staff will track and enroll eligible patients who have upcoming visits with participating clinicians in both arms. We plan to enroll about 10 patients per clinician for a total of 500 patients. The clinicians will complete a short survey after each eligible patient visit. The patients will be invited to participate in the survey study and will be asked to complete a survey after their visit and another short survey one year later. Patients will be asked whether a spouse, friend or caregiver was involved in the decision-making process and if so, whether they would be willing to invite them to participate in the study. Study staff will follow up to enroll caregivers that patient participants identify and administer a short survey. We expect that about half of the patients will identify a caregiver, so about 250 caregivers will be surveyed.

Staff will track colorectal cancer screening for 12 months after the recorded date of visit for participating patients, for participating clinicians and for non-participating clinicians across these sites to examine trends in screening over time. Historical controls will also be collected to calculate rates of screening across the sites for the 2 years prior to the study (approximately calendar years 2017 and 2018).

The eligibility criteria for the clinician and patient participants are in Tables 1 and 2.

Table 1: Eligibility for clinician participants

Eligible	Ineligible
• Primary Care Physician (MD or NP)<br>• Have ≥20 potentially eligible patients in their panel<br>• Use of Epic electronic health record	• Residents, medical students

Table 2: Eligibility for patient participants

Eligible	Ineligible
• Adults, age 76-85 at the time of a scheduled visit<br>• Scheduled for non-urgent office visit with a participating clinician during the study period<br>• Due or overdue for colorectal cancer screening (e.g. never been screened, <1 year to follow-up interval indicated on last test).	• Prior diagnosis of colon or rectal cancer, inflammatory bowel disease or genetic disorder that raises CRC risk (hereditary non-polyposis CRC and familial adenomatous polyposis)<br>• Unable to consent for themselves (moderate to severe dementia or other major cognitive limitations)<br>• Unable to read or write in English or Spanish

Briefly describe study procedures. Include any local site restrictions, for example, "Subjects enrolled at Partners will not participate in the pharmacokinetic portion of the study." Describe study endpoints.

Clinicians will be recruited from primary care practices, both internal medicine and family medicine, affiliated with MGH, BWH, MMC, NWH and NSMC. The investigators will work with Steve Atlas who has an IRB approved protocol (2004P002796) to help identify the number of eligible patients by clinician and will then target those clinicians with high number of eligible patients for recruitment on the trial. IRB protocol 2004P002796 remains an active study and continues to operate per an IRB approved protocol and amendments. The study uses data from patients followed in MGH affiliated primary care practices for research and quality improvement purposes. The IRB protocol specifically pertains to research aspects that go beyond data used for administrative purposes as part of usual hospital operations. The study involves implementing a previously validated and published methodology to identify and link patients seen in MGH primary care practices to specific providers. Information is also collected on patient characteristics and outcomes of care. The data for this study is updated on a yearly basis. The information collected as part of this study can be made available to other IRB approved studies such as the current PRIMED submission if permitted in the IRB submission. Data collected as part of IRB protocol 2004P002796 will be used to identify MGH primary care providers with patients who meet eligibility criteria for the PRIMED submission. Though IRB protocol 2004P002796 will help the current submission with identifying potential primary care providers and patients, all aspects of contacting these providers and patients are covered in the current submission. Study staff will use similar methodology to identify the number of eligible patients by clinician for recruitment from the other Partners hospitals (BWH, NWH, NSMC) using RPDR and from MMC.

The participating clinicians at each site will be placed into two groups stratified by gender, years in practice, and number of clinic sessions per week. Then, each group will be randomly assigned to one of two arms: Registry arm or Registry plus SDM Skills training arm (SDM arm).

- Clinicians in Registry arm will complete a baseline telephone-based simulated patient interaction to evaluate their SDM skills. Once patient enrollment begins study staff will send periodic notification of their patients aged 76-85 who are due for colorectal cancer screening with an upcoming visit.
- Clinician participants in the SDM arm will complete an online SDM skills course, two telephone-based simulated patient interactions, and monthly facilitated case-based discussions. Once patient enrollment begins, study staff will send periodic notification of their patients aged 76-85 who are due for colorectal cancer screening with an upcoming visit.

Study staff will use existing functionality in the electronic medical record (EMR) via RPDR and Epic Reports to generate a list of patients 75 and older with CRC screening status, prior CRC screening results, and upcoming visit dates for each participating clinician. Staff will also work within the EMR at each site to develop an automated report of these items as available.

Our target is to enroll approximately 10 patients per clinician for a total of 500 patients. The clinicians will complete a short survey after each eligible patient visit. The patients will be invited to participate in the survey study and will be asked to complete a survey after their visit and another short survey one year later. Patients will be asked whether a spouse, friend or caregiver was involved in the decision-making process and if so, whether they would be willing to invite them to participate in the study. Study staff will follow up to enroll caregivers that patient participants identify and administer a short survey. We expect that about half of the patients will identify a caregiver, so about 250 caregivers will be surveyed.

The research coordinator will track the number of study participation invitations sent to each clinician as well as the number of clinicians indicating interest. We will track any reason given for refusal to join the study as well as any reason for dropping out of the study after randomization for reporting in CONSORT flow diagram. Staff will track completion of each activity (baseline survey, simulated patient interaction, training course, etc) for each clinician participant.

Staff will also track all patient participants screened, reason for ineligibility, the number sent invitation or post-visit survey packet by mail, the number who opted out or otherwise declined participation, those lost to follow-up for any reason, and any reasons given for the refusal to participate for reporting in CONSORT flow diagram. There are no formal written consent procedures in this study. The research presents no more than minimal risk of harm to subjects and involves no procedures for which written consent is normally required. Consent for the study will be implied by completion of the one survey for patient participants and email consent for clinician participants.

The primary outcome for this study is whether patients report more SDM in the visit with participating clinicians about CRC screening in the SDM arm compared to the Registry arm. Key secondary outcomes will be whether patients are knowledgeable about CRC screening benefits and harms, and whether clinicians understand patients' preferences and tailor screening decisions appropriately.

Patient reported measures: patients will complete a survey shortly after the visit.

- **Shared Decision Making Process (SDMP) Survey:** Four items assess the amount of shared decision making that occurs during a visit. These items are summed to generate a total score (0-4), with higher scores indicating greater patient involvement in decision making. The survey has been validated through its use in many studies, including two national studies of shared decision making for cancer screening and has strong evidence of acceptability, feasibility, reliability and validity.[Error! Bookmark not defined.][ii,iii] The s urvey has also been endorsed by National Quality Forum as a SDM performance measure (#2962).
- **Knowledge:** Five multiple choice knowledge items will assess patients' understanding of colorectal cancer screening adapted from the Colorectal Cancer Screening Decision Quality Instrument.[iv,v] A total knowledge score (0-5) will be calculated from the number of correct answers.
- **Risk perceptions:** One item will assess affective risk perception, or cancer worry. This item will be adapted from the National Cancer Institute's Health Information National Trends Survey (HINTS).[vi]

- **Patient's preferred approach to screening:** One item will assess patients' preferred approach to screening (with responses of colonoscopy, stool card test, no screening, not sure).
- **Overall health:** SF-1 will be used to assess patient's perception of overall health (poor to excellent)[vii]
- **Screening Recommendation and Time Spent:** One item will assess the patient's perception about their clinician's recommendation about CRC screening and one item will assess how much time was spent discussing CRC screening in the visit.
- **Single Item Literacy Screener**: One item that measures comfort with reading materials from health care providers. It has high specificity according to other, more detailed health literacy screening tools, and is able to be self administered.[viii,ix]
- **Demographics and CRC risk factors**: items will assess factors such as education, employment, marital status, family history of CRC, and alcohol use.
- **Barriers and facilitators to screening**: a subset of patients (those who did not receive their preferred approach to screening) will be surveyed again by phone about one year later to discuss any barriers or reasons why the preferred decision was not completed. For patients who had screening despite indicating desire to stop we will explore reasons for this change.

Study staff will supplement patient reported data with data collected via chart review. First, staff will review chart to confirm eligibility (e.g. age, dates and types of prior CRC screening tests as well as follow up test timing recommendation, CRC cancer history, dementia or cognitive impairment that would prevent participation, upcoming visit dates with participating clinician). Second, staff will access chart of participating patients to document CRC risk factors (e.g. family history, BMI, inflammatory bowel disease, diabetes, smoking status), abstract screening discussion in the visit note, subsequent CRC tests or procedures, complications related to CRC testing for participants, and findings of tests. Missing patient demographic information may also be supplemented via chart review.

Clinician, Practice and Network level CRC screening rates: We will use established, validated algorithms for calculating cancer screening rates using a combination of administrative, billing and clinical data. Dr. Atlas (co-I) led the algorithm development efforts at MGH and Partners, and he will work with the MMC team to ensure CRC screening rates are comparable across sites. Data is aggregated at the physician, practice and network (e.g. MGH, MMC, PCPO) level to identify the percentage of eligible patients up to date for screening during the historical control period, and concurrent observation period for study and non-study clinicians. Limited data on patient characteristics (e.g. age, gender, education, insurance status, etc) will be collected along with screening rates.

Clinician reported measures: clinicians will complete a baseline survey and a telephone-based simulated patient interaction (SPI) before staff start enrolling their patients onto the trial. They will complete a short survey after each eligible patient visit. After patient enrollment is complete, all clinicians will participate in a debrief interview.

- **Baseline clinician survey** will include the same CRC knowledge items as the patients and 3 items to assess clinicians' confidence in their ability to present benefits and harms, to discuss probabilities of benefits and harms and to elicit patients' goals and concerns during an office visit, each based on a five-point scale (not at all, a little, somewhat, very, and extremely confident).
- **Post visit survey:** 4 items SDMP survey will be adapted for use by clinicians, time spent discussing CRC screening in visit, recommendation, patient's preferred approach, and satisfaction with visit.

- **Baseline SDM skills assessment:** The telephone-based SPI will be transcribed and coded by two trained coders according to the well-validated Braddock's Informed Decision Making framework. The Braddock framework covers the core areas of SDM skills. [x,xi] A total score 0-9 will be calculated with higher scores indicating more SDM skills.
- **Debrief interview and survey**: After patient recruitment is complete, study staff will conduct a brief interview with all participating clinicians and will follow a structured interview guide to assess clinicians' attitudes toward SDM, their perceptions of the study and satisfaction with the intervention, and ideas for improvement. The clinicians will also be asked to complete a short, online survey to re-assess their confidence in their ability to present benefits and harms, to discuss probabilities of benefits and harms and to elicit patients' goals and concerns during an office visit.

Caregiver measures: a short survey post visit will contain SDMP survey, their perception of physician recommendation, their preferred approach to screening and their perception of the patient's preference for screening.

Adherence to intervention(s): The online training platform will track completion of modules and time spent on the webinar. Staff will track completion rates and time for the SPIs, delivery of the registry reports, participation in monthly case discussion sessions, and documentation in notes of CRC screening discussions in order to examine whether outcomes are affected by adherence to the protocol.
Patients will self-report time spent discussing CRC screening in the visit. Finally, staff will conduct a short follow-up survey with a subset of patients at 12 months to confirm screening choice and reasons for any discrepancy between preferred and implemented approach (e.g. transportation, insurance, clinician recommendation, spouse/caregiver preference, other new or worsening illness).

All study staff are Collaborative Institutional Training Initiative (CITI) certified and will receive training from the PI and program manager in the study protocol. We will hold regular meetings to review screening, enrollment and completion data, to discuss protocol and standard operating procedures, and to identify and mitigate any issues that arise.

For studies involving treatment or diagnosis, provide information about standard of care at Partners (e.g., BWH, MGH) and indicate how the study procedures differ from standard care. Provide information on available alternative treatments, procedures, or methods of diagnosis.

The standard of care is that physicians may discuss appropriate screening options, including their benefits and risks with each patient and individualize a decision based on the patients' risk, overall health and preferences. In this project, we will proactively remind clinicians to have these conversations with eligible patients about continuing colorectal cancer screening. Whether the physician and patient have this conversation remains at their discretion during a clinic visit. No tests or treatments will be administered as part of this study.

Describe how risks to subjects are minimized, for example, by using procedures which are consistent with sound research design and which do not unnecessarily expose subjects to risk or by using procedures already being performed on the subject for diagnostic or treatment purposes.

There are minimal risks to participating individuals associated with or attributable to this study. The main risks are associated with loss of privacy of their health information. To minimize risks, all electronic data files that include clinician, caregiver and patient identifiers will be kept in a

Partners protected server and only members of the research team will have access to the files. Files with PHI will only be accessed from Partners computers or encrypted laptops that are protected with SafeBoot. All patient information on eligibility screeners, chart reviews, and surveys collected at MMC will be sent securely using a secure file transfer to the Partners network. To ensure confidentiality, all paper surveys will be identified by study code number only and kept in a locked file cabinet and the scanned surveys and electronic files will be on password protected Partners server. Study papers (screeners, notes, surveys) that have been scanned or entered into a database will be disposed of in the confidential shredder. To address issues of psychological discomfort, research assistants will inform patients that they may refuse to answer any question and may withdraw from the study at any time. To address privacy and confidentiality issues, analytic database with outcomes data will not contain any identifying information and will be coded by unique study ID number only.

Patients will be invited to complete survey questionnaires. The time required for patient participants to complete each survey is about 15 minutes. Participants may opt out of the survey study, may refuse to answer any question (or set of questions) and may discontinue their participation at any time. It will also be emphasized that whether or not subjects participate will not impact the medical care that they receive. The caregiver's survey should take less than 10 minutes to complete.

The clinicians in the Registry arm will spend about 1.5 hours on study related surveys and activities and clinicians in the SDM arm will spend about 3.5 hours on study related activities over the course of 12-18 months. This includes the baseline questionnaire (10 minutes), the simulated patient interaction (20 minutes), and the training course for those assigned to SDM arm (about 2 hours). Clinicians will also complete about 10 surveys after patient visits that should take 1-2 minutes to complete. The exit interview will be about 20 minutes.

Describe explicitly the methods for ensuring the safety of subjects. Provide objective criteria for removing a subject from the study, for example, objective criteria for worsening disease/lack of improvement and/or unacceptable adverse events. The inclusion of objective drop criteria is especially important in studies designed with placebo control groups.

Although there are no written informed consent forms, Drs. Simmons and Sepucha are responsible for assuring that clinician and patient participants are adequately informed prior to engaging in any research procedures, that all subjects meet eligibility criteria, and that the study is conducted according to the IRB-approved research plan. The patient population in this study is older, and may have significant co-morbidities which may limit life expectancy. Staff will confirm status of patients, particularly before contacting patient participants for the follow-up survey.

There are no formal stopping rules for this minimal risk study.

FORESEEABLE RISKS AND DISCOMFORTS

Provide a brief description of any foreseeable risks and discomforts to subjects. Include those related to drugs/devices/procedures being studied and/or administered/performed solely for research purposes. In addition, include psychosocial risks, and risks related to privacy and confidentiality. When applicable, describe risks to a developing fetus or nursing infant.

There are minimal risks to individuals participating in this project. The main risks are the time and effort involved in participating and the potential loss of privacy. All electronic data files that include clinician, caregiver and patient identifiers will be kept in a Partners protected servers and only members of the research team will have access to the files. Files with PHI will only be accessed from Partners computers or encrypted laptops that are protected with SafeBoot. All patient information on eligibility screeners, chart reviews, and surveys collected at MMC will be sent securely using a secure file transfer to the Partners network. To ensure confidentiality, all paper surveys will be identified by study code number only and kept in a locked file cabinet and the scanned surveys and electronic files will be on password protected Partners server. Study papers (screeners, notes, surveys) that have been scanned or entered into a database will be disposed of in the confidential shredder. To address issues of psychological discomfort, research assistants will inform patients that they may refuse to answer any question and may withdraw from the study at any time. To address privacy and confidentiality issues, analytic database with outcomes data will not contain any identifying information and will be coded by unique study ID number only.

EXPECTED BENEFITS

Describe both the expected benefits to individual subjects participating in the research and the importance of the knowledge that may reasonably be expected to result from the study. Provide a brief, realistic summary of potential benefits to subjects, for example, "It is hoped that the treatment will result in a partial reduction in tumor size in at least 25% of the enrolled subjects." Indicate how the results of the study will benefit future patients with the disease/condition being studied and/or society, e.g., through increased knowledge of human physiology or behavior, improved safety, or technological advances.

All participating physicians will be notified of upcoming visits with eligible patients. SDM in the clinic visit has been shown to increase patient knowledge, reduce decisional conflict and improve the match between patients' preferences and their treatment choices. Those clinicians randomized to the SDM Skills arm may further benefit as prior work has shown the training results in increased confidence and competence in conducting SDM conversations with patients. Clinicians in both arms may benefit from the registry report as that may prompt them to discuss cancer screening with their older population of patients.

There are no direct benefits to patients from completing the surveys. The potential benefit to society is that the study will help determine the most effective approach to engaging and informing older patients about cancer screening.

As efforts to integrate SDM into routine care expand, understanding the effectiveness of interventions to achieve SDM is critical. This study will provide important new information on comparative effectiveness of different decision support strategies promoting SDM.

EQUITABLE SELECTION OF SUBJECTS

The risks and benefits of the research must be fairly distributed among the populations that stand to benefit from it. No group of persons, for example, men, women, pregnant women, children, and minorities, should be categorically excluded from the research without a good scientific or ethical reason to do so. Please provide the basis for concluding that the study population is representative of the population that stands to potentially benefit from this research.

The patient recruitment is limited to older men and women 76 to 85 years of age as the clinical guidelines on colorectal cancer screening highlight this age group as requiring an individual decision and thus are appropriate to engage in shared decision making. Children, younger adults, and pregnant women are not eligible for this decision. We will be targeting clinicians across the sites who care for a diverse patient population in order to increase enrollment of minority patients on the trial.

The clinician recruitment is focused on primary care clinicians (MD and NPs) who spend a significant portion of time seeing patients. Residents and other health care professional (e.g. RNs, social workers) are not eligible as they rarely consult patients regarding this decision.

When people who do not speak English are excluded from participation in the research, provide the scientific rationale for doing so. Individuals who do not speak English should not be denied participation in research simply because it is inconvenient to translate the consent form in different languages and to have an interpreter present.

Patient survey materials will be available in English, Spanish. We will try to include other languages based on the need of the population. Most patients seen at these sites speak either English or Spanish (>97%). Patients with other primary languages not translated for this study will be excluded from the survey portion of the study.

For guidance, refer to the following Partners policy:<br>Obtaining and Documenting Informed Consent of Subjects who do not Speak English<br>**https://partnershealthcare-public.sharepoint.com/ClinicalResearch/Non-English_Speaking_Subjects.1.10.pdf**

RECRUITMENT PROCEDURES

Explain in detail the specific methodology that will be used to recruit subjects. Specifically address how, when, where and by whom subjects will be identified and approached about participation. Include any specific recruitment methods used to enhance recruitment of women and minorities.

Clinicians will be recruited from primary care, internal medicine, and family medicine practices affiliated with MGH, BWH, MMC, NWH and NSMC. At MGH, there are about 190 adult primary care clinicians across 19 affiliated primary care practices, including 12 community-based practices and 3 hospital-based practices serving a diverse patient population in Eastern Massachusetts. Four of the practices are community health centers located in low-income urban communities around Boston and we plan to target clinicians at these centers to increase patient diversity. At MMC, there are 108 primary care providers at 10 affiliated primary care practices that will be screened for eligibility. Newton Wellesley Hospital and North Shore Medical Center have about 128 clinicians across 16 community practices. Current CRC screening rates for patients 76-85 range from 52%-65% at these sites. All participating sites use the Epic EMR with similar cancer screening registry functionality.

Clinician recruitment:

Investigators at each site will identify clinicians with a high volume of patients aged 76-85 and those who see patients at community health centers or centers with higher patient diversity. The recruitment and study procedure for clinicians is as follows:

1. The PIs and co-investigators will inform clinicians about the study and invite their participation in multiple ways: through presentations at clinical leadership meetings, practice meetings, through HDSC newsletter, through Partners Center for Population Health Newsletter, and through calls, meetings and emails to individual clinicians. Reminder emails will be conducted for non-responders within 2 weeks of the initial outreach.
2. Clinicians will indicate interest in participating in the study by contacting the PIs or study staff (either via email, phone or in person).
3. Study staff will contact interested clinicians to collect information to confirm eligibility and to support randomization using the screener questionnaire and will send an information sheet to clinicians that details the requirements of the study.
4. Clinicians will indicate their consent to participate by sending an email with their intention to join the study.
5. Study staff will then run an RPDR report to identify the clinicians' eligible patients due for colorectal cancer screening in the study year.
6. Eligible Clinicians will be randomly assigned to an arm by study statistician.
7. Clinicians assigned to the registry arm will have 4 weeks to complete:
    a. Baseline survey via RedCap or phone
    b. A telephone-based SPI conducted with standardized patients that will be audiotaped
    c. Short meeting with staff to review protocol and preferences for receiving the registry information about potentially eligible patients (e.g. 24h in advance of visit, weekly report, at morning huddle through medical asst)
    d. Review of their eligible patient list to indicate any patient that should not be approached for the study and a reason for exclusion.
    e. Study staff will send up to six reminder emails for each activity and make two reminder calls to encourage completion of each activity.
8. Clinicians assigned to the SDM training arm will have 4 weeks to complete:
    a. Baseline survey via RedCap or phone
    b. The online training course
    c. A telephone-based interaction conducted with a standardized patient. Study staff will email feedback 1 week after completion.
    d. Short meeting with staff to review protocol and preferences for receiving the information about potentially eligible patients (e.g. 24h in advance of visit, weekly report, at morning huddle through medical asst)
    e. Review of their eligible patient list to indicate any patient that should not be approached for the study and a reason for exclusion.
    f. Study staff will send up to six reminder emails for each activity and make two reminder calls to encourage completion of each activity.
9. Clinicians in the SDM Training arm will complete a second SPI about 8-12 weeks after the first and will have the opportunity to participate in monthly 'office hours' sessions—conference calls open to all participants in this arm that will be facilitated by SDM experts, primary care physicians and/or a gastroenterologist, to discuss cases and field questions and challenges that come up as they put the skills into practice.
10. After each eligible patient visit, staff will email a short questionnaire to participating clinicians. Staff will follow up with two reminder emails at 24 and 48 hours to complete the questions.
11. After patient enrollment is complete, staff will schedule an exit interview with clinicians. For participants who are not able to attend in person, the interview will be conducted by phone.

Patient and caregiver recruitment:

1. Study staff will review clinic schedules and medical records to identify eligible patients for participating clinicians prior to their scheduled visit. An Epic Report will be created to assist with this screening and eligibility review.
2. About two weeks before the visit, the research coordinator will send a cover letter signed by the participating clinician and an information sheet describing the study to all eligible patients. The cover letter will have information for participants who wish to opt out of the survey. Eligible patients at Maine Medical Center will also receive a post-card they may send back to opt-out of the study.
3. 1-3 days before the visit, staff will call all eligible patients who did not opt out to discuss the study and answer any questions. Staff will inquire about subject's preference to receive survey via email or mail. If email is preferred, staff will discuss privacy and obtain permission to send the survey via email without send secure (and confirm address). Study staff will read the following statement to patients, "The Partners HealthCare standard is to send email securely. This requires you to initially set up and activate an account with a password. You can then use the password to access secure emails sent to you from Partners HealthCare. If you prefer, we can send you "unencrypted" email that is not secure and could result in the unauthorized use or disclosure of your information. If you want to receive communications by unencrypted email despite these risks, Partners HealthCare will not be held responsible. Your preference to receive unencrypted email will apply to emails sent from this research study only. If you wish to communicate with other research staff at Partners regarding additional studies, your preference will have to be documented with each research group." After reading the required warning language, study staff will ask for the patient's verbal agreement. The agreement and agreement date will be noted in the research records. Finally, staff will determine whether a caregiver will be involved at the visit and if so, staff will obtain contact information for the caregiver.
4. After the visit, staff will send the patient a survey packet. The mailed survey packets will include a $5 incentive. Patients completing the survey via RedCap will receive an email with the direct link to the survey.
5. Patient consent for the study will be implied by return of the completed survey.
6. Staff will also send a survey packet to the caregiver if the patient identified one. Staff will make up to three reminder phone calls in an attempt to get a response and may complete the survey via the phone. Reminder packets will not be sent to caregivers.
7. Staff will make up to three reminder phone calls (or emails for patients who preferred email). For those who don't respond to the initial survey and reminders, study staff will send all patients one reminder paper survey packet. For patients who initially received the email link, the paper packet will include their $5 incentive to complete the survey. Study staff will then make up to 3 additional reminder calls to non-responders. Patients will be given the option to complete the survey by phone.
8. Approximately one year after initial visit with PCP, a research coordinator will confirm status of each patient and will document any colorectal cancer screening tests and procedures completed since the visit from the medical record.
9. Staff will identify patients who did not receive their preferred approach to colon cancer screening and will mail a letter notifying patients about the second survey. The mailed letter notifying patients about the survey ~~packet~~ will contain the $5 incentive.
10. Study staff will follow a similar protocol as with the initial survey by making up to three phone call attempts to reach the patient and administer the survey by phone.
11. All participants who complete a survey will receive a thank you note.

Patient and caregiver recruitment during COVID-19:

Given the COVID-19 pandemic, we have to make modifications to our patient recruitment process. Physician schedules have been in flux; with most patient appointments being converted to a telehealth visit and confirmed about a week before the visit. Given this new scheduling process, we can no longer screen patients weeks in advance of the visit, nor send an invitation letter to the patient about the study. Instead, we will screen visits about a week before the visit and send the patients the invitation cover letter, information sheet and survey after their visit occurs. The process is outlined below:

1. About a week before scheduled visits, study staff will review clinic schedules and medical records to identify eligible patients for participating clinicians. An Epic Report will be created to assist with this screening and eligibility review.
2. After confirming the visit occurred, staff will send the patient a survey packet including an invitation cover letter signed by the participating clinician, an information sheet describing the study, the survey, and a $5 incentive. The cover letter will have information for participants who wish to opt out of the survey. The cover letter will also have the REDCap online link for patients who wish to complete the survey online.
3. Patient consent for the study will be implied by return of the completed survey.
4. For patients who didn't opt out, staff will make up to three reminder phone calls for the initial survey packet.
5. For those who don't respond to the initial survey and reminders, study staff will send all patients one reminder paper survey packet. Study staff will then make up to 3 additional reminder calls to non-responders. Patients will be given the option to complete the survey by phone.
6. Staff will also send a survey packet to the caregiver if the patient identifies one. Reminder phone calls will not be conducted and reminder packets will not be sent to caregivers.
7. Approximately one year after initial visit with PCP, a research coordinator will confirm status of each patient and will document any colorectal cancer screening tests and procedures completed since the visit from the medical record.
8. Staff will send a letter about the second survey to all patients who are still alive and who did not receive their preferred approach to screening. The mailed packet will contain the $5 incentive.
9. Study staff will make up to three phone calls to administer the survey by phone for all non responders. All participants who complete a survey will receive a thank you note.

All study staff are CITI certified and will receive training from the PI and program manager in the study protocol. We will hold regular meetings to review screening, enrollment and completion data.

Provide details of remuneration, when applicable. Even when subjects may derive medical benefit from participation, it is often the case that extra hospital visits, meals at the hospital, parking fees or other inconveniences will result in additional out-of-pocket expenses related to study participation. Investigators may wish to consider providing reimbursement for such expenses when funding is available

- At MGH, BWH, NWH, NSMC and MMC:
    - All clinician participants will receive a total of $100.
    - Clinicians in the SDM skills arm will receive 2-3 hours of risk management CME credit, as well as MOC Part II credit.
    - Clinicians' name will be entered into a quarterly lottery for a $50 amazon gift card each time they complete a survey on one of their patient participants.

- Clinicians’ name will be entered into a lottery for a $50 amazon gift card upon completion of the exit survey.
- Clinicians’ name will be entered into a lottery for a $50 amazon gift card upon completion of the exit interview.

- Patients and caregivers will receive a $5 incentive with each survey.

For guidance, refer to the following Partners policies:

Recruitment of Research Subjects
https://partnershealthcare-public.sharepoint.com/ClinicalResearch/Recruitment_Of_Research_Subjects.pdf

Guidelines for Advertisements for Recruiting Subjects
https://partnershealthcare-public.sharepoint.com/ClinicalResearch/Guidelines_For_Advertisements.1.11.pdf

Remuneration for Research Subjects
https://partnershealthcare-public.sharepoint.com/ClinicalResearch/Remuneration_for_Research_Subjects.pdf

CONSENT PROCEDURES

Explain in detail how, when, where, and by whom consent is obtained, and the timing of consent (i.e., how long subjects will be given to consider participation). For most studies involving more than minimal risk and all studies involving investigational drugs/devices, a licensed physician investigator must obtain informed consent. When subjects are to be enrolled from among the investigators’ own patients, describe how the potential for coercion will be avoided.

There are no formal written consent procedures in this project for either patients, clinicians, or the non-intervention group. The research presents no more than minimal risk of harm to subjects and involves no procedures for which written consent is normally required. Clinician consent will be implied by email indicating interest and patient and caregiver consent for the study will be implied by completion of the first questionnaire.

Clinicians will be provided a written information sheet that describes the requirements of the study and will be instructed to send an email to the PIs or study staff indicating their interest in participating. By agreeing to be on the study, clinicians will give consent to study staff to identify and contact their eligible patients.

Eligible patient participants will be given an information sheet that describes the risks and benefits of the study and a cover letter inviting them to participate in the survey. The invitation will include information about how to opt out of the survey portion of study. Eligible patient participants at Maine Medical Center will also receive a post-card that may send back to opt-out of the study. Eligible patients sent a survey packet during the COVID-19 pandemic will also see information about how to opt out of the survey portion of the study in the invitation cover letter. Patient subjects will be given 7 days to review the material and opt out by calling or emailing the study staff. Participants who do not opt out will be contacted by phone by research staff and can indicate their decision to accept or decline participation when contacted. Consent will be implied by the return of the completed survey.

The principal investigators’ names and contact information will be available on the information sheet if participants have any questions or concerns about the study. The study staff will be

available by phone or email to discuss the study and answer any questions. Each site has at least one primary care physician co-investigator who will be available by pager and study staff/PI will be available by phone to answer any questions.

Patients and caregivers will give verbal consent if they wish to receive surveys via unencrypted emails. The IRB information regarding send-secure vs. unencrypted emails will be included on the invitation and research staff will discuss this with the patients when they join the study. Patients and caregivers may also be read the IRB policy and ask for verbal consent to receive unencrypted emails over the phone. Participants can choose to receive the surveys via a send-secure email or on paper in the mail if they do not wish to receive unencrypted emails. Participants during the COVID-19 pandemic will all receive a paper survey and may choose to complete the survey online via the REDCap link provided in the cover letter.

Study materials will emphasize that whether or not patients participate will have no effect on the health care they receive.

NOTE: When subjects are unable to give consent due to age (minors) or impaired decision-making capacity, complete the forms for Research Involving Children as Subjects of Research and/or Research Involving Individuals with Impaired Decision-making Capacity, available on the New Submissions page on the PHRC website:
https://partnershealthcare.sharepoint.com/sites/phrmApply/aieipa/irb

For guidance, refer to the following Partners policy:
Informed Consent of Research Subjects:
https://partnershealthcare-public.sharepoint.com/ClinicalResearch/Informed_Consent_of_Research_Subjects.pdf

DATA AND SAFETY MONITORING

Describe the plan for monitoring the data to ensure the safety of subjects. The plan should include a brief description of (1) the safety and/or efficacy data that will be reviewed; (2) the planned frequency of review; and (3) who will be responsible for this review and for determining whether the research should be altered or stopped. Include a brief description of any stopping rules for the study, when appropriate. Depending upon the risk, size and complexity of the study, the investigator, an expert group, an independent Data and Safety Monitoring Board (DSMB) or others might be assigned primary responsibility for this monitoring activity.

NOTE: Regardless of data and safety monitoring plans by the sponsor or others, the principal investigator is ultimately responsible for protecting the rights, safety, and welfare of subjects under his/her care.

The data sources will be clinician surveys, transcripts of simulated patient interactions, patient surveys, caregiver surveys and, electronic health record information.

There are no foreseeable safety risks to participants for participating in a simulated patient interaction or completing a survey. Study staff will protect the privacy of research study participants as described in the Privacy and Confidentiality section. It is possible that participants may be upset by a question in the interview or survey, although our experience with similar questionnaires in other topics (including breast cancer decision making and decision

making for joint replacement surgery) have found that it is rare for participants to be upset. Nevertheless, study staff will screen for adverse events and address them as described in the next section.

Study data will be accessible at all times for the Co-PIs to review. The project manager and co-PIs will examine study conduct including enrollment, accrual, drop-outs, and protocol deviations on a weekly or every other week basis with the staff at each site. Study staff will review study related data including comments from the SPIs, reminder phone calls to participants, participant surveys and will notify the PI about any serious or moderate potential adverse events (AEs) immediately and any minor or potential ones at regular meetings. The Co-PIs will review AEs individually real-time and in aggregate on a regular basis at team meetings. No SAEs are expected based on the minimal risk trial. However, the Co-PIs and co-investigators will review potentially serious adverse events (SAEs), as soon as they are discovered. The Co-PIs will ensure all protocol deviations, AEs, and SAEs are reported to the IRB within required time frame based on severity, and will file an HRC AE Form within 10 working days as needed.

There are no formal stopping rules for this minimal risk study.

Describe the plan to be followed by the Principal Investigator/study staff for review of adverse events experienced by subjects under his/her care, and when applicable, for review of sponsor safety reports and DSMB reports. Describe the plan for reporting adverse events to the sponsor and the Partners' IRB and, when applicable, for submitting sponsor safety reports and DSMB reports to the Partners' IRBs. When the investigator is also the sponsor of the IND/IDE, include the plan for reporting of adverse events to the FDA and, when applicable, to investigators at other sites.<br><br>NOTE: In addition to the adverse event reporting requirements of the sponsor, the principal investigator must follow the Partners Human Research Committee guidelines for Adverse Event Reporting

No serious adverse events are expected. The name and contact information for the principal investigator will be included on study information sheet as well as contact for study staff and MGH IRB in case participants have a problem. We will have a clinical co-investigator for each topic who will be able to consult on any clinical issues that arise during the course of the interviews or surveys. However, if a serious adverse event occurs relating to the study, then the principal investigator will report the event to the IRB within 24 hours and will file an HRC Adverse Event Form within 10 working days. If a mild or moderate adverse event occurs, the principal investigator will summarize the event in the progress report at continuing review.

Study staff will be instructed to review surveys within 48 hours of receipt and to notify the PI about any potentially serious events immediately and all other events at regularly scheduled meetings. Study staff will keep records of any feedback, questions, concerns and/or complaints that are received and we will address them with the co-investigators and staff as needed.

MONITORING AND QUALITY ASSURANCE

Describe the plan to be followed by the principal investigator/study staff to monitor and assure the validity and integrity of the data and adherence to the IRB-approved protocol. Specify who will be responsible for monitoring, and the planned frequency of monitoring. For example, specify who will review the accuracy and completeness of case report form entries, source documents, and informed consent.

NOTE: Regardless of monitoring plans by the sponsor or others, the principal investigator is ultimately responsible for ensuring that the study is conducted at his/her investigative site in accordance with the IRB-approved protocol, and applicable regulations and requirements of the IRB.

The study staff and the principal investigator at each site will have routine meetings during the study period to ensure the project proceeds as intended per the protocol. All participant screening and enrollment will be tracked on password protected servers using an Access or RedCap database. The information is stored behind a firewall and only study staff will have access to it as needed. We will track recruitment rates and response rates weekly and identify issues as they come up. The study staff will complete all required documents for the study binder and this will be reviewed quarterly by the project manager and one of the principal investigators.

Limited data will be kept on clinician non-responders for those who received an individual invitation (site, age, gender, patient volume and years in practice) as well as patient non-responders including age, gender, physician, and all elements in the eligibility screener. This information will be used to examine non-response bias.

For guidance, refer to the following Partners policies:

Data and Safety Monitoring Plans and Quality Assurance
https://partnershealthcare-public.sharepoint.com/ClinicalResearch/DSMP_in_Human_Subjects_Research.pdf

Reporting Unanticipated Problems (including Adverse Events)
https://partnershealthcare-public.sharepoint.com/ClinicalResearch/Reporting_Unanticipated_Problems_including_Adverse_Events.pdf

PRIVACY AND CONFIDENTIALITY

Describe methods used to protect the privacy of subjects and maintain confidentiality of data collected. This typically includes such practices as substituting codes for names and/or medical record numbers; removing face sheets or other identifiers from completed surveys/questionnaires; proper disposal of printed computer data; limited access to study data; use of password-protected computer databases; training for research staff on the importance of confidentiality of data, and storing research records in a secure location.

NOTE: Additional measures, such as obtaining a Certificate of Confidentiality, should be considered and are strongly encouraged when the research involves the collection of sensitive data, such as sexual, criminal or illegal behaviors.

Special efforts will be made to protect the privacy of subjects. We will review the subject's medial record to confirm eligibility to participate in the study. We will have names and addresses of eligible participants and this information will be kept separate from the study data (e.g. surveys and/or interview notes). All participants--patients, clinicians and caregivers--will receive a code number and the surveys and other data will only be identified by code number. A

separate password-protected electronic file will contain the codes linked to identifying information. Only the MGH study staff and investigators will have access to this file. These will be kept as long as required by the research project. After the study has been completed the personal contact information of all eligible participants will be destroyed.

All files (e.g. eligibility screeners) that contain PHI will be kept in a locked file cabinet or in a secure offsite file storage location or on a password protected Partners shared drive.

Patient confidentiality will be maintained as is routine for all patient care privacy guidelines. All research staff are CITI certified and will be trained on the importance of data confidentiality.

SENDING SPECIMENS/DATA TO RESEARCH COLLABORATORS OUTSIDE PARTNERS

Specimens or data collected by Partners investigators will be sent to research collaborators outside Partners, indicate to whom specimens/data will be sent, what information will be sent, and whether the specimens/data will contain identifiers that could be used by the outside collaborators to link the specimens/data to individual subjects.

To promote research replicability, transparency and future use of the data, de-identified data sets will be created and will be available, by request, to outside researchers. After the study results have been published, de-identified data sets will also be deposited in an open access service such as, ICPSR (https://www.icpsr.umich.edu/icpsrweb/). On ICPSR, individuals must register and agree to ICPSR's Responsible Use statement prior to accessing datasets. Additionally, before a dataset is made available for access, ICPSR completes a detailed review of all datasets to assess disclosure risk. If necessary, ICPSR modifies data to reduce disclosure risk or limits access to datasets for which modifying the data would substantially limit their utility or the risk of disclosure remains high. No information that contains identifiers or that could be used to link an individual to the data will be included in the de-identified data set. The information sheets will contain the following language: After the study is completed, all identifiable information will be removed from the data and after removal, the de-identified information will be deposited in an open access service to promote use of the data by other researchers.

Specifically address whether specimens/data will be stored at collaborating sites outside Partners for future use not described in the protocol. Include whether subjects can withdraw their specimens/data, and how they would do so. When appropriate, submit documentation of IRB approval from the recipient institution.

No identifiable data on Partners patients will be stored outside MGH. MMC patient data that is collected outside Partners will be received by the MGH research team (see details below).

MMC will have access to the clinician participant data across all sites, as needed, to schedule and conduct the simulated patient interactions. Any emails that contain identifiable clinician data will be sent using SendSecure, and any large files will be sent using secure file transfer. MMC will only have access to de-identified data sets for the Partners patients and caregivers.

RECEIVING SPECIMENS/DATA FROM RESEARCH COLLABORATORS OUTSIDE PARTNERS

When specimens or data collected by research collaborators outside Partners will be sent to Partners investigators, indicate from where the specimens/data will be obtained and whether the specimens/data will contain identifiers that could be used by Partners investigators to link the specimens/data to individual subjects. When appropriate, submit documentation of IRB approval and a copy of the IRB-approved consent form from the institution where the specimens/data were collected.

Eligibility screeners, patient, caregiver and clinician surveys, and medical record information will be collected from participants at MMC, by MMC-affiliated study staff. This patient health information is necessary for assessing patient eligibility for participation and for administering the study protocol. As such, this patient information will be sent from MMC to MGH study staff via a secure file transfer or REDCap. The paper surveys collected at MMC will be scanned and sent to the MGH research team using a secure file transfer, and the paper copies will be transported for ultimate storage or confidential disposal at MGH.

All electronic files that contain patient identifiers will be kept Partners protected servers and will only be accessed with Partners computers or encrypted laptops.

Eligibility and medical chart review data will be collected via REDCap (Research Electronic Data Capture). REDCap is a free, secure, HIPAA compliant web-based application hosted by the Partners HealthCare Research Computing, Enterprise Research Infrastructure & Services (ERIS) group. The system offers easy data manipulation with audit trails, reports for monitoring and querying participant records, and an automated export mechanism to common statistical packages (SPSS, SAS, Stata, R/S-Plus).

---

[i] US Preventive Services Task Force. Screening for colorectal cancer US Preventive Services Task Force Recommendation Statement. The Journal of the American Medical Association 2016;315(23):2564-2575.

[ii] Hoffman T, Lewis CL, Pignone MP, et al. Decision-making processes for breast, colorectal, and prostate cancer screening: the DECISIONS survey. *Medical Decision Making*. 2010;30(5 Suppl):53S-64S.

[iii] Sepucha K, Stringfellow V, Fowler FJ. Shared Decision Making (SDM) Process Survey: Validity and Reliability of a Short, Patient-Reported Measure of SDM. 2017 Society for Medical Decision Making Annual Meeting abstract.

[iv] Sepucha K, Feibelmann S, Cosenza C, Levin CA, Pignone M. Development and evaluation of a new survey instrument to measure the quality of colorectal cancer screening decisions. *BMC medical informatics and decision making*. 2014;14(1):72.

[v] Hoffman RM, Elmore JG, Pignone MP, Gerstein BS, Levin CA, Fairfield KM. Knowledge and values for cancer screening decisions: Results from a national survey. Patient Educ Couns. 2016 Apr;99(4):624-30.

[vi] Health Information National Trends Survey. https://hints.cancer.gov/ Accessed December 5, 2016.

[vii] DeSalvo KB, Bloser N, Reynolds K, He J, Muntner P. Mortality Prediction with a Single General Self-Rated Health Question: A Meta-Analysis. *Journal of General Internal Medicine*. 2006;21(3):267-275. doi:10.1111/j.1525-1497.2005.00291.x.

[viii] Morris NS, MacLean, CD, Chew LD, Littenberg B. The Single Item Literacy Screener: evaluation of a brief instrument to identify limited reading ability. *BMC Family Practice*. 2006;7(1):1.

[ix] Brice JH, Foster MB, Principe S, Moss C, Shofer FS, Falk RJ, DeWalt DA. Single-item or two-item literacy screener to predict the S-TOFHLA among adult hemodialysis patients. *Patient education and counseling*. 2014;94(1):71-75.

[x] Leader A, Daskalakis C, Braddock C, Kunkel EJ, et al. Measuring Informed Decision Making about Prostate Cancer Screening in Primary Care. Med Dec Making. 2011; 32(2):327-36.
[xi] Price EL, Bereknyei S, Kuby A, Levinson W, Braddock CH. New elements for informed decision making: a qualitative study of older adults' views. Patient education and counseling. 2012;86(3):335-341.

PCORI RESEARCH PLAN TEMPLATE

RESEARCH STRATEGY

A. Specific Aims

As people age, medical decisions become more complex, including conversations about cancer screening. For patients aged 76-85, the United States Preventive Services Task Force (USPSTF) advises clinicians that decisions about colorectal cancer (CRC) screening should be individualized based on overall health and prior screening history (C recommendation).[1] However, studies find that many older adults are not well-informed about, nor meaningfully engaged in, decisions about whether to continue CRC screening. Shared decision making (SDM) has been shown to improve the quality of decisions about initiating cancer screening but little is known about its effectiveness for decisions about stopping interventions. This proposal addresses an important gap in our understanding of how to support clinicians and older patients in making good decisions about whether to continue CRC screening.

We will conduct a comparative effectiveness trial that will randomly assign clinicians at participating academic and community practices to one of two different decision support strategies. The first strategy (Registry arm) takes a population health management (PHM) approach and uses a patient registry to identify and track use of CRC screening among older adults for each clinician. The second strategy will enhance the registry by adding an established, multi-faceted SDM training program for clinicians (SDM Skills arm). We will enroll about 500 patients, aged 76-85, who are due or overdue for CRC screening, and survey them shortly after an office visit to determine the impact of the two strategies on outcomes of importance to patients. We will examine whether the interventions increase shared decision making, patients' knowledge, and ensure patients get their preferred option for CRC screening. We will also examine the impact on CRC screening rates. Through this project, we will accomplish the following specific aims:

Aim 1: Determine the impact of the approaches on patients' involvement in decision making and knowledge about the risks and benefits of continued CRC screening.

Hypothesis 1.1 (Primary outcome): Patients seen by clinicians in the SDM Skills arm will report more SDM discussions about cancer screening compared to the Registry arm.

Hypothesis 2.1 (Secondary outcome): Patients seen by clinicians in the SDM Skills arm will have higher knowledge of the benefits and harms of CRC screening and treatment compared to Registry arm.

Aim 2: Examine the effects of the interventions on patients' preferences for screening, the extent to which patients receive their preferred approach to screening, and on CRC screening rates.

Hypothesis 2.1 (Secondary outcome): A higher percentage of patients will receive their preferred approach to screening in SDM Skills arm compared to the Registry arm.

Hypothesis 2.2: (Secondary outcome) Both interventions will reduce CRC screening rates compared to concurrent controls (rates of clinicians who are not involved in study).

Aim 3: Examine the impact of the interventions on physicians' confidence with and skills for SDM in this setting.

Hypothesis 3.1: Clinicians in the SDM Skills arm will have higher confidence in their SDM skills.

Hypothesis 3.2: Clinicians in the SDM Skills arm will demonstrate more SDM skills in simulated patient interactions than the Registry arm.

The study will advance our understanding of how to best communicate evidence of cancer screening benefits and harms to older adults and is well aligned with PCORI's mission. Better decisions about whether or when to stop screening may reduce unnecessary tests and treatments and allow patients to avoid potential harms of screening. Although this project focuses on cancer screening decisions, the findings may be applicable to many other decisions about continuing or stopping medical tests or treatments for older adults such as statins for primary prevention of heart disease, bisphosphonates for fracture prevention, and anticoagulants for stroke prevention in patients with atrial fibrillation.

B. Background

Colorectal cancer (CRC) is a common, lethal disease that affects both men and women. In 2016, an estimated 135,000 people were diagnosed with CRC and 49,000 people died of it.[2] The incidence of CRC increases with age, and the average age of diagnosis is 68 for men and 72 for women.[3] CRC screening is widespread, and data indicate that 65% of adults over 65 were up-to-date with CRC screening tests; however, almost one quarter of adults 75 and older have never been screened for CRC.[4,5] The screening is performed using a variety of methods, including stool-based testing requiring patients to collect stool specimens at home, and direct visualization testing such as colonoscopy. If stool-based testing is positive, then additional testing with a colonoscopy is recommended. There is evidence from observational and randomized trials that all methods for CRC screening are effective at reducing mortality attributed to colorectal cancer, provided the tests are conducted at recommended intervals with follow-up as needed.[6]

Although CRC screening is recommended for adults aged 50-75, the USPSTF advises clinicians to make an individual decision for adults aged 76-85. Older adults often have a small potential benefit from screening and are at higher risk for complications, particularly complications of colonoscopy.[7] The choice of whether to continue or stop screening depends significantly on patients' individual risk of colorectal cancer, their overall health, as well as their preferences for testing. Those who are able to undergo treatment if cancer is found and those who are otherwise healthy with long life expectancy may be more inclined to continue. Further, the USPSTF notes that adults aged 76-85 who have never been screened are more likely to benefit from CRC screening than those with prior testing.[1]

As of the 2014 census, there were over 14 million people ages 76-85 in the United States, and it is projected that will grow to 21 million people by 2025.[8] **There is a growing need to address appropriate use of cancer screening tests in older adults.** Screening for asymptomatic disease comes with costs and potential harms. The harms of colorectal cancer screening include the risk of perforation or bleeding with colonoscopy, and harms related to the colonoscopy bowel preparation (inconvenience and nausea). Other considerations include the direct costs of the test, follow up on abnormal findings, as well as the less-often recognized costs related to transportation to medical appointments, time off work for patients and caregivers, and the physical discomforts of testing and treatment.[9] As highlighted by our patient partners, these concerns can be magnified for seniors, who live on limited incomes and may have additional illness or frailty that make screening and treatment more challenging.

There are growing efforts to encourage clinicians to avoid the overuse of testing and treatments that are considered "low-value," with the most prominent of such efforts being the Choosing Wisely® campaign.[10,11] Recently, the American College of Surgeons, American Society of Clinical Oncology, Society of General Internal Medicine, and the American Society of Nephrology all recommended against routine cancer screening in patients with life expectancy of less than 10 years.[12] This recommendation is based on evidence that patients with higher comorbidity burden have less benefit from cancer screening compared to those of the same age who are healthy.[13] However, this recommendation, based solely on life expectancy, may not result in truly patient-centered care.

There is tremendous heterogeneity of cancer risk, functional status, and life expectancy in older adults, factors which should influence decision making about continued CRC screening. Older patients who are at higher risk for complications of colonoscopy may consider switching to a less invasive test or may decide to stop altogether, depending on their ability to complete follow up testing or treatment if indicated. Working with our patients, primary care clinicians and gastroenterologists, we have identified different features that will impact decision making including screening history, results of prior screens (positive or negative), overall health, patient preferences and clinician preferences. **Table 1** details three patient cases with different features to illustrate the challenges that may arise and the need for shared decision making.

Studies have found considerable gaps in clinicians' ability to inform and meaningfully involve older patients in cancer screening decisions.[14,15,16] Despite the fact that the majority of older adults (89%) want to be involved in deciding about continuing or stopping cancer screening, only 49% reported talking to their doctor about the options.[17] Addressing age-appropriate cancer screening is a required component of the Initial Preventive Physical Examination and the Medicare Annual Wellness Visit (AWV), the Medicare-funded yearly reviews of patients' health status and establishment of an age-appropriate Medicare Part B Preventive Services screening schedule.[18,19] In other words, all primary care clinicians should be having these conversations with their older patients, but studies suggest this is not happening as often as patients would like.

Studies have also identified several clinician barriers to high quality CRC screening decisions such as inadequate clinician knowledge of guidelines, lack of training, bias of clinicians toward colonoscopy over other screening options, and lack of systematic strategies (e.g. reminder systems, performance reports) to support CRC screening discussions and implementation.[20,21,22,23] These conversations require patients and clinicians to prioritize multiple, potentially competing health goals, discuss competing hazards for mortality, and engage patients who may have mild to moderate cognitive impairment. Careful attention to how these conversations happen will be important to ensure high quality, patient-centered decisions.

Table 1: Three cases highlighting the need for SDM across different clinical scenarios

**Case 1 Features**<br>Prior Screening and findings: None<br>Overall health: Good<br>Patient Preference: Not sure<br>Doctor Preference: Screening	**Situation**: *76 year-old woman, recently moved to town to be closer to her daughter and help with childcare for her grandkids. She has never had any colorectal cancer screening.*<br>**Patient's view**: *I know screening is good but I can't ask my daughter to take off work to help me. Not sure that I can do this now.*<br>**Doctor's view**: *She is very healthy and would probably benefit from screening. FIT/FOBT testing might be good start, but would need to make sure she is willing/able to have colonoscopy if we find something.*
**Case 2 Features**<br>Prior screening and findings: Colonoscopy every 10 years, all negative<br>Overall health: Fair to poor<br>Patient Preference: Screening<br>Doctor Preference: No screening	**Situation**: *83 year-old woman with rheumatoid arthritis is extremely frail, her mother was diagnosed with colon cancer at 90, past colonoscopies were normal. She lives alone.*<br>**Patient's view**: *I don't want to be another statistic—someone who could have prevented dying from colon cancer. I didn't find my prior colonoscopies too difficult.*<br>**Doctor's view**: *Missing a diagnosis is not the statistic I am worried about. I'm concerned about her high risk of complications and about her safety with the prep, she lives alone and there is a risk of falling. Her mother's diagnosis does not make her at higher risk and given prior negative findings there is little benefit from continued screening.*
**Case 3 Features**<br>Prior screening and findings: Colonoscopy every 5 years, polyp<br>Overall health: Average<br>Patient preference: No screening<br>Spouse preference: Screening<br>Doctor preference: Screening	**Situation**: *81 year-old man with heart disease, arthritis with prior spine and hip surgery, has had an abnormal polyp 6 years ago and is overdue for follow up colonoscopy.*<br>**Patient's view**: *It would be very hard for me to go through the cleanout regimen at home. Why should I do anything at this point?*<br>**Wife's view**: *He has a hard time with the prep, but I'd rather go through that than cancer.*<br>**Doctor's view**: *The prior polyp was pretty abnormal, a tubulovillous adenoma. I think he could do the prep with some help from his wife. With his age and heart disease, if he has cancer, having extensive colon surgery would carry significant risk. Continuing with colonoscopy may help remove precancerous polyps and prevent advanced cancer.*

SDM is promising, but there is a gap in evidence regarding its effectiveness for decisions about stopping screening. SDM is an established approach to engaging and informing patients in medical decisions. The use of patient decision aids (pDAs) to promote SDM has been well studied in decisions about initiating cancer screening. Four published studies have examined use of decision aids in CRC screening for patients 50-75, and all resulted in improved knowledge while two studies found the pDA increased screening rates and two found no effect on screening uptake.[24,25] However, there are no existing decision aids to support CRC screening decisions with older adults; thus, other approaches are needed.

Cancer screening registries that are built into electronic health records (EHR) are common and effective in increasing appropriate use of screening for patients aged 50-75.[26,27] However, after age 75, patients are typically excluded from these registries. Extending registries to include this older population may prompt clinicians to engage more older adults in discussions about screening. We selected an EHR-enabled registry as one of our interventions to examine the impact in this population. However, the registry alone may not be enough to overcome the barriers to SDM. As a result, we added clinician training in SDM skills to the registry for our comparator intervention.

This proposal identifies an important, prevalent and challenging clinical decision and the results will fill a key evidence gap. Currently, **there is a lack of evidence on effective interventions to support clinicians in communicating with patients 76-85 about the benefits and harms of cancer screening and tailoring decisions to what matters most to patients.** This innovative proposal will compare two established interventions to advance our understanding of how to support clinicians in conducting SDM conversations with older adults who may be considering stopping cancer screening.

C. Significance

Though most patients and physicians are accustomed to discussing when to begin cancer screening, the conversation on when and whether to stop cancer screening may raise anxieties. Limited life expectancy is often an underlying issue, and bringing up mortality is not always expected, nor welcome, at a routine office visit. In addition, physicians report many barriers to using SDM for discussions on whether to stop cancer screening, such as lack of time to engage patients, limited knowledge of evidence for screening in older patients, and general discomfort with stopping or withdrawing care. The majority of patients report that they do not have these discussions with their doctor, and are not involved as much as they would like to be in these decisions.[28] One study with older adults found that they are very open to stopping screening, but many did not understand the role of life expectancy and objected to its use in the discussion. The study recommended clinicians avoid giving reasons phrased with life expectancy, e.g. "you will not live long enough to benefit from this test," and instead simply say "the test will not help you live longer."[29] **Without skills to elicit patient concerns and guide a balanced conversation about potential benefits and harms of screening, there is the potential that some patients will undergo tests that they would not want if fully informed. Conversely, other patients could be denied testing that they would have preferred to continue, if they had been given a voice in the decision.**

The study will address key gaps in the literature and will answer questions that are aligned with PCORI's mission, and that are important to patients and clinicians. The two main research questions that we will address in this proposal are: (1) How do we ensure that older adults are engaged in and informed about decisions regarding continuing colorectal cancer screening? and (2) What is the comparative effectiveness of two established, widely available decision support strategies on the quality of decisions and rates of CRC screening with older adults? Our patient partners, who were involved in designing the study, identified several important outcomes. The primary outcome is whether the interventions result in more SDM conversations about CRC screening. Other important outcomes include patient knowledge of benefits and harms and evidence that physicians tailored recommendations to patents preferences. Our patient and clinician partners also emphasized that better decisions about whether or when to stop screening may reduce unnecessary tests and allow patients to avoid potential harms of screening. Although this project compares effectiveness of decision support for cancer screening decisions, the findings will be applicable to a range of other decisions for older adults about stopping of medical tests or treatments.

D. Study Design or Approach. The study design, interventions and outcome measures were selected in collaboration with patient partners, clinician stakeholders, and researchers. The study is a cluster randomized trial and the PCORI methodology standards, CONSORT standards and the Standards for Universal Reporting of Decision Aid Evaluation Studies (SUNDAE) guidelines have been followed to ensure scientific rigor.[30,31,32]

D.1. Conceptual frameworks and prior studies. The SDM skills training intervention is based on the conceptual framework of shared decision making as outlined in Mulley[33] and Sepucha and Mulley,[34,35] which views SDM as a systems approach to enable continuous improvement in clinical decision making. Core elements of the framework guide the content of the training, including (1) recognizing a decision, (2) presenting options, (3) discussing pros and cons of each option, (4) eliciting patients' goals and preferences, (5) facilitating deliberation to identify the best option and (6) supporting implementation.[34,36,37,38] The SDM Skills training provides physicians concrete approaches to each of these steps. The SDM conceptual framework has guided the training intervention as well as the selection of outcomes, including SDM, knowledge and patient's preferences.

Cancer screening across the United States is increasingly supported through population health initiatives within organizations. Thus, the proposed research also builds on the **Chronic Care Model (CCM)** that provides a health systems structure for organizing care to improve outcomes. In the CCM, the central goal of interventions to change care is to enable productive interactions between informed and engaged patients and prepared and proactive providers.[39,40] The Zapka/Taplin Framework provides a cancer specific model that outlines the multi-level influences on cancer care delivery, including the patient, health care providers, and practice setting, and identifies where failures can occur to inform interventions.[41,42,43] Based on these frameworks, we have engaged patients, primary care clinicians, gastroenterologists who perform colonoscopies, and population health management teams who oversee cancer screening initiatives in the design of the study. In addition, we have selected our intervention and comparator to use information technology (IT) to facilitate patient identification, remind clinicians to have these conversations, and to provide training to clinicians in SDM skills. Finally, we have the support of leadership (see Letters from **Metlay, Chaguturu, Chamberlain, St. Germain, Weil**) at the organization and practice level to ensure successful conduct of the study and dissemination of results.

The Massachusetts General Hospital (MGH) team has developed a webinar on SDM skills training that provides 2 hours of Continuing Medical Education credits (CME). The webinar was based on in-person training sessions that received high satisfaction ratings and resulted in clinician behavior change, increasing use of patient decision aids.[44,45] The MGH team recently conducted a study with 18 physicians to evaluate the impact of the webinar. **The training was feasible and acceptable, as the majority of physicians (89%) completed the intervention including the telephone-based simulated patient interactions (SPI), with written feedback on their SPI**. The training resulted in significant improvements in SDM skills as evidenced by telephone-based simulated patient interactions. For example, the percentage of physicians who elicited patients' goals nearly doubled (from 47% at baseline to 82% after the webinar).[46] Co-investigators at Maine Medical Center (MMC) have also developed and delivered SDM training courses.[47,48] Their SDM training curriculum includes an e-learning module, didactic lecture material, clinical training and evaluation tools (including Observed Structured Clinical Examination cases using standardized patients), and faculty training, among different CME programs. **The PIs and co-investigators have extensive experience delivering SDM training using online modules and standardized patients and will adapt an existing, available intervention that has been shown to be effective in increasing clinicians' skills in SDM.**

> *It was really nice to have that [video of the] patient's encounter and have the doctor rate herself too. It helps to know what's being measured when you're looking at shared decision making. Because it's something that we all think we do- hopefully some of us do it.*
>
> –Physician after viewing the SDM webinar

D.2. Interventions: The study will compare two established approaches to support decisions about stopping CRC screening for older adults.

Comparator 1 (Registry only arm): Clinicians across the country routinely receive reports from registries indicating CRC screening status for their patients aged 50-75 and these data often form the basis of quality measures.[26,27] For this project, we will extend the CRC screening registry to include patients 76-85. Participating clinicians will receive a report indicating patients with an upcoming visit who are due for screening along with results of prior screens (**Appendix A**

includes a sample report generated using existing functionality). Staff will meet with each clinician before recruitment to review registry functionality and preferred workflow for receiving reports. Clinicians in this arm will not receive any formal SDM training. They will complete one SPI to assess baseline skills, but will not receive feedback on performance.

Comparator 2 (SDM Skills arm): CME is an established approach to advance clinicians' knowledge and skills. In this arm, clinician participants will receive the patient registry reports described above and will participate in a multi-component training course in SDM skills. The training is available online and participants will receive 2 hours of CME for completing it. The course was designed with input from patients and clinicians and incorporates evidence-based features that have been shown to result in physician behavior change including case studies, interactive exercises, as well as simulated patient interactions (SPIs).[49] Participants will receive written feedback on their SPIs. The online training and telephone-based SPIs will provide flexibility for clinicians to complete the modules when convenient for them. In addition to having been very highly-rated by clinician participants, the existing training program been shown to be effective in promoting SDM skills in simulated patient interactions and increasing use of patient decision aids.[44,45] Investigators will make adaptations to the existing webinar to incorporate examples and evidence on CRC risk factors, risks and benefits of screening, considerations of co-morbidity, functional status and life expectancy, and risks and benefits of treatment if cancer is found. The existing training covers how to effectively elicit and respect patients' goals and concerns, how to engage a spouse and/or caregiver, and how to uncover and address barriers to implementation. **Appendix B** includes screen shots from the existing webinar and the content outline for the adapted version.

D.3. Study Design: We will conduct a cluster randomized trial at the clinician level. In the study, participating clinicians within three organizations (MGH, MMC, and Partners Community Physician's Organization (PCPO)) will be randomized into two groups stratified by gender and years of experience. Then, each group will be randomly assigned to one of two arms: Registry arm or SDM Skills training. Because the interventions are delivered at the clinician-level, randomization at the patient-level is not feasible. The risk of contamination with clinician-level randomization is low, since it is not likely that clinicians assigned to different study arms will share enough details with one another to impact outcomes. Due to the nature of the interventions, clinicians will not be blinded to their study arm. Patient participants will be blinded to their clinician's assignment. The patient surveys will be identified by code only, so study staff doing data entry and statistician doing data analysis will be blinded to study arm.

D.4. Study Protocol

D.4.a. Project activities and timeline: Figure 1 includes the CONSORT diagram with estimates for enrollment and numbers for the primary outcome. Staff will track details about the randomization, screening and enrollment estimates and ensure systematic sampling of eligible patients. A full study protocol will be developed in collaboration with co-investigators, biostatistician and our patient partners and stakeholders in the first quarter of the project.

Table 2 summarizes the major activities for the comparative effectiveness study. After administrative tasks are completed we will begin recruitment. Clinicians will be assigned to an arm and given four weeks to complete the baseline assessment, including the telephone-based SPI. Clinicians assigned to the training arm will also complete the webinar in that time. Clinicians will receive $100 or the practice will receive a stipend for participation. More details on clinician recruitment process are included in the Human Subjects Section. Once patient enrollment begins, study staff will provide regular reports of CRC screening status for each clinician's 76-85 year-old patient population with an upcoming visit. Study staff will survey eligible patients approximately one week after the visit until they receive 10 patient responses per clinician. The patient survey protocol will follow a modified Dillman approach to ensure high response rates.[50,51] The mailed survey packets will include a small incentive ($5). Staff make reminder phone calls, send a reminder mailing, and then make additional reminder calls to non responders. Research staff will document use of CRC screening from the medical record in the 12 months following the visit. Most patients will complete screening within 3 months if they plan to follow through. However, we selected a 12-month window to minimize the number of patients

falsely categorized as having stopped screening when, in fact, their screening has simply not yet occurred. Staff will also follow up at 12 months with patients to explore screening choice and reasons for any discrepancies between preferred and actual screening.

Table 2: Key project activities and timeline

Months 0-6	• Webinar updated, standardized patients trained, study protocol finalized and IRB approval across sites.
Months 6-9	• Clinicians enrolled and randomized, training completed for those in SDM skills arm, baseline survey and simulated patient interviews completed for all clinician participants.
Months 9-21	• Patient enrollment starts. Study staff will ensure registry reports are delivered, screen and enroll eligible patients, collect post-visit patient surveys.
Months 21-33	• Debrief interviews for participating clinicians, study staff will collect screening status for participants, and conduct follow-up with patient participants.
Months 34-36	• Analysis and write up of results, dissemination of results to study participants.

D.4.b. Webinar adaptation and simulated patients: In the first six months of the project, we will adapt the existing MGH SDM Skills training webinar. We will conduct structured interviews with 20 patients (10 men and 10 women) who are 76-85, about half of whom are still screening and half of whom decided to stop screening, to elicit their understanding of the role of cancer screening, the benefits and harms of continued screening, and their experiences discussing the decision whether to screen with their primary care clinicians. We plan to include 2-3 participants who have been diagnosed with colorectal cancer after age 75 to understand experiences with screening and treatment. From these interviews, we will identify common misperceptions that may need to be addressed, key information that should be conveyed, and goals and concerns. We will use these findings to prepare cases that illustrate key teaching points in the webinar and to create realistic but challenging simulated patient cases. The cases will highlight different features such as (1) prior screening history (2) prior findings (3) co-morbidity/overall health and (4) patient's preferences and goals. Table 1 shows how these different features may be combined into cases. Our patient partners and clinician co-investigators will work with the PIs on the simulated patient (SP) cases to ensure they are clinically accurate, and are realistic from the patient's perspective. We will train the professional SPs and have them practice delivering the case over the phone with three clinician co-investigators. Once the SPs feel comfortable with the content, they will practice with two more clinicians using the same telephone-based protocol as the formal study. Each practice case will be audiotaped, transcribed, and reviewed to ensure consistency in the delivery of the cases.

D.5. Study population and settings: Clinicians will be recruited from primary care, internal medicine, and family medicine practices affiliated with MGH in Boston, MA; MMC in Portland, ME; and PCPO in MA. At MGH, there are about 190 adult primary care clinicians across 19 affiliated primary care practices, including 12 community-based practices and 3 hospital-based practices serving a diverse patient population in Eastern Massachusetts. Four of the practices are community health centers located in low-income urban communities around Boston and we plan to target clinicians at these centers to increase patient diversity. At MMC, there are 108 primary care providers at 10 affiliated primary care practices that will be screened for eligibility. The PCPO has a network adult and pediatric clinicians in community practices across MA. For this study, we will recruit from two systems within the PCPO that have 128 clinicians at 16 community practices. Current CRC screening rates for patients 76-85 range from 52%-65% at these sites. All participating sites use the Epic electronic health record (EHR) with similar cancer screening registry functionality. Letters of support from clinical leaders (Metlay (MGH), Chamberlain (MMC), Weil (PCPO)) and from the medical directors of each MGH health center (Morrill, Pasinski, Stratton and Xerras) confirm access to the clinician population.

Rationale for selection of sites: These sites were selected to provide a mix of academic and community clinicians, in suburban and urban settings. In addition, they have strong investigator experience with SDM, strong leadership support,

large potential pool of eligible clinicians and patients, and existing electronic health records that can support the registry reports. These features are highlighted in the CCM conceptual framework as necessary for successful implementation.

For this study, we will recruit 60 primary care clinicians (about 30-35 from MGH, 10-15 from MMC, and 10-15 from PCPO) from primary care practices affiliated with the three networks. To be eligible, clinicians need to have completed their training and have ≥30 potentially eligible patients in their panel. Administrative data indicate there are 291 clinicians across the sites that meet these criteria. Further, the average number of patients aged 76-85 per eligible clinician is about 70. (These data were used for the estimates in Table 3). At MGH, we plan to target clinicians in our 4 health centers to increase patient diversity and planned enrollment is in **Human Subjects** (**p.20**).

Figure 1: CONSORT Flow diagram

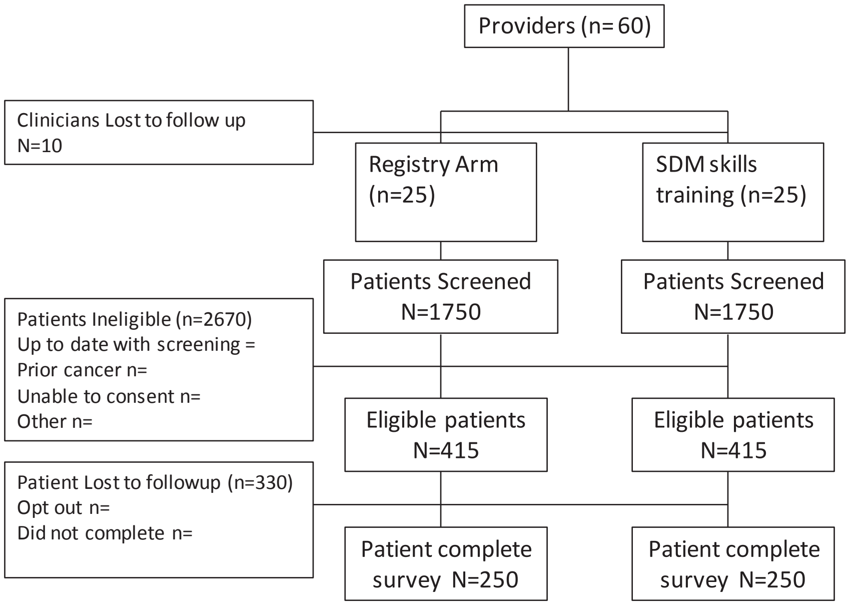

Our goal is to receive 500 completed patient surveys (250 per arm). We will approach patients from participating clinicians until we reach 10 completed surveys. Eligible patients will be aged 76-85 years old, have a scheduled visit with a participating clinician during the study period, and be ***due*** for CRC screening (e.g. never been screened, <1 year to follow-up interval indicated on last test). Patients who have a prior diagnosis of colon or rectal cancer, inflammatory bowel disease or genetic disorder that raises colon cancer risk, including hereditary non-polyposis colorectal cancer and familial adenomatous polyposis, do not speak English or Spanish, have severe cognitive impairment, or are scheduled for an urgent visit, will be excluded. We estimate that about 60% will complete the post-visit survey; therefore, we need to approach 830 patients to meet the target. The response rate for the patient survey was based on prior experience with MGH patients (n=652) who were surveyed about one week after a visit using a similar protocol that achieved a response rate of 70.4%.[52] However, the patients in that study were on average of 62 years old, and we lowered our estimate based on older population for this study.

Table 3. Recruitment Plan for Prospective Studies (site specific breakdowns are in **Appendix C).**

	**Clinicians**	**Patients**
1. Estimated number of potentially eligible study participants (Clinicians: administrative data; Patients: EHR and admin data)	426	3500
2. Total number of study participants expected to be screened:	426	3500
3. Total number of study participants expected to be eligible of those screened:	291	830
4. Target sample size (use same number stated in milestones):	50	500
5. If applicable, total number of practices or centers that will enroll participants:	3 networks	3 networks
6. Projected month first participant enrolled (month after project initiation):	6	9
7. Projected month last participant enrolled (month after project initiation):	9	21
8. Projected rate of enrollment (number enrolled per month of enrollment period):	15	42
9. Estimated percentage of participant dropout:	15%	n/a

D.6. Sample size and Power: We plan to recruit 60 clinicians (mostly physicians but may include nurse practitioners) to participate. Assuming an 15% drop out rate, this number will ensure that we reach our target of 50 clinicians. Administrative data indicates that the eligible clinicians will have on average, 70 patients aged 76-85 in their panel, and we expect to screen those 3500 patients. Data from EHR and GI colleagues suggests that there will be 17 eligible patients per clinician, or 830 patients overall, and we estimate that roughly 60% will complete the post-visit survey. This response rate will result in 500 completed surveys, or approximately 250 per arm. Our estimate for intraclass correlation coefficient (ICC) was 0.023 from a previous study. Assuming a more conservative ICC of 0.03, the effective sample size will be 394 and will enable us to detect a difference of 0.28 standard deviation (SD) for the primary outcome with 80% power. **Section D.9** contains effective sample sizes and power calculations for the other hypotheses.

D.7. Outcomes: Our patient partners feel that the most important and relevant outcome for this study will be whether patients report more SDM in the visit with participating clinicians about CRC screening. Key secondary outcomes will be whether patients are knowledgeable and whether clinicians listen to patients' preferences and tailor screening decisions appropriately. Our clinician partners are interested in whether the interventions will reduce overall screening rates and have expressed concerns about whether it will take too much time to have these conversations. Accordingly, we plan to ask patients how long the discussion was and to survey clinicians in both arms to assess satisfaction. The outcomes are assessed using a mix of established, well-validated patient and clinician surveys, as well as EHR data.

Primary outcome, patient reported:

- **Shared Decision Making Process (SDMP) Survey:** Four items assess the amount of shared decision making that occurs during a visit. These items are summed to generate a total score (0-4), with higher scores indicating greater patient involvement in decision making. The survey has been validated through its use in many studies, including two national studies of shared decision making for cancer screening and has strong evidence of acceptability, feasibility, reliability and validity.[16,53,54] The survey has also been endorsed by National Quality Forum as a SDM performance measure (#2962).

Secondary outcomes and covariates:

- **Knowledge:** Five multiple choice knowledge items will assess patients' understanding of colorectal cancer screening from the Colorectal Cancer Screening Decision Quality Instrument.[55,56] A total knowledge score (0-5) will be calculated from the number of correct answers.
- **Risk perceptions:** One item will assess affective risk perception, or cancer worry. This item will be adapted from the National Cancer Institute's Health Information National Trends Survey (HINTS).[57]
- **Patient's preferred approach to screening:** One item will assess patients' preferred approach to screening (with responses of colonoscopy, stool card test, no screening, not sure).
- **Overall health:** SF-1 will be used to assess patient's perception of overall health (poor to excellent)[58]
- **Screening Recommendation and Time Spent:** One item will assess the clinician's recommendation about CRC screening and one item will assess how much time was spent discussing CRC screening in the visit.
- **Single Item Literacy Screener**: One item that measures comfort with reading materials from health care providers. It has high specificity according to other, more detailed health literacy screening tools, and is able to be self administered.[59,60]
- **Demographics and CRC risk factors**: items will assess factors such as education, employment, marital status, family history of CRC, and alcohol use.

Study staff will conduct chart review to confirm eligibility, document CRC risk factors (e.g. BMI, inflammatory bowel disease, diabetes, smoking status), screening discussion in the visit note, and use of CRC screening tests for participants.

Clinician, Practice and Network level CRC screening rates: We will use established, validated algorithms for calculating cancer screening rates using a combination of administrative, billing and clinical data. Dr. Atlas (co-I) led the algorithm development efforts at MGH and Partners, and he will work with the MMC team to ensure rates are comparable across

sites. Data is aggregated at the physician, practice and network (e.g. MGH, MMC, PCPO) level to identify the percentage of eligible patients up to date for screening during the observation period for study and non-study clinicians.

Each clinician will complete a baseline survey and a telephone-based SPI before staff start enrolling their patients onto the trial. After enrollment is complete, all clinicians will participate in a debrief interview.

- **Baseline clinician survey** will include the same CRC knowledge items as the patients and 3 items to assess clinicians' confidence in their ability to present benefits and harms, to discuss probabilities of benefits and harms and to elicit patients' goals and concerns during an office visit, each based on a five-point scale (not at all, a little, somewhat, very, and extremely confident).
- **Baseline SDM skills assessment:** The telephone-based SPI will be transcribed and coded by two trained coders according to the well-validated Braddock's Informed Decision Making framework. The Braddock framework covers the core areas of SDM skills. [61,62] A total score 0-9 will be calculated with higher scores indicating more SDM skills.
- **Debrief interview**: After patient recruitment is complete, study staff will conduct a brief interview with all participating clinicians and will follow a structured interview guide to assess clinicians' attitudes toward SDM, their perceptions of the study and satisfaction with the intervention, and ideas for improvement.

Adherence to intervention(s): The online platform will track completion of modules and time spent on the webinar. Staff will track completion rates and time for the SP interactions, delivery of the registry reports, and documentation in notes of CRC screening discussions in order to examine whether outcomes are affected by adherence to the protocol. Patients will self-report time spent discussing CRC screening in the visit. Finally, staff will conduct a short follow-up survey with patients at 12 months to confirm screening choice and reasons for any discrepancy between preferred and implemented approach (e.g. transportation, insurance, clinician recommendation, spouse/caregiver preference).

D.8. Data sources: The main data sources are patient and clinician surveys, the transcribed SPIs and electronic health record. The surveys may be administered by paper, over telephone or online and all variables will be coded consistently across modes to enable reliable analysis. The SPIs will be audiorecorded, using common functionality available with our conference call system, and the audiofiles will be transcribed. The MGH team will create an Access database that will be used across sites to support systematic screening of patients, tracking reasons for ineligibility and nonresponse, and tracking implementation of study protocols to ensure consistency of data collection across sites. The MGH team will create RedCap surveys (web-based, HIPAA compliant system) to support data entry for chart reviews and paper surveys.

D.9. Analytic plan: As in all research that uses surveys, the key assumption is the obtained sample represents the underlying population. As the first step to check this assumption, responders and non-responders will be compared across groups to examine non-response bias. Sample demographics and characteristics will be compiled and compared across groups to evaluate the randomization and whether the groups are well balanced. Reasons for non-response will be tracked. We will use established methods to maximize survey response rates, including financial incentives and a series of phone and mail reminders. As indicated earlier, we anticipate 60% response rate for patients. For patient reported outcomes (e.g. knowledge, shared decision making), missing data will be handled according to established protocols for the validated surveys. For item-specific analysis, our primary analyses will be conducted excluding patients with missing data. The following describes the analyses for main hypotheses. The hypotheses will be evaluated using an **intention to treat approach** and patients will be analyzed based on their assigned arm and not on whether the intervention took place (i.e. clinician completed the training).

Hypothesis 1.1*: (Primary) Patient reports of shared decision making will be higher in the SDM Skills arm.* We will first examine the distribution of the SDM Process score and apply variable transformation techniques if necessary. We will use the Generalized Estimating Equations (GEE) techniques to account for the patients within clinician data structure.[63] As described earlier (see **D.6.**), we will be able to difference of 0.28 SD for the SDM Process score with 80% power. Studies using the SDM Process survey have found effect sizes ranging from 0.39SD – 0.88SD when comparing sites that

used have formal decision support (coaching or decision aids) and those that did not.[54] Although we have sufficient power to detect a smaller difference, we are interested in a meaningful difference 0.4SD or higher.
Hypothesis 1.2*: Patients knowledge will be higher in the SDM Skills arm compared to the Registry arm.* As in Hypothesis 1.1, the target sample size will provide 80% power to detect a difference of 0.28 SD for the knowledge scores after adjusting for the effects of clustering. Although there are no standards for a minimally important difference in knowledge, SDM studies have found effect sizes of 0.25SD-0.8SD in studies of decision aids, and this study is adequately powered to detect a meaningful difference in that range.[64]
Hypothesis 2.1*: A higher percentage of patients will receive their preferred approach to screening in SDM Skills arm.* For these analyses, we will compare the percentages of patients who prefer to stop screening, the percentages of informed patients who received their preferred screening in the 12 months after the visit, and overall rates of screening across the two groups using logistic regression model with the GEE approach to adjust for clustering of patients within clinicians. We will have 81% power to detect a difference of 14% in rates (e.g. decrease from 61% to 47%).
Hypothesis 2.2: *Both interventions will reduce screening rates compared to concurrent controls (rates of clinicians not involved in study).* The screening data will be available for all patients from all participating sites. We estimate a total of 6248 patients will be eligible for screening from 436 clinicians: 5353 patients from clinicians not participating the study and 448 from each arm of clinicians participating the study. After taking into account clustering effect, we will have at least 86% power to detect a 6% decrease in CRC screening rates of patients from clinicians participating in the study compared to patients from clinicians not participating.
Hypothesis 3.1: *SDM Skills arm will have higher clinician confidence in their ability to conduct SDM conversations.* Clinician confidence will first be analyzed as a continuous variable. Variable transformation will be performed to improve normality assumption if deemed necessary. Additionally, we will dichotomize the variable into very or extremely confident vs. not. A two-sample t-test or Wilcoxon rank sum test, as appropriate, will be used to compare the continuous outcome while a chi square test will be used to compare the dichotomized outcome. With a sample size of 50 clinicians, we will have 81% power to detect difference of 35%, e.g. from 50% to 85%, in the percentage of clinicians who are very or extremely confident in different elements needed to conduct SDM conversations. In our prior study of the SDM skills webinar, we found a 30-40% absolute increase in clinician confidence.
Hypothesis 3.2*: Clinicians in the SDM skills arm will demonstrate more SDM behaviors in the simulated interactions.* We will code each transcript using Braddock's Informed Decision Making framework that covers core aspects of SDM (scores range from 0 to 9). First, we will determine whether a two-sample t-test or a Wilcoxon rank sum test is more appropriate to compare the two groups. Assuming the score is normally distributed, a sample size of 25 in each group will have 80% power to detect a 0.81SD difference in the mean Braddock score (or about 1.4 points out of 9). Our prior study of the SDM skills webinar found an increase of 1.2SD on the Braddock scale after training, and this study is powered to detect a meaningful improvement in scores.

Heterogeneity of treatment effects (HTE)*:* The goal of the HTE analysis is to identify differential treatment effects among subgroups of patients. We will explore the HTE by examining the interaction between study arm and different factors on outcomes from both aim 1 and aim 2 analyses. These factors include (1) site (MGH vs MMC vs. PCPO), (2) gender of physician, (3) gender of patient, (4) age of patient (5) prior screening history and (6) co-morbidity burden. Linear or logistic regression models with the GEE approach will be used to test the interaction between study arms and these factors. We will also report treatment effect in each subpopulation if there are strong evidence of interactions between interventions and these factors.
Contamination and Selection Bias: We will compare screening rate from each clinician in each arm of the study to his/her own screening rate from the previous year as a gauge of potential contamination. To investigate potential selection bias, we will compare (1) clinician characteristics between those participating in the study and those who do not, (2) screening rate from the previous year from the clinicians participating the study and those who do not, and (3) patient characteristics between those participate in the study and those who are eligible but do not participate in the study. As mentioned earlier, we will compare patient characteristics between survey responders and non-responders.

D.10. Engagement Plan

D.10.a. PLANNING THE STUDY: The MGH Health Decision Sciences Center (HDSC), led by Dr. Sepucha (PI), has a standing patient advisory committee (PAC) and the PAC challenged the HDSC team to focus on training for physicians to ensure they listen to and respect patients' goals. This insight from our patient partners launched our investigation into formal skills training with a focus on training established clinicians (not students or residents). We selected decisions about continuing or stopping CRC screening for older adults based on input from patient and clinician stakeholders who identified many challenges with these discussions. The PAC members contributed to the patient cases included in the training and Ms. Kwiecien participated as a standardized patient in a prior study. The patient partners changed the emphasis from a focus on communicating about life expectancy to focusing on goals, fears and on overall health and wellbeing.

D.10.b. CONDUCTING THE STUDY: The patient partners from MGH and MMC (Ms. Kwiecien, Ms. Callahan, Mr. Kungel, Mr. Stiker) bring considerable expertise in health communication, simulated patient interactions, and patient advocacy. As evidenced in their LOS, they have contributed to the design of the proposal, and are committed to assisting in the planning, implementation and dissemination of the study. They each have personal experience with CRC screening and several also have supported spouses and friends. Other key stakeholder groups include our primary care clinicians, colorectal cancer specialists, geriatricians and population health managers. The study team and stakeholders have had weekly calls to plan the study and develop the proposal. We have planned an in-person kick off meeting to gather all patient partners, co-investigators, clinical advisors and study staff at the beginning of the project. The in-person meeting will foster strong relationships among the team, provide solid background in the science and key gaps that we intend to address, and set out roles and responsibilities to ensure successful implementation. Thereafter, the research team will have monthly calls with PIs and staff at MGH and MMC. Within each site, the research staff will have weekly meetings to review progress and milestones. **Principles for Engagement:** SDM is the focus of the study, and the principles of SDM will be used to guide the decision making and engagement of collaborators for the grant activities as well. A core concept in shared decision making is recognizing the expertise of various stakeholders, whether it is expertise in clinical care, in the lived experience of disease and treatments, or in the scientific method. Throughout the grant, Drs. Sepucha and Simmons will have ultimate financial, scientific and administrative responsibility for all grant milestones but will achieve those through open communication and shared decision making with patient, clinician and administrative partners. Two patient partners at each site have been included as key personnel and will be paid as consultants. The patient partners will participate in the monthly research team meetings and at each site, will attend some of site specific meetings. During this time, they will develop and test the standardized patient cases, and provide feedback on study recruitment materials and patient surveys to ensure they are understandable and engaging to patients. Once the study has begun, the patient partners will advise on any issues in recruitment, enrollment, or unanticipated findings. In the last year of the study, the patient partners will support interpretation of the data and presentation of the results as part of our dissemination activities. Clinician stakeholders (Atlas, Lewis, Schonberg, Richter, Percac-Lima) will attend a study kick-off meeting and have regular monthly conferences calls for the first 6 months of the grant and, thereafter, quarterly conference calls with the study team. They will also be available for 1 in-person meeting in year 1 and 3. They will ensure clinical accuracy of the webinar material and simulated patient interactions, they will also advise on the design and implementation of the registry report. The clinical stakeholders will also advise the investigators to ensure that the interventions will be able to be disseminated outside MGH and Maine.

D.10.c. DISSEMINATING THE STUDY RESULTS: Early in Year 3, we will host an in-person dissemination meeting at MGH with the entire team to create a detailed dissemination plan and specify roles and responsibilities for each of the stakeholders in implementing the plan. The patient partners have experience in communication (Callahan and Stiker) and will write up cases studies and blog posts for consumer audiences. The patient partners have also indicated willingness to be interviewed about their role and participation. MGH media services will work with the PIs and team to generate extensive coverage in popular press. We also partnered with American College of Physicians (Nickel and Erikson) to disseminate findings to their large international network of clinicians.

Research Team and Environment (Criterion 4. Investigators and Environment)

Research team: The Co-Principal Investigator, Dr. Karen Sepucha, is the Director of the Health Decision Sciences Center (HDSC) at MGH. She has a doctorate in engineering with a focus in decision sciences. Dr. Sepucha has developed valid and reliable survey instruments to measure decision quality across a dozen common medical conditions, two of which were recently endorsed as performance measures by National Quality Forum. She is actively involved in the International Patient Decision Aids Standards (IPDAS) consensus process, and leads the chapter on establishing the effectiveness of decisions. She has also been a member of the Agency for Healthcare Research and Quality (AHRQ)/National Cancer Institute (NCI) scientific evaluation group for advancing research methodology for measuring and monitoring patient-centered communication in cancer care, and has been a SDM expert for a National Quality Forum (NQF) performance measurement committee. She is currently PI on a PCORI-funded comparative effectiveness trial that has enrolled more than 1200 patients with knee and hip osteoarthritis to evaluate different decision aids.

Dr. Leigh Simmons, Co-Principal Investigator, is an internal medicine physician and the Medical Director of the HDSC at MGH. She is trained in primary care internal medicine and maintains a clinical practice at MGH. She has led the HDSC's efforts to train clinicians and office staff in shared decision-making skills, and has overseen the implementation of efforts to expand decision aid prescription at primary care practices at MGH and across Partners HealthCare. She is actively involved with programming for the Society of General Internal Medicine where she has led 3 faculty training workshops on SDM at national meetings. She recently served as an advisor to AHRQ and has presented locally, regionally, and nationally on SDM training. In addition, Dr. Simmons serves on several committees at MGH and Partners HealthCare focused on practice redesign, primary care services delivery, and patient engagement.

As described in the Leadership Plan (see **People and Places**), Drs. Sepucha and Simmons will share responsibility for ensuring timely completion of the project and have put together a team with clinical co-investigators, patient partners and consultants who have considerable expertise in key domains needed for successful completion of the project.

- **Shared decision making**: The MGH investigators (Sepucha, Simmons, Atlas, Percac-Lima), the MMC investigators (Fairfield and Han) and consultants (Schonberg and Lewis) have considerable expertise in SDM. The investigators have been involved in developing decision aids, developing measures of SDM, training clinicians in SDM using standardized patients, and implementing SDM into routine primary and specialty care for cancer screening decisions. Drs. Sepucha and Simmons oversee the distribution of approximately 10,000 patient decision aids across Partners Healthcare annually and have multiple ongoing research studies focused on measurement of SDM, comparative effectiveness of decision support strategies and clinician training on SDM skills. The HDSC also has a database of more than 1,000 patients willing to be re-contacted for future research studies.
- **Primary Care, Geriatrics and Gastroenterology**: We have engaged primary care physicians (Atlas, Simmons, Fairfield), a geriatrician (Percac-Lima), and GI specialists (Richter, Stefan) involved in counseling patients about CRC screening and delivering cancer screening. Further, one advisor (Korsen) and one co-investigator (Percac-Lima) have significant experience with community practices and cancer screening with underserved patients. The ACP (Nickel and Erickson) represents a large national and international network of primary care clinicians.
- **Patient, family and community engagement:** The patient partners bring considerable expertise in SDM (Kwiecien), health communication (Callahan), standardized patient interactions (Stiker), and research and advocacy (Kungel). As detailed in the engagement plan, these patient partners have been involved in the design of the study and development of the interventions to be examined in this proposal. Ms. Edgman-Levitan is also a nationally recognized expert in patient experience and patient engagement and will advise on dissemination. MGH has a general Patient and Family Advisory Committee (G-PFAC) that meets monthly and its co-chair, Ms. Nyeko, has invited the PIs to present the study annually and to get feedback from and engage G-PFAC members in the research.

- **Cancer registries and population health management initiatives**: Our team has strong experience conducting cluster randomized trials of primary care informatics-based systems for preventive cancer screening. Dr. Atlas (co-I) led a study that evaluated a population management informatics tool (PM IT) with 6,730 eligible women overdue for breast cancer screening, and found it increased mammography rates.[65,66] Drs. Atlas and Percac-Lima, then examined receipt of cancer screening by comparing PCP involvement to automated outreach using a PM IT tool.[67] Another study embedded patient navigation (PN) with a PM IT tool with low income and minority patients at high risk for non-adherence. The randomized trial of 1,613 patients found that intervention patients were more likely to complete cancer screening.[68] These studies demonstrate our ability to implement large, randomized studies of preventive cancer screening using system-wide, IT-enhanced patient- and clinician-level interventions.

Environment: MGH has nineteen affiliated primary care practices that care for adults, including 12 community-based practices and 3 hospital-based practices serving a diverse patient population in Eastern Massachusetts. The practices are staffed by more than 250 internal medicine physicians, physician assistants and nurse practitioners. The practices care for about 170,000 patients who have more than 1,000,000 patient visits each year. To increase diversity of our study patient population, we will target enrolling clinicians from our four health centers. **MGH Everett Family Care** works hard to ensure that the services provided reflect the needs of their diverse community. The racial makeup of the city is 53.6% Non-Hispanic White, 14.3% African American, 4.8% Asian, 2.4% from other races, and 3.8% were multiracial. Hispanic or Latino of any race were 21.1% of the population. About 11.9% of the population is below the poverty line, including 10.0% of those 65 or older. **MGH Chelsea HealthCare Center**- serves a diverse, working-class community that contains a high level of industrial activity. It is about 47.8% White, 8.5% African American or Black, and 37.9% other races. In addition, 62.1% of residents identify as Hispanic or Latino. About 20.6% of families and 23.3% of the population were below the poverty line, including 20.9% of those 65 or older. **MGH Revere HealthCare Center**, which opened in 1981 is a valued community resource and in addition to adult and pediatric primary care, offers many medical specialties on site. The racial makeup of the city was 62.4% White, 4.9% Black, 5.6% Asian, 12.1% from other races, and 3.3% were multiracial. Hispanic or Latino persons were 24.4% of the population. About 14.6% of the population is below the poverty line, including 10.4% of those 65 or over. **MGH Charlestown HealthCare Center**- Charlestown is the oldest neighborhood in Boston, MA. The population of Charlestown is non Hispanic white (75.4%), Black or African (7.4%), Asian (7.5%), Hispanic or Latino (7.4%), and multiracial (2.2%). About 17% percent of the population live below the poverty line. Human Subjects section on Planned Enrollment includes detailed estimates for patient race and ethnicity.

The Partners Community Physicians Organization (PCPO) has more than twenty years of experience working with Partners' community providers, and offers both practice management services and population health implementation support to its employed and affiliated physician groups. PCPO currently supports more than 6,000 community physicians in efforts to improve the quality and efficiency of health care services. We will enroll clinicians from two PCPO primary care systems with 16 practices and 128 clinicians. Key strengths of including clinicians from the PCPO include increased generalizability to nonacademic practices and administrative and system resources (e.g. reciprocating IRB, shared EHR) to support successful study implementation.

MMC is a not-for-profit family of leading high-quality providers and other healthcare organizations working together so their communities are the healthiest in America. MMC was the recipient of an implementation grant from the Informed Medical Decisions Foundation from 2009-2013.The goal of that project was to develop infrastructure, including physician and practice team education, and deploy tools for SDM across primary care settings at MMC. Dr. Fairfield was Co-PI for the project. Through that work, Dr. Fairfield worked with providers from around the MMC to educate clinical teams about SDM using decision aids, and to optimize office workflows for decision aid dissemination. The experience of the investigators (Han and Fairfield) in SDM and risk communication including use of standardized patients to assess skills, the strong relationships between the PIs and the MMC team, and the geographic diversity of patient population made the site a strong collaborator for this project.

DISSEMINATION AND IMPLEMENTATION POTENTIAL

For detailed instructions, refer to the Application Guidelines for your PFA. Do not exceed one page.

Both interventions will be able to be disseminated widely. We designed the SDM skills training intervention on an online platform to make it easy for clinicians to access and use. Similarly, the telephone-based simulated patient interactions also provide flexibility in scheduling. The registry intervention was selected because of its widespread use in primary care practices to support cancer screening in younger patients, and the ease of extending it to older patients. The American College of Physicians (Nickel and Erickson), the largest professional society for internal medicine clinicians, has signed on as a stakeholder because of their great interest in the topic and desire to disseminate the findings widely.

There is also a large potential to disseminate and implement the findings locally. MGH is part of Partners Healthcare System (PHS), and the PHS Center for Population Health coordinates primary and specialty care initiatives across its network of more than 8 hospitals and 300 primary care practices with thousands of clinicians. Similarly, MMC is part of MaineHealth, a large integrated delivery system with more than 16 member organizations. Both organizations have institutional commitment to SDM and routinely promote CME training for clinicians across the network and would be willing to promote SDM skills training (see LOS from Chaguturu and St. Germain). The team will secure slots to present at MMC and MGH grand rounds, department and faculty meetings, and will place articles in hospital- and system-wide newsletters to reach a broad clinical audience. The results will also be disseminated nationally at key scientific and professional meetings such as SGIM, and SMDM as well as publications in leading journals to reach the clinician and research community more broadly.

The team will work with MGH, MMC and Partners' public affairs departments to generate extensive coverage in the popular press. The PIs and co-investigators have had their work covered by NPR, the Boston Globe, the New York Times, as well as Harvard Men's Health and Harvard's Women's Health publications and plan to develop media kits that will facilitate coverage and reach large consumer audiences. Patient partners will play key role in supporting media dissemination, including co-writing case studies, being interviewed, co-writing key findings.

A. Describe how you will make study results available to study participants after you complete your analyses.

Routine updates on the study progress will be posted on the HDSC website (https://mghdecisionsciences.org/) at least every six months. At the end of the study, public information about the study findings and links to published abstracts or open access articles will be posted on the HDSC website. Study participants will be able to sign up for the HDSC quarterly newsletter that will contain study updates and will be encouraged to check the HDSC website. We will also include articles summarizing the results of the study in patient-facing communications at each site to reach the broader community.

B. Describe possible barriers to disseminating and implementing the results of this research in other settings.

Lack of time: The PIs and team will work to ensure the webinar and associated training components are as streamlined and convenient as possible (e.g. online webinar, telephone-based simulated patient interactions with flexible scheduling) to fit into busy schedules. Further, they will work with stakeholders from the American College of Physicians, a leading CME provider, to build on their expertise in large scale dissemination of educational trainings.

Lack of funds: After the study, the webinar will be freely available, but there will be a cost for clinicians who wish to receive CME credits and/or to have telephone-based SPIs. The CME cost will be within current market rate $30-50 per hour of CME. We will explore other sources for covering the cost of the SPIs, but will not solicit funds from pharmaceutical or other for-profit companies that may have an interest in CRC treatment or testing options.

PROTECTION OF HUMAN SUBJECTS

The proposed study is considered Human Subjects Research and meets the definition of a clinical trial. This study is categorized as an NIH-defined Phase IV trial comparing effectiveness of established interventions, namely decision support strategies. As a result, this human subjects section follows the requirements in "Scenario E" from Section 5.0 "Human Subjects Research Policy" referenced in the instructions above.

1. Protection of human subjects

1.1 Risks to human subjects

a. ***Human subjects involvement, characteristics, and design***

We propose to conduct a cluster randomized control trial to examine the effectiveness of two established decision support strategies for decisions about stopping colorectal cancer screening for adults between the ages of 76-85. In the study, primary care clinicians will be recruited from Massachusetts General Hospital (MGH), Maine Medical Center (MMC), and Partners Community Physicians Organization (PCPO). Clinicians will be randomized into two groups stratified by gender and years of experience. Then, each group will be randomly assigned to one of two arms: Registry arm or SDM Skills training. In the Registry only arm, clinicians will receive a report of their patients aged 76-85 with an upcoming visit and their colorectal cancer screening status. In the SDM Skills arm, clinicians will receive the report and participate in a SDM skills training including a webinar and standardized patient interaction with feedback. All participating clinicians will complete one standardized patient assessment and a baseline survey before enrolling any patients on the trial. Once patient enrollment begins, research staff will survey eligible patients about one week after the visit with the participating clinician to examine the impact of the interventions on patients' knowledge, preferences for screening and reports of SDM in the visit. A short follow up survey of patients at 12 months will confirm screening choice and identify any reasons the preferred choice was not implemented.

Sample: We plan to enroll 30-35 clinicians from MGH, 10-15 from MMC, and 10-15 from PCPO. Eligible clinicians will be licensed primary care physicians, nurse practitioners, or physician assistants, who have completed their training, are in clinical practice >20% time or have at least 30 eligible patients in their panel. Trainees (residents and medical students) will be excluded. We plan to recruit clinicians within these sites who have a large panel of patients aged 76-85, and who have diverse patient panels. We estimate that if we enroll 60 clinicians then we will have 50 clinicians complete the baseline assessments and be randomized to a study arm. The eligibility criteria for patient participants are listed in Table 1 and are the same across sites. All patients between the ages of 76-85 coming in for a non-urgent care visit will be screened for eligibility.

Table 1: Eligibility for patient participants

	Eligible	Ineligible
Colorectal Cancer Screening	• Adults, age 76-85<br>• Scheduled for non-urgent office visit with a participating clinician during the study period<br>• Due for colorectal cancer screening in upcoming year or never been screened	• Prior diagnosis of colon or rectal cancer, inflammatory bowel disease or genetic disorder that raises CRC risk (hereditary non-polyposis CRC and familial adenomatous polyposis)<br>• Unable to consent for themselves<br>• Unable to read or write in English or Spanish

We plan to enroll 60 clinicians and estimate that 15% will be lost to follow up (e.g. move, drop out) and 50 will complete the study. We plan to approach about 17 patients per provider, or 830 patients overall and estimate that about 60% will complete the post-visit survey. This will result in 500 completed surveys, or about 250 per arm. We estimate that we will need to screen about 1200 patients to find 830 eligible ones.

Recruitment: Investigators at each site will target clinicians with a high volume of patients aged 76-85 and those who see patients at community health centers or centers with higher patient diversity. Those clinicians will be targeted for the first round of invitations. Letters from medical director, network director and PI will invite clinicians to participate. Follow up calls and emails will be sent to encourage participation. We expect that if we invite 120 clinicians across the three networks, 60 (50%) will agree to join the study, and 50 of those will complete the study. Participating clinicians at each site (MGH, MMC, PCPO) will be split into two groups that are balanced for gender and years of experience. Then, each group will be randomized to an arm. Those clinicians randomized to the SDM Skills arm will be sent an email by study staff detailing the training procedures. Clinicians in the SDM arm will have four weeks to complete the baseline survey, webinar, and two telephone-based SP interviews. Clinicians in the Registry arm will have four weeks to complete the baseline survey and one telephone-based SPI. Study staff will send reminders to ensure completion the activities. The PI and clinical co-investigators will also send reminder emails and calls to encourage completion of the clinician interventions and assessments as needed.

Once the registry has started, staff will review medical record to identify eligible patients from the participating clinicians prior to their scheduled visit. All eligible patient participants will receive a cover letter signed by their clinician inviting their participation and an information sheet describing the study. The cover letter will include information for participants to opt-out if they desire. All potentially eligible patients will be sent a survey about one week after their visit with the participating clinician. Patient consent for the study will be implied by completion of the survey.

b. ***Sources of materials***

The purpose of the study is to evaluate the comparative effectiveness of decision support strategies and examine their ability to promote SDM about colorectal cancer screening for older adults. Clinician participation in this study involves reviewing reports, competing the training (if assigned), one survey, a simulated patient interaction, and a short debrief interview. There are no foreseeable safety risks to clinician participants for participating. Patient participation in this study involves the completion of a survey shortly after their visit and again about one year later. It also involves collecting information about screening history, screening use and overall health from the medical record.

Study staff will be instructed to review the surveys within a week of receipt and will notify the PI and clinical investigators on the team about any serious events immediately and all other events at regularly scheduled meetings. Study staff will keep records of any feedback, questions, concerns and/or complaints that are received and these will be address as needed.

The main safety risk for both clinician and patient participants involves privacy. Participant names will not be included in any paper survey instruments and will only be referenced by study code number. The file that links the study code numbers to names and contact information will be kept separately on a Partners password protected server. Survey data will be collected using Research Electronic Data Capture (REDCap). REDCap is a free, secure, HIPAA-compliant web-based application hosted by the Partners HealthCare Research Computing, Enterprise Research Infrastructure & Services (ERIS) group. The system offers easy data manipulation with audit trails, reports for monitoring and querying participant records, and an automated export mechanism to common statistical packages.

c. ***Potential risks***

There are minimal risks to participating individuals associated with or attributable to this study. The main risks are associated with loss of privacy of their health information. The time required for clinician participants is about 2 hours for the Registry only arm and about 4 hours for the SDM Skills arm spread out over the course of about a year. Those clinicians participating in the SDM Skills training interventions will do so during their administrative time. The time required for patient participants to complete the surveys is about 15-20 minutes for the first survey and 10 minutes for the second. Consent for patient participants will be implied by completion of the survey. Patient participants may opt out of the study, may refuse to answer any question (or set of questions) and may discontinue their participation at any time. It will also be emphasized that whether subjects participate will not impact the medical care that they receive.

1.2 Adequacy of protection against risks

a. ***Recruitment and informed consent***

Subject recruitment will follow best practices for human subjects research and will occur as follows:

1. PI and co-investigators will present at practice team meetings, send individual invitation emails, and otherwise advertise the opening of the study and encourage interested clinicians to contact a member of the study team for more information.
2. Clinicians who indicate interest will be screened to confirm eligibility (mainly confirming adequate number of eligible patients in their panel) and will then be sent a research information sheet describing the study. Clinicians will indicate their consent by responding to the email with their intention to join the study.
3. Clinicians will be given a link to an online baseline survey. Study staff will follow up with email and phone reminders to encourage response.
4. The participating clinicians will be assigned to the Registry only arm or SDM Skills arm according to the pre-specified randomization scheme.
5. Clinicians randomized to the SDM Skills arm will be scheduled to complete one SP, then the webinar, then the second SP. Clinicians randomized to the registry arm will complete one SP. Clinicians will have 1 month to complete these activities.
6. The research coordinators will review the medical charts of patients with scheduled appointments and complete the eligibility screener and prepare the report from the registry.
7. About one week before the visit, the research coordinator will send a cover letter signed by the participating clinician and an information sheet describing the study to all eligible patients. The cover letter will have information for participants who wish to opt out of the study.
8. Depending on clinician's preferences, clinicians will receive a report listing patients with upcoming visits and screening status either daily or weekly.
9. All patient participants who do not opt out will receive the study survey by mail with a $5 incentive about one week after the visit. Study staff will follow up with up to three phone reminders and one mailed reminder for all the participants who did not return the survey.
10. At 12 months, staff will call all patient participants for short follow-up to determine any barriers to implementing preferred choice.

The research coordinator will track the number of participants screened, reason for ineligibility, the number sent invitation by mail, the number who opted out or otherwise declined participation and any reasons given for the refusal to participate for reporting in CONSORT flow diagram. There are no formal written consent procedures in this study. The research presents no more than minimal risk of harm to subjects and involves no procedures for which written consent is normally required. Consent for the study will be implied by completion of the one survey for patient participants and email consent for clinician participants.

All study staff are Collaborative Institutional Training Initiative (CITI) certified and will receive training from the PI and program manager in the study protocol.

b. ***Protections against risk***

There are minimal risks to individuals participating in this project. The main risks are the time and effort involved in participating and loss of privacy. There are no known risks or safety concerns for those clinicians who participate in the SDM Skills sessions including the SPI. However, study staff will monitor feedback obtained and any conversations that may occur in which participants and SPs express complaints or problems related to the study. All subjects will be told that participating in the study and completing the survey is voluntary and that they may refuse to answer any questions or set of questions and may discontinue their participation at any time.

To address privacy and confidentiality issues, the paper surveys will be identified by code number only. Online surveys will be programmed into the REDCap web-based survey. All patient identifiers will be kept in a password protected files

on password protected PHS servers for the MGH team and MMC servers for the Maine team. Files shared between sites will be de-identified and will use the Partners secure file transfer website (not email) to send and receive study files. Only members of the research team will have passwords to access the folders and files and they will only access the files from PSH or MMC computers or encrypted laptops that are protected with SafeBoot. To address issues of psychological discomfort, study materials will inform patients that they may refuse to answer any question and may withdraw from the study at any time. Surveys and reminders will also include contact information for the PI, Co-investigators and study staff in case participants have questions or concerns. In similar survey studies that we have run, we have only had calls from participants for clarification of questions or logistical issue, none related to harms related to their participation.

1.3 Potential benefits of the proposed research to human subjects and others

SDM in the clinic visit has been shown to increase patient satisfaction and increase match between patients' preferences and their treatment choices. Those clinicians randomized to the SDM Skills arm may benefit as prior work has shown the training results in increased confidence and competence in conducting SDM conversations with patients. The report that all clinicians will receive may prompt them to discuss cancer screening with their older population of patients.

There are no direct benefits to patients from completing the surveys. The potential benefit to society is that the study will help determine the most effective approach to engaging and informing older patients about cancer screening.

1.4 Importance of the knowledge to be gained

An older population of patients facing a significant medical decision, such as the decision to continue cancer screening or not for colorectal cancer, need help to ensure that their decisions are well informed and reflect their goals. Poor quality decisions are problematic for patients, clinicians and health care systems. SDM is a skill that supports clinicians and their patients to have a well-informed discussion, including the potential benefits and harms, to more effectively communicate complex medical information to patients and families. As efforts to integrate SDM into routine care expand, understanding the comparative effectiveness of different interventions is critical. This study will provide important new information on comparative effectiveness of different decision support strategies promoting SDM.

1.5 Data and safety monitoring plan (DSMP)

Oversight of the trial is provided by the Co-PIs, Drs. Simmons and Sepucha at MGH and co-investigators, Drs. Han and Fairfield at MMC. The trial is minimal risk and the DSMP is commensurate with the potential risk level.

Monitoring procedures: Although there are no written informed consent forms, Drs. Simmons and Sepucha are responsible for assuring that clinician and patient participants are adequately informed prior to engaging in any research procedures, that all subjects meet eligibility criteria, and that the study is conducted according to the IRB-approved research plan. Study data will be accessible at all times for the Co-PIs to review. The Co-PIs will examine study conduct including enrollment, accrual, drop-outs, and protocol deviations on a weekly or every other week basis with the staff at each site. Study staff will review study related data including comments from the SPIs, reminder phone calls to participants, participant surveys and will notify the PI about any serious or moderate potential adverse events (AEs) immediately and any minor or potential ones at regular meetings. The Co-PIs will review AEs individually real-time and in aggregate on a regular basis at team meetings. The Co-PIs and co-investigators will review potentially serious adverse events (SAEs), as soon as they are discovered. The Co-PIs will ensure all protocol deviations, AEs, and SAEs are reported to the IRB according to the standard requirements.

Collection and reporting of SAEs AND AEs: AEs are identified by study staff through interactions with study participants and upon review of completed surveys. SAEs and specific study-associated AEs are reported to the PI and IRB within 24 hours. In addition, all AEs are reported according to the Partners Human Subjects Committee AE reporting guidelines.

Adverse event monitoring and reporting: No SAEs are expected based on the minimal risk in the trial. However, if a SAE occurs then the PI will report the event to the IRB within 24 hours and will file an HRC AE Form within 10 working days. If a mild or moderate AE occurs, the PI will summarize the event in the progress report at the annual continuing review.

Based on prior experience fielding survey research, the most common issue that arises is a clinical question or request for which the study staff are not qualified to answer or complete (e.g. regarding rescheduling of visits, or ordering tests).

The PI and Co-PIs will train staff in the appropriate way to handle clinical issues and will work with participating clinicians and their staff to ensure communication for any issues that arise.

Data analysis plans: The analysis of study data will occur after all the patient survey data has been collected. The screening rates will be calculated at the clinician, practice and network level according to established, validated algorithms currently in use at each network. The analysis will also summarize all AEs, and compare across each arm of the trial. Given the minimal risk of this trial there are no planned stopping rules.

Plan for data management: Compliance of regulatory documents and study data accuracy and completeness will be maintained through an internal study team quality assurance process. Patient confidentiality will be maintained as is routine for all patient care privacy guidelines. Special efforts will be made to protect the privacy of subjects. We will have names, addresses, phone numbers and email addresses of eligible participants and this information will be kept separate from the study data and analysis files. Surveys and messages sent by email will be encrypted using the “send secure” institutional policy. The study code number will be used to help identify participants who need to be followed up for non-response. A separate password-protected electronic file will contain the codes linked to identifying information. Only the IRB approved study staff and investigators will have access to this file. These will be kept as long as required by the research project. After the study has been completed, all study files containing personal health information of participants will be destroyed.

1.6 ClinicalTrials.gov

The study will be registered in ClinicalTrials.gov before the first subject is enrolled. The reporting of summary results information (including adverse events) will be completed according to the requirements and no later than 1 year after the completion date.

2. Inclusion of women and minorities

All eligible patient participants who have a scheduled appointment at a participating site will be included, and we expect the demographics will reflect those of the underlying patient panels. We plan to target clinicians at community health centers affiliated with MGH to increase racial and ethnic diversity. Both men and women will be included in the study and study materials will be available in English and Spanish. Most patients seen at these sites speak either English or Spanish (>97%), and patients with other primary language will be excluded.

3. Planned enrollment report for the 500 patients to be enrolled on the trial

Race	Male (N)	Female (N)	Total (N)
American Indian/Alaska Native	0	0	0
Asian	9	11	21
Black/African American	17	21	37
Hawaiian/Pacific Islander	0	0	0
White	194	235	429
Multirace	7	6	12
**Ethnicity**	**Male (N)**	**Female (N)**	**Total (N)**
Hispanic (Latino/Latina)	17	33	50
Non-Hispanic	209	240	450

4. Inclusion of children Children will not be eligible for this study. The research topic to be studied (continuing colorectal cancer screening for adults 76-85) is not relevant to children.

CONSORTIUM CONTRACTUAL ARRANGEMENTS

For detailed instructions, refer to the Application Guidelines for your PFA. Do not exceed five pages.

Describe the proposed research projects that subcontracted organizations will perform. Explain the strengths that these partners bring to the overall project to ensure successful submission of contract deliverables in accordance with the milestone schedule.

There is one subcontract with this proposal, between MGH and Maine Medical Center (MMC). The MMC subcontract paperwork includes the financial and administrative details for the arrangement between MGH and MMC. In the People and Places template there is a detailed description of the MMC site and the Research team and Environment section summarizes the key strengths of the site. MMC was chosen as a site for this study because of the strong existing relationship between the MGH PIs (Sepucha and Simmons) and the MMC PIs (Han and Fairfield), and because of the strong commitment to and experience with shared decision making, primary care, and medical education. Further, MMC provides access to a predominantly rural community compared to the predominantly urban community served by MGH. Having multiple sites for recruitment will strengthen the study by providing important evidence of the generalizability of the approach and helping to meet the recruitment milestones in the short study time frame.

Partners' Community Physicians Organization is also participating as a recruitment site; however, we will not need a formal subcontract with the PCPO. Both the PCPO and MGH are part of Partners HealthCare, and the community practices targeted are all using Partners eCare electronic health record; as a result, all study activities can be managed centrally through the MGH staff. We have selected PCPO clinicians at these community practices because of the support of leadership, generalizability to nonacademic sites, and the efficiency to be able to conduct the study centrally as opposed to having to hire additional coordinators at each community practice.

REFERENCES CITED

For detailed instructions, refer to the Application Guidelines for the PFA. Do not exceed 10 pages.

[1] US Preventive Services Task Force. Screening for colorectal cancer US Preventive Services Task Force Recommendation Statement. The Journal of the American Medical Association 2016;315(23):2564-2575.

[2] Surveillance, Epidemiology, and End Results Statistics Fact Sheets: Colon and Rectum Cancer. National Cancer Institute. Bethesda, MD. https://seer.cancer.gov/statfacts/html/colorect.html Accessed December 8, 2016.

[3] Colorectal cancer facts and figure 2017-2019. American Cancer Society. https://www.cancer.org/content/dam/cancer-org/research/cancer-facts-and-statistics/colorectal-cancer-facts-and-figures/colorectal-cancer-facts-and-figures-2017-2019.pdf. Published 2017. Accessed January 26, 2018.

[4] Klabunde CN, Shapiro JA, Kobrin S, Nadel MR, Zapka JM. Colorectal cancer screening in U.S. seniors ages 76-84 years. *Journal of Community Health*. 2015;40(4):769-779. doi:10.1007/s10900-015-9998-z.

[5] Klabunde CN, Joseph DA, King JB, et al. Vital signs: colorectal cancer screening test use: United States, 2012. MMWR Morb Mortal Wkly, Centers for Disease Control and Prevention (CDC). Rep. 2013;62(44):881-888.

[6] Knudsen AB, Zauber AG, Rutter CM, et al. Estimation of Benefits, Burden, and Harms of Colorectal Cancer Screening Strategies: Modeling Study for the US Preventive Services Task Force. *JAMA*. 2016;315: 2595-2609.

[7] Lin JS, Piper MA, Perdue LA, et al. Screening for colorectal cancer: updated evidence report and systematic review for the US Preventive Services Task Force. JAMA. 2016. 315: 2576-2594.

[8] 2014 National Population Projections: Table 9. Projections of the Population by Sex and Age for the United States: 2015 to 2060. U.S. Census Bureau, Population Division. 2014.

[9] Welch HG. The problem with free healthcare. *The New York Times*. April 30, 2014. https://www.nytimes.com/2014/05/01/opinion/the-problem-with-free-health-care.html?_r=0. Accessed December 15, 2016.

[10] Wilt TJ, Harris RP, Qaseem A. Screening for cancer: advice for high-value care from the American College of Physicians. *Ann Intern Med*. 2015;162:718-725.

[11] Wolfson D, Santa J, Slass L. Engaging physicians and consumers in conversations about treatment overuse and waste: a short history of the Choosing Wisely campaign. *Acad Med*. 2014;89:990-995.

[12] Choosing Wisely. http://www.choosingwisely.org/doctor-patient-lists/. Accessed December 8, 2016.

[13] Lansdorp-Vogelaar, I, Gulati R, Mariotto AG. Personalizing age of cancer screening cessation based on comorbidity: model estimates of harms and benefits. *Ann Intern Med*. 2014;161:104-112.

[14] Coulter A, Cleary PD. Patients' experiences with hospital care in five countries. *Health Affairs* (Millwood). 2001; 20(3):244-252.

[15] Fagerlin A, Sepucha K, Couper M, et al. Patients' knowledge about 9 common health conditions: Data from a national representative sample. *Medical Decision Making*. 2010;30(5 Suppl): 35S-52S.

[16] Zikmund-Fisher BJ, Couper MP, Singer E, et al. Deficits and variations in patients' experience with making 9 common medical decisions: the DECISIONS survey. *Medical Decision Making*. 2010;30(5 Suppl):85S-95S.

[17] Lewis CL, Kistler CE, Amick HR, et al. Older adults' attitudes about continuing cancer screening later in life: a pilot study interviewing residents of two continuing care communities. BMC Geriatrics. 2006;6:10.

[18] Initial Preventive Physical Examination (IPPE). https://www.cms.gov/Outreach-and-Education/Medicare-Learning-Network-MLN/MLNProducts/Downloads/MPS_QRI_IPPE001a.pdf. Accessed December 8, 2016.

[19] Medicare Annual Wellness Visit (AWV): https://www.cms.gov/Outreach-and-Education/Medicare-Learning-Network-MLN/MLNProducts/downloads/AWV_chart_ICN905706.pdf Accessed December 8, 2016.

[20] Yabroff KR, Klabunde CN, Yuan G, et al. Are physicians' recommendations for colorectal cancer screening guideline-consistent? *J Gen Intern Med.* 2011;26:177–184.
[21] Zapka JM, Klabunde CN, Arora NK, Yuan G, Smith JL, Kobrin SC. Physicians' colorectal cancer screening discussion and recommendation patterns. *Cancer Epidemiol Biomarkers Prev*. 2011;20:509–521.
[22] Zapka J, Klabunde CN, Taplin S, Yuan G, Ransohoff, Kobrin S. Screening colonoscopy in the U. S.: attitudes and practices of primary care physicians. *J Gen Intern Med*. 2012;9:1150–1158.
[23] Yabroff KR, Zapka J, Klabunde CN, et al. Systems strategies for support cancer screening in U.S. primary care practice. *Cancer Epidemiol Biomarkers Prev*. 2011;20:2471–2479.
[24] Inadomi JM, Vijan S, Janz NK, et al. Adherence to colorectal cancer screening: a randomized clinical trial of competing strategies. *Arch Intern Med*. 2012;172: 575-582.
[25] Lillie SE, Partin MR, Rice K, Fabbrini AE, Greer NL, Patel SS, MacDonald R, Rutks I, Wilt TJ. The effects of shared decision making on cancer screening – a systematic review. Washington (DC): Department of Veterans Affairs (US); 2014 Sep. Available from http://www.ncbi.nlm.nih.gov/books/NBK310872/. PubMed PMID: 26290937.
[26] Sarfaty M, Wender R, Smith R. Promoting cancer screening within the patient centered medical home. *CA: A Cancer Journal for Clinicians*. 2011;61(6):397-408.
[27] Stone EG, Morton SC, Hulscher ME, et al. Interventions that increase use of adult immunization and cancer screening services:a meta-analysis. *Ann Intern Med.* 2002;136:641-651.
[28]Lafata EJ, Brown RF, Pignone MP, Ratliff S, Shay LA. Primary care physicians' support of shared decision making for different cancer screening decisions.*Med Decis Making.* 2017; 37(1):70-78.
[29] Schoenborn NL, Lee K, Pollack CE, Armacost K, Dy SM, Bridges JFP, Xue QL, Wolff AC, Boyd C. Older Adults' Views and Communication Preferences About Cancer Screening Cessation. JAMA Intern Med. 2017 Aug 1;177(8):1121-1128. doi: 10.1001/jamainternmed.2017.1778. PubMed PMID: 28604917; PubMed Central PMCID: PMC5564296.
[30] Campbell MK, Piaggio G, Elbourne DR, Altman DG; for the CONSORT Group. Consort 2010 statement: extension to cluster randomised trials. BMJ. 2012 Sep 4;345:e5661. PMID: 22951546.
[31] Montgomery AA, Schulz K, Altman DG, Moher D. CONSORT 2010 Statement: Updated guidelines for report parallel group randomized trials. *BMJ.* 2010;340:c332.
[32] Sepucha KR, Abhyankar P, Hoffman AS et al. Standards for Universal reporting of patient Decision Aid Evaluation studies: the development of SUNDAE Checklist. *BMJ Qual Saf.* Published Online First: 21 December 2017. doi: 10.1136/bmjqs-2017-006986
[33] Mulley AG. Methodological issues in the application of effectiveness and outcomes research to clinical practice. Effectiveness and Outcomes in Health Care Washington D.C.: National Academy Press; 1990.
[34] Sepucha K, Mulley A. A Perspective on the Patient's Role in Treatment Decisions. *Medical care research and review*. 2009;Feb;66(1 Suppl):53S-74S.
[35] Sepucha K, Mulley A. Extending Decision Support: Preparation and Implementation. Patient education and counseling. 2003;50(3):269-71.
[36] Charles C, Gafni A, Whelan T. Decision-making in the physician–patient encounter: revisiting the shared treatment decision-making model. *Social science & medicine*. 1999;49(5):651-661.
[37] Makoul G and Clayman ML. An integrative model of shared decision making in medical encounters. *Patient education and counseling*. 2006;60:301-312.
[38] Légaré F, Moumjid-Ferdjaoui N, Drolet R, et al. Core competencies for shared decision making training programs: insights from an international, interdisciplinary working group. *The Journal of continuing education in the health professions*. 2013;33(4):267.
[39] Wagner EH, Austin BT, Von Korff M. Improving outcomes in chronic illness. *Managed care quarterly*. 1996;4(2):12-25.
[40] Wagner EH, Austin BT, Von Korff M. Organizing care for patients with chronic illness. *Milbank quarterly.* 1996;74(4):511-544.

[41] Zapka JG, Taplin SH, Solberg LI, Manos MM. A framework for improving the quality of cancer care: the case of breast and cervical cancer screening. *Cancer Epidemiol Biomarkers Prev.* 2003;12(1):4-13.
[42] Zapka J, Taplin SH, Ganz P, Grunfeld E, Sterba K. Multilevel factors affecting quality: examples from the cancer care continuum. *J Nat Cancer Inst Monographs.* 2012;2012(44):11-19.
[43] Taplin SH, Anhang Price R, Edwards HM, et al. Introduction: Understanding and influencing multilevel factors across the cancer care continuum. *J Nat Cancer Inst Monographs.* 2012;2012(44):2-10.
[44] Simmons L, Leavitt L, Ray A, et al. Shared decision making in common chronic conditions: Impact of a resident training workshop. *Teaching and learning in medicine*. 2016;28(2):202-209.
[45] Sepucha K, Simmons L, Barry M, et al. Ten years, forty decision aids, and thousands of patient uses: Shared decision making at Massachusetts General Hospital. *Health Affairs*. 2016;35:630-636.
[46] Sepucha K, Simmons LH, Meyer C, Leavitt L, Marques F. A randomized pilot study examining approaches to training clinicians to conduct shared decision making conversations. Society for Medical Decision Making's 39th Annual North American Meeting, 2017 (oral abstract).
[47] Han P, Joekes K, Elwyn G, et al. Development and evaluation of a risk communication curriculum for medical students. *Patient education and counseling*. 2014;94:43-49.
[48] Han P, Piccirillo J, Gutheil C, et al. Development and Evaluation of an online risk communication teaching program for medical students. *Medical Science Educator*. 2016;26(4):557-567.
[49] Davis D. Does CME work? An analysis of the effect of educational activities on physician performance or health care outcomes. *The International Journal of Psychiatry in Medicine*. 1998;28(1):21-39.
[50] Dillman D. Mail and Telephone Surveys: The Total Design Method. New York: John Wiley; 1978.
[51] Edwards PJ, Roberts I, Clarke MJ, DiGuiseppi C, Wentz R, Kwan I, Cooper R, Felix LM, Pratap S. Methods to increase response to postal and electronic questionnaires. Cochrane Database of Systematic Reviews 2009(3):Art. No.: MR000008.-DOI: 10.1002/14651858.MR000008.pub4
[52] Sepucha K, Atlas S, Chang Y, et al. Patient decision aids improve decision quality, patient experience and reduce surgical rates in routine orthopaedic care: prospective cohort study. *Journal of Bone and Joint Surgery*. 2017; 2;99(15):1253-1260.
[53] Hoffman T, Lewis CL, Pignone MP, et al. Decision-making processes for breast, colorectal, and prostate cancer screening: the DECISIONS survey. *Medical Decision Making*. 2010;30(5 Suppl):53S-64S.
[54] Sepucha K, Stringfellow V, Fowler FJ. Shared Decision Making (SDM) Process Survey: Validity and Reliability of a Short, Patient-Reported Measure of SDM. 2017 Society for Medical Decision Making Annual Meeting abstract.
[55] Sepucha K, Feibelmann S, Cosenza C, Levin CA, Pignone M. Development and evaluation of a new survey instrument to measure the quality of colorectal cancer screening decisions. *BMC medical informatics and decision making*. 2014;14(1):72.
[56] Hoffman RM, Elmore JG, Pignone MP, Gerstein BS, Levin CA, Fairfield KM. Knowledge and values for cancer screening decisions: Results from a national survey. Patient Educ Couns. 2016 Apr;99(4):624-30.
[57] Health Information National Trends Survey. https://hints.cancer.gov/ Accessed December 5, 2016.
[58] DeSalvo KB, Bloser N, Reynolds K, He J, Muntner P. Mortality Prediction with a Single General Self-Rated Health Question: A Meta-Analysis. *Journal of General Internal Medicine*. 2006;21(3):267-275. doi:10.1111/j.1525-1497.2005.00291.x.
[59] Morris NS, MacLean, CD, Chew LD, Littenberg B. The Single Item Literacy Screener: evaluation of a brief instrument to identify limited reading ability. *BMC Family Practice*. 2006;7(1):1.
[60] Brice JH, Foster MB, Principe S, Moss C, Shofer FS, Falk RJ, DeWalt DA. Single-item or two-item literacy screener to predict the S-TOFHLA among adult hemodialysis patients. *Patient education and counseling*. 2014;94(1):71-75.
[61] Leader A, Daskalakis C, Braddock C, Kunkel EJ, et al. Measuring Informed Decision Making about Prostate Cancer Screening in Primary Care. Med Dec Making. 2011; 32(2):327-36.

[62] Price EL, Bereknyei S, Kuby A, Levinson W, Braddock CH. New elements for informed decision making: a qualitative study of older adults' views. Patient education and counseling. 2012;86(3):335-341.
[63] Liang KY and Zeger S. Longitudinal data analysis using generalized linear models. Biometrika. 1986;73(1):13–22.
[64] Stacey D, Légaré F, Lewis K, Barry MJ et al. Decision aids for people facing health treatment or screening decisions. *Cochrane Database of Systematic Reviews* 2017, Issue 4. Art. No.: CD001431. DOI: 10.1002/14651858.CD001431.pub5
[65] Atlas SJ, Grant RW, Lester WT, et al. A cluster-randomized trial of a primary care informatics-based system for breast cancer screening. Journal of general internal medicine. 2011;26(2):154-161.
[66] Atlas SJ, Ashburner JM, Chang Y, Lester WT, Barry MJ, Grant RW. Population-based breast cancer screening in a primary care network. The American journal of managed care. 2012;18(12):821-829.
[67] Atlas SJ, Zai AH, Ashburner JM, et al. Non-visit-based cancer screening using a novel population management system. The Journal of the American Board of Family Medicine. 2014;27(4):474-485.
[68] Percac-Lima S, Ashburner JM, Zai AH, et al. Patient Navigation for Comprehensive Cancer Screening in High-Risk Patients Using a Population-Based Health Information Technology System: A Randomized Clinical Trial. *JAMA intern medicine.* 2016;176(7):930-937.

APPENDIX (optional)

For detailed instructions, refer to the Application Guidelines for the PFA. Do not exceed 10 pages.

Appendix A: Sample registry report generated for primary care clinician with existing electronic health record functionality (personal health identifiers redacted). Study staff will be able to easily identify participating clinicians potentially eligible patients.

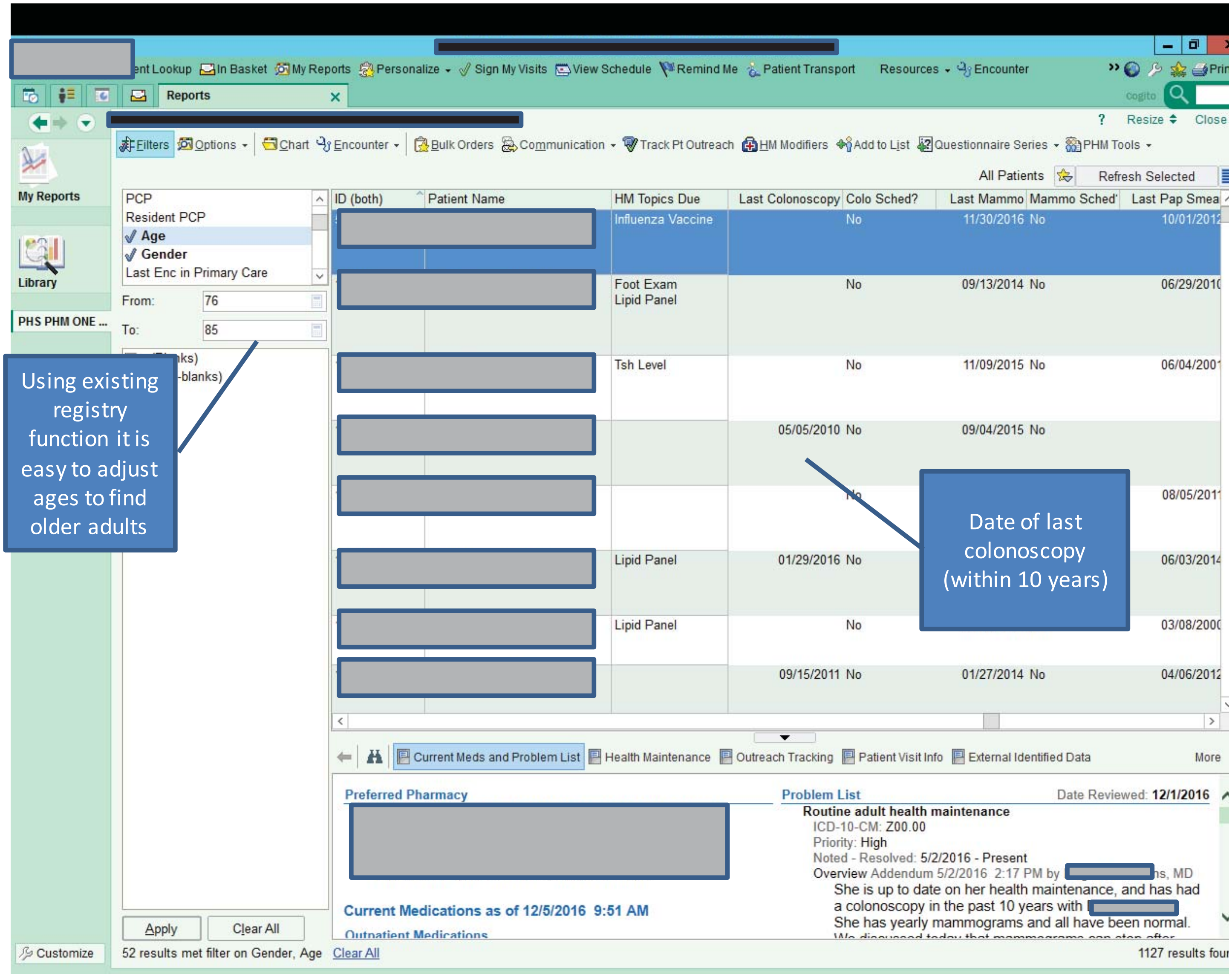

Appendix B: Screen shots from existing SDM skills webinar and detailed outline for content to be included in adapted webinar

The webinar contains didactic presentation with scripts for clinicians to try with patients across a range of clinical scenarios. The section on eliciting goals and preferences includes overarching direction as well as guidelines for specific questions to ask.

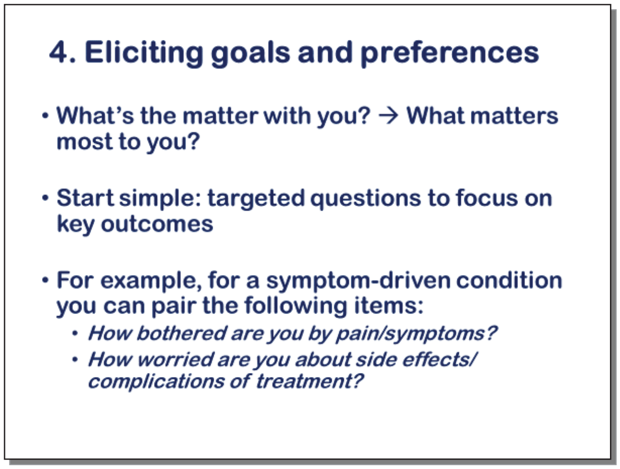

This section also provides practical techniques for more challenging situations. Here, clinicians use an adapted version of 'backcasting' to help women explore their preferences about starting mammograms at age 40 or 50. A key issue is how concerned they might be about missing a cancer. Women's responses can help identify those patients who prefer to postpone or defer screening. This technique can be applied to many clinical decisions, including colorectal cancer screening.

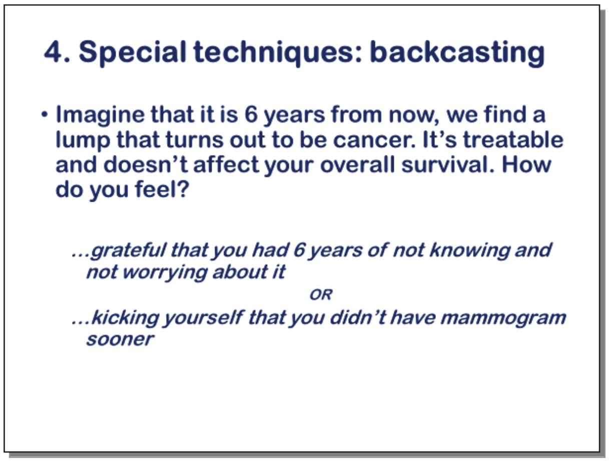

The webinar also has embedded case studies with videos illustrating shared decision making conversations between primary care clinicians and patients. Users have a score sheet to rate the presence and quality of different shared decision making behaviors in the videos.

Appendix B cont. Detailed outline for content to be included in adapted webinar

Shared Decision Making Skills Training Content
Colorectal Cancer (CRC) Screening Discussions with Older Patients

Mode of content	Description of content
Slides with audio	Introduction to shared decision making (key concepts and steps)
Interactive questions	Short assessment: understanding of key SDM concepts, when it is appropriate to use SDM, and barriers to use
Video	Video case 1: Visit with 83 year old woman who is frail and due for colonoscopy, her mother was diagnosed with colon cancer at age 90, her prior tests have been negative. She is insistent on continuing screening.
Interactive questions	*Users score case for presence/absence of SDM behaviors*<br>Faculty debrief on case what went well, what was missing from SDM perspective
Slides with audio	Communicating evidence to older patients<br>• Incidence and risk factors for CRC<br>• Clinical Guidelines for Colorectal Cancer Screening for patients 76-85<br>• Benefits and harms of colonoscopy, stool based tests, no screening<br>• Consideration of co-morbidities, functional status, life expectancy<br>• Benefits and harms of treatment for CRC for patients 76-85
Interactive questions	Short assessment: understanding of evidence, clinical guidelines, limitations in evidence base for older adults
Slides with audio	Eliciting Patients' Goals, Concerns and Preferences<br>• Reasons to screen (e.g. reducing cancer mortality, benefits of early detection)<br>• Reasons not to screen (e.g. concerns with complications, false positives, other health issues)<br>• Challenges and barriers to implement CRC screening tests<br>• Involvement of spouse or others in decision making
Interactive questions	Short assessment: understanding of patients' goals and concerns regarding screening, techniques for assisting in deliberation for those who are unsure, techniques to handle conflicting opinions (doctor vs patient, patient vs spouse).
Video	Video case 2: Visit with 81 year old man who has heart disease and prior total knee replacement and spine surgery, he had pre-cancerous polyp removed on prior colonoscopy. He is reluctant to go through another prep and colonoscopy.
Interactive questions	*Users score case for presence/absence of SDM behaviors*<br>Faculty debrief case, what went well, what was missing from SDM perspective
Slides with audio	Special issues (users can pick two or more):<br>• Low literacy and limited English proficiency populations<br>• Patients with mild cognitive impairment and caregiver involvement<br>• Using patient decision aids and risk calculators<br>• Patients >75 who have never been screened<br>• Quality measures, documentation, and medical malpractice risk
Interactive questions	Short assessment at end of each section to check understanding of and ability to apply concepts in each area
Slides with audio	Summary of key points, recap SDM steps, and evaluation

Appendix C: Site specific recruitment data

As described in the research plan, we will recruit clinicians from 45 primary care practices affiliated with three organizations, Massachusetts General Hospital, Maine Medical Center and Partners Community Physicians' Organization. The eligibility criteria for clinicians and patients are included in **Section D5** and in **Human Subjects Plan**. We have used similar assumptions for response rates across sites. Administrative data indicates that there are 190 primary care clinicians at MGH, 108 at MMC and 128 at the PCPO sites. Investigators plan to target clinicians who have a large potentially eligible patient population and those who see a diverse population of patients (estimate 130 at MGH, 70 at MMC, and 80 at PCPO). With a conservative response rate estimate for clinicians (50%), we will need to invite 120 clinicians across the three sites in order to have 60 indicate interest in participating, and then assume that 50 (85%) of those will complete baseline assessment and enroll on the trial. We have sufficient clinician population to meet the recruitment targets.

Administrative data suggests that the 50 participating clinicians will have on average, 70 patients in their panel that are 76-85, which gives 50X70=3500 potentially eligible patients (2100 at MGH, 700 at MMC and 700 at PCPO). Using data from EHR and from our GI colleagues (distribution of colonoscopy recall rates for 10-year, 5-year and 3-year recalls for patients 76-85), we estimate that there will be 17 eligible patients per clinician over the study period. We will approach (50X17=830) 830 patients, and estimate that roughly 60% will complete the post-visit survey. This response rate will result in 500 completed surveys, or approximately 250 per arm. The site-specific numbers for clinician and patient participants are in Tables 3a, 3b and 3c.

Table 3a. Recruitment Plan for MGH

	Clinicians	Patients
1. Estimated number of potentially eligible study participants (Clinicians: administrative data; Patients: EHR and admin data)	190	2100
2. Total number of study participants expected to be screened:	190	2100
3. Total number of study participants expected to be eligible of those screened:	130	510
4. Target sample size (use same number stated in milestones):	30	300
5. If applicable, total number of practices or centers that will enroll participants:	19	19
6. Projected month first participant enrolled (month after project initiation):	6	9
7. Projected month last participant enrolled (month after project initiation):	9	21
8. Projected rate of enrollment (number enrolled per month of enrollment period):	10	25
9. Estimated percentage of participant dropout:	15%	n/a

Table 3b. Recruitment Plan for MMC

	Clinicians	Patients
10. Estimated number of potentially eligible study participants (Clinicians: administrative data; Patients: EHR and admin data)	108	700
11. Total number of study participants expected to be screened:	108	700
12. Total number of study participants expected to be eligible of those screened:	70	170
13. Target sample size (use same number stated in milestones):	10	100
14. If applicable, total number of practices or centers that will enroll participants:	10	10
15. Projected month first participant enrolled (month after project initiation):	6	9
16. Projected month last participant enrolled (month after project initiation):	9	21
17. Projected rate of enrollment (number enrolled per month of enrollment period):	3-4	8-9
18. Estimated percentage of participant dropout:	15%	n/a

Table 3c. Recruitment Plan for PCPO

	Clinicians	Patients
19. Estimated number of potentially eligible study participants (Clinicians: administrative data; Patients: EHR and admin data)	128	700
20. Total number of study participants expected to be screened:	128	700
21. Total number of study participants expected to be eligible of those screened:	80	170
22. Target sample size (use same number stated in milestones):	10	100
23. If applicable, total number of practices or centers that will enroll participants:	16	16
24. Projected month first participant enrolled (month after project initiation):	6	9
25. Projected month last participant enrolled (month after project initiation):	9	21
26. Projected rate of enrollment (number enrolled per month of enrollment period):	3-4	8-9
27. Estimated percentage of participant dropout:	15%	n/a

PCORI Methodology Standards Checklist

Follow the instructions provided below. Upload the completed template as a single PDF into PCORI Online. Detailed instructions are included in the Application Guidelines for this PCORI Funding Announcement (PFA). Refer to the PCORI Methodology Report for explanations about the standards. Note that the Methodology Standards in red text indicate those that are newly approved, as of May 8, 2017 by the Board of Governors.

In the checklist below, you will see a complete list of the PCORI Methodology Standards. In column D, using the drop-down menu options, indicate whether or not each methodology standard applies to your research. If the standard applies, in column E, provide the page number and section of your research plan where the text illustrates how you addressed the standard. Lastly, in column F, indicate whether your study may deviate from the standard and provide a rationale. Repeat the sequence for each standard.

Application ID	
PI Name	
Application Title	

Standard Category	Abbrev.	Standard	Have you addressed how you plan to adhere to the standard in your application?	List page numbers	Notes	
**Cross-Cutting Standards for PCOR**						
Standards for Formulating Research Questions	RQ-1	Identify Gaps in Evidence	Yes	4	Section B	
	RQ-2	Develop a Formal Study Protocol	Yes	6	Section D4a	
	RQ-3	Identify Specific Populations and Health Decision(s) Affected by the Research	Yes	2, 4	Sections B and C	
	RQ-4	Identify and Assess Participant Subgroups	Yes	9-10; 10-11	Sections D7 and D9	
	RQ-5	Select Appropriate Interventions and Comparators	Yes	5	Section D2	
	RQ-6	Measure Outcomes that People Representing the Population of Interest Notice and Care About	Yes	9	Section D7	
Standards Associated with Patient-Centeredness	PC-1	Engage people representing the population of interest and other relevant stakeholders in ways that are appropriate and necessary in a given research context.	Yes	7	Section D4b	
	PC-2	Identify, Select, Recruit, and Retain Study Participants Representative of the Spectrum of the Population of Interest and Ensure that Data Are Collected Thoroughly and Systematically from All Study Participants	Yes	6	Sections D3 and D4a	
	PC-3	Use Patient-Reported Outcomes When Patients or People at Risk of a Condition Are the Best Source of Information for Outcomes of Interest	Yes	9	Section D7	
	PC-4	Support dissemination and implementation of study results	Yes	12, 15	Sections 10c and Dissemination Plan	
Standards for Data Integrity and Rigorous Analyses	IR-1	A priori, Specify Plans for Data Analysis that Correspond to Major Aims	Yes	10 and 11	Section D9	
	IR-2	Assess Data Source Adequacy	Yes	10	Section D8	
	IR-3	Describe Data Linkage Plans, if applicable	Yes	10	Section D8	
	IR-4	Document Validated Scales and Tests	Yes	9	Section D7	
	IR-5	Provide Sufficient Information in Reports to Allow for Assessments of the Study's Internal and External Validity	Yes	4, 6	Sections B and D4a	
	IR-6	Masking should be used when feasible	Yes	6	Section D3	
Standards for Preventing and Handling Missing Data	MD-1	Describe in Protocol Methods to Prevent and Monitor Missing Data	Yes	6	Section D4a	
	MD-2	Use Validated Statistical Methods to Deal with Missing Data that Properly Account for Statistical Uncertainty Due to Missingness	Yes	10	Section D9	
	MD-3	Record and Report All Reasons for Dropout and Missing Data, and Account for All Patients in Reports	Yes	10	Section D9	
	MD-4	Examine Sensitivity of Inferences to Missing Data Methods and Assumptions, and Incorporate into Interpretation	Partially	10	Section D9. Methods for sensitivity analsyses for missing data will be included in the full study protocol (they were not included here due to page limits for research plan)	
Standards for Heterogeneity of Treatment Effect (HTE)	HT-1	State the Goals of HTE Analyses, including hypotheses and the supporting evidence base	Yes	11	Section D9	
	HT-2	For all HTE Analyses, provide an analysis plan, including the use of appropriate statistical methods.	Yes	11	Section D9	
	HT-3	Report all prespecified HTE analyses and, at minimum, the number of post-hoc HTE analyses, including all subgroups and outcomes analyzed.	Yes	11	Section D9	
**Standards for Specific Study Designs and Methods**						
Standards for Data Registries	DR-1	Requirements for the Design of Registries	N/A			
	DR-2	Documentation and reporting requirements of registry materials, characteristics, and bias	N/A			
	DR-3	Adapting established registries for PCOR	N/A			
	DR-4	Documentation requirements when using registry data	N/A			
Standards for Data Networks as Research-Facilitating Structures	DN-1	Requirements for the Design and Features of Data Networks	N/A			
	DN-2	Selection and Use of Data Networks	N/A			
Causal Inference Standards	CI-1	CI-I: Specify the causal model underlying the research question ***CROSS-CUTTING STANDARD***	Yes	4 and 5	Section D1	
	CI-2	Define and appropriately characterize the analysis population used to generate effect estimates.	Yes	6, 8-10	Sections D4a, D5, D7 and D9	
	CI-3	Define with the appropriate precision the timing of the outcome assessment relative to the initiation and duration of exposure.	Yes	6	Section D4a	
	CI-4	Measure potential confounders before start of exposure and report data on potential confounders with study results.	Yes	9	Section D7	
	CI-5	Report the assumptions underlying the construction of Propensity Scores and the comparability of the resulting groups in terms of the balance of covariates and overlap	N/A			

Standard Category	Abbrev.	Standard	Have you addressed how you plan to adhere to the standard in your application?	List page numbers	Notes	
	CI-6	Assess the Validity of the Instrumental Variable (i.e. how the assumptions are met) and report the balance of covariates in the groups created by the instrumental variable	N/A			
Standards for Adaptive and Bayesian Trial Designs	AT-1	Specify planned adaptations, decisional thresholds, and statistical properties of those adaptations.	N/A			
	AT-2	Specify the structure and analysis plan for Bayesian adaptive randomized clinical trial designs	N/A			
	AT-3	Ensure that clinical trial infrastructure is adequate to support planned adaptation(s) and independent interim analyses.	N/A			
	AT-4	When reporting adaptive randomized clinical trials, use the CONSORT statement, with modifications.	N/A			
Standards for Studies of Medical Tests	MT-1	Specify the clinical context and key elements of the medical test	N/A			
	MT-2	Assess the effect of factors known to affect performance and outcomes	N/A			
	MT-3	Focus studies of medical tests on patient-centered outcomes, using rigorous study designs with a preference for randomized controlled trials	N/A			
Standards for Systematic Reviews	SR-1	Adhere to National Academy of Medicine (NAM) standards for systematic reviews of comparative effectiveness research, as appropriate.	N/A			
Standards on Research Designs Using Clusters	RC-1	Specify whether the study objectives, the interventions, and the primary outcomes pertain to the cluster level or the individual level.	Yes	10 and 11	Section D9	
	RC-2	Justify the choice of cluster randomization	Yes	6	Section D3	
	RC-3	Power and sample size estimates must use appropriate methods to account for the dependence of observations within clusters and the degrees of freedom available at the cluster level.	Yes	8-9; 10-11	Sections D6 and D9	
	RC-4	Data analyses must account for the dependence of observations within clusters regardless of its magnitude.	Yes	10 and 11	Section D9	
	RC-5	Stratified randomization should be used when feasible	Yes	6	Section D3	
