# Supplementary material for: Shared Decision-Making in Colorectal Cancer Screening for Older Adults: A Secondary Analysis of a Cluster Randomized Clinical Trial
Source: JAMA Netw Open. 2024 Aug 23;7(8):e2429645. doi: 10.1001/jamanetworkopen.2024.29645 (PMC11344228; doi:10.1001/jamanetworkopen.2024.29645)
Supplement: Supplement 2. — eTable 1. Shared Decision-Making Training Course Content and Sample Reminder Notification eTable 2. Respondents vs Nonrespondents (Including Those Who Opted Out Prior to the Visit) eTable 3. Physician Characteristics for the Study Groups [file jamanetwopen-e2429645-s002.pdf]

Supplementary Online Content

Sepucha KR, Chang Y, Valentine KD, et al. Shared decision-making in colorectal cancer screening for older adults: a secondary analysis of a cluster randomized clinical trial. *JAMA Netw Open*. 2024;7(8):e2429645. doi:10.1001/jamanetworkopen.2024.29645

eTable 1. Shared Decision-Making Training Course Content and Sample Reminder Notification

eTable 2. Respondents vs Nonrespondents (Including Those Who Opted Out Prior to the Visit)

eTable 3. Physician Characteristics for the Study Groups

This supplementary material has been provided by the authors to give readers additional information about their work.

© 2024 Sepucha KR et al. *JAMA Network Open.*

eTable 1. Shared Decision-Making Training Course Content and Sample Reminder Notification

**Course Content**	
Section 1	• Review clinical guidelines for Colorectal cancer screening for patients aged 76-85<br>• Overview of shared decision making and 7 Steps Framework<br>• Scoring two video vignettes one positive and one negative (Case: Mr. Sullivan) for elements of shared decision making
Section 2	• In depth description of 7 Steps for Shared decision making with example scripts for each step:<br>1. Invite participation<br>2. Present Options<br>3. Describe Benefits and Harms<br>4. Elicit goals and concerns<br>5. Facilitate deliberation<br>6. Support implementation<br>7. Involve trusted others<br>• Scoring two video vignettes (Case: Mrs. Turner) for elements of shared decision making<br>• Presentation of two risk calculators to estimate colorectal cancer risk and overall life expectancy
Section 3	Interactive case-based module where learners progress through cases and determine (1) whether or not the patient is eligible/appropriate for the decision discussion and (2) for the two eligible cases (Case: Mrs. Clark and Mr. Martinez) the learner progresses through the 7 steps of shared decision making for each case, including accessing the online risk calculators to estimate CRC risk and life expectancy, with tips and feedback.
**Case descriptions**	
Mr. Sullivan	81 year-old man with heart disease and arthritis with prior spine and hip surgery. He had an abnormal polyp 6 years ago and is over due for follow-up colonoscopy. He asks whether he really needs one at his age. His wife wants him to get one.
Mrs. Turner	76 year-old woman, recently moved to town to be closer to her daughter. She is new to the practice, is very healthy, but has never been screened before (prior PCP notes mention she declined colonoscopy).
Ms. Clark	83 year-old woman with rheumatoid arthritis who is extremely frail and lives alone. Her mother was diagnosed with colon cancer at 90. All of her past colonoscopies were normal. Her last one was at age 73 and she wants to schedule another one.

© 2024 Sepucha KR et al. *JAMA Network Open.*

<table>
<tr><td>Mr. Martinez</td><td>78-year-old man who has hypertension and high cholesterol. He is moderately active and has no family history of CRC. His first colonoscopy (age 56) was normal, his second (age 66) removed single tubular adenoma and his third (age 71) was normal.</td></tr>
<tr><td colspan="2">Reminder notification</td></tr>
<tr><td colspan="2">«First_Name» «Last_Name» with a visit on «VisitScheduledDate» at «ApptTime» has been invited into the PRIMED Study.

Per Epic records, the patient's most recent colorectal cancer screening: «LastScreeningType» on «LastScreeningDate» / no test on file.

Please have a conversation about colorectal cancer screening at this visit.

Look out for a brief survey link after the visit.

Thank you,

<<Study research coordinator name>>

PRIMED Study information:

Eligible patients are between 76-85 years of age, and per our review of Epic these patients do not have a diagnosis of colon cancer, inflammatory bowel disease, or a serious illness for which you have previously indicated that they should not have a colon cancer screening discussion.

We recognize that many topics may be covered during your upcoming visit with your patient, and that colon cancer screening is only one of the many topics you may be discussing. We are sending this notice as a reminder that your patient may be due for an update of their colon cancer screening plan.</td></tr>
</table>

© 2024 Sepucha KR et al. *JAMA Network Open.*

eTable 2. Respondents vs Nonrespondents (Including Those Who Opted Out Prior to the Visit)

		All		Intervention		Comparator	
		Responder		Responder		Responder	
		No	Yes	No	Yes	No	Yes
N		440	466	188	236	252	230
Age [a,b]	Mean (SD)	79.8 (2.8)	79.4 (2.8)	79.8 (2.8)	79.6 (2.8)	79.8 (2.8)	79.2 (2.8)
Gender [b]							
Female	N	259	249	114	138	145	111
	%	58.9	53.4	60.6	58.5	57.5	48.3
Male	N	181	217	74	98	107	119
	%	41.1	46.6	39.4	41.5	42.5	51.7
Language							
English	N	437	463	187	234	250	229
	%	99.3	99.4	99.5	99.2	99.2	99.6
Spanish	N	3	3	1	2	2	1
	%	0.7	0.6	0.5	0.8	0.8	0.4
Site							
1	N	169	179	66	89	103	90
	%	38.4	38.4	35.1	37.7	40.9	39.1
2	N	39	25	25	17	14	8
	%	8.9	5.4	13.3	7.2	5.6	3.5
3	N	89	108	48	59	41	49
	%	20.2	23.2	25.5	25.0	16.3	21.3
4	N	85	98	26	42	59	56
	%	19.3	21.0	13.8	17.8	23.4	24.3
5	N	58	56	23	29	35	27
	%	13.2	12.0	12.2	12.3	13.9	11.7

© 2024 Sepucha KR et al. *JAMA Network Open.*

		All		Intervention		Comparator	
		Responder		Responder		Responder	
		No	Yes	No	Yes	No	Yes
Prior Test [a, b]							
Procedures	N	249	311	116	159	133	152
	%	56.6	66.7	61.7	67.4	52.8	66.1
Stool-based tests	N	82	81	41	48	41	33
	%	18.6	17.4	21.8	20.3	16.3	14.3
None	N	109	74	31	29	78	45
	%	24.8	15.9	16.5	12.3	31.0	19.6

[a]: $p<0.05$ for overall sample; [b]: $p<0.05$ for comparator.

© 2024 Sepucha KR et al. *JAMA Network Open.*

eTable 3. Physician Characteristics for the Study Groups

Characteristics	Comparator Physicians N=31 N (%)	Intervention Physicians N=28 N (%)
Age: mean (SD)	52.4 (9.0)	53.1 (10.0)
Gender		
Female	16 (51.6%)	14 (50.0%)
Male	15 (48.4%)	15 (50%)
Years in practice: mean (SD)	20.9 (9.6)	22.4 (10.9)
Number of enrolled patients: median (range)	6 (1, 20)	8 (0, 25)
Had prior SDM training	8 (25.8%)	8 (28.6%)
Practice setting		
Academic	19 (61.3%)	18 (64.3%)
Community	12 (38.7%)	10 (35.7%)

© 2024 Sepucha KR et al. *JAMA Network Open.*
