# Supplementary material for: Shared Decision-Making in Colorectal Cancer Screening for Older Adults: A Secondary Analysis of a Cluster Randomized Clinical Trial
Source: JAMA Netw Open. 2024 Aug 23;7(8):e2429645. doi: 10.1001/jamanetworkopen.2024.29645 (PMC11344228; doi:10.1001/jamanetworkopen.2024.29645)
Supplement: Supplement 3. — Data Sharing Statement [file jamanetwopen-e2429645-s003.pdf]

Data Sharing Statement

Sepucha. Shared Decision-Making in Colorectal Cancer Screening for Older Adults. *JAMA Netw Open*. Published August 23, 2024. doi:10.1001/jamanetworkopen.2024.29645

Data
Data available: Yes
Data types: Deidentified participant data, Data dictionary
How to access data: After the study results have been published, de-identified data sets and code books will be deposited in an open access service, ICPSR (https://www.icpsr.umich.edu/icpsrweb/).
When available: With publication

Supporting Documents
Document types: None

Additional Information
Who can access the data: Anyone requesting the data who meets the requirements for ICPSR (https://www.icpsr.umich.edu/icpsrweb/) will be able to access the data.
Types of analyses: Anyone requesting the data who meets the requirements for ICPSR (https://www.icpsr.umich.edu/icpsrweb/) will be able to access the data and perform analyses.
Mechanisms of data availability: The data will be available through ICPSR (https://www.icpsr.umich.edu/icpsrweb/).
